# Comparative Efficacy of Seven Psychotherapeutic Interventions for Patients with Depression: A Network Meta-Analysis

**DOI:** 10.1371/journal.pmed.1001454

**Published:** 2013-05-28

**Authors:** Jürgen Barth, Thomas Munder, Heike Gerger, Eveline Nüesch, Sven Trelle, Hansjörg Znoj, Peter Jüni, Pim Cuijpers

**Affiliations:** 1 Institute of Social and Preventive Medicine (ISPM), University of Bern, Bern, Switzerland; 2 CTU Bern, Department of Clinical Research, University of Bern, Bern, Switzerland; 3 Faculty of Epidemiology and Population Health, London School of Hygiene and Tropical Medicine, London, United Kingdom; 4 Department of Clinical Psychology and Psychotherapy, University Bern, Bern, Switzerland; 5 Department of Clinical Psychology, VU University Amsterdam, Amsterdam, The Netherlands; Massachusetts General Hospital, United States of America

## Abstract

**Background:**

Previous meta-analyses comparing the efficacy of psychotherapeutic interventions for depression were clouded by a limited number of within-study treatment comparisons. This study used network meta-analysis, a novel methodological approach that integrates direct and indirect evidence from randomised controlled studies, to re-examine the comparative efficacy of seven psychotherapeutic interventions for adult depression.

**Methods and Findings:**

We conducted systematic literature searches in PubMed, PsycINFO, and Embase up to November 2012, and identified additional studies through earlier meta-analyses and the references of included studies. We identified 198 studies, including 15,118 adult patients with depression, and coded moderator variables. Each of the seven psychotherapeutic interventions was superior to a waitlist control condition with moderate to large effects (range *d* = −0.62 to *d* = −0.92). Relative effects of different psychotherapeutic interventions on depressive symptoms were absent to small (range *d* = 0.01 to *d* = −0.30). Interpersonal therapy was significantly more effective than supportive therapy (*d* = −0.30, 95% credibility interval [CrI] [−0.54 to −0.05]). Moderator analysis showed that patient characteristics had no influence on treatment effects, but identified aspects of study quality and sample size as effect modifiers. Smaller effects were found in studies of at least moderate (Δ*d* = 0.29 [−0.01 to 0.58]; *p* = 0.063) and large size (Δ*d* = 0.33 [0.08 to 0.61]; *p* = 0.012) and those that had adequate outcome assessment (Δ*d* = 0.38 [−0.06 to 0.87]; *p* = 0.100). Stepwise restriction of analyses by sample size showed robust effects for cognitive-behavioural therapy, interpersonal therapy, and problem-solving therapy (all *d*>0.46) compared to waitlist. Empirical evidence from large studies was unavailable or limited for other psychotherapeutic interventions.

**Conclusions:**

Overall our results are consistent with the notion that different psychotherapeutic interventions for depression have comparable benefits. However, the robustness of the evidence varies considerably between different psychotherapeutic treatments.

*Please see later in the article for the Editors' Summary*

## Abstract 2

Jürgen Barth and colleagues use network meta-analysis - a novel methodological approach - to reexamine the comparative efficacy of seven psychotherapeutic interventions for adults with depression.

*Please see later in the article for the Editors' Summary*

## Editors' Summary

**Background:**

Depression is a very common condition. One in six people will experience depression at some time during their life. People who are depressed have recurrent feelings of sadness and hopelessness and might feel that life is no longer worth living. The condition can last for months and often includes physical symptoms such as headaches, sleeping problems, and weight gain or loss. Treatment of depression can include non-drug treatments (psychotherapy), antidepressant drugs, or a combination of the two. Especially for people with mild or intermediate depression, psychotherapy is often considered the preferred first option. Psychotherapy describes a range of different psychotherapies, and a number of established types of psychotherapies have all shown to work for at least some patients.

**Why Was This Study Done?:**

While it is broadly accepted that psychotherapy can help people with depression, the question of which type of psychotherapy works best for most patients remains controversial. While many scientific studies have compared one psychotherapy with control conditions, there have been few studies that directly compared multiple treatments. Without such direct comparisons, it has been difficult to establish the respective merits of the different types of psychotherapy. Taking advantage of a recently developed method called “network meta-analysis,” the authors re-examine the evidence on seven different types of psychotherapy to see how well they have been shown to work and whether some work better than others.

**What Did the Researchers Do and Find?:**

The researchers looked at seven different types of psychotherapy, which they defined as follows. “Interpersonal psychotherapy” is short and highly structured, using a manual to focus on interpersonal issues in depression. “Behavioral activation” raises the awareness of pleasant activities and seeks to increase positive interactions between the patient and his or her environment. “Cognitive behavioral therapy” focuses on a patient's current negative beliefs, evaluates how they affect current and future behavior, and attempts to restructure the beliefs and change the outlook. “Problem solving therapy” aims to define a patient's problems, propose multiple solutions for each problem, and then select, implement, and evaluate the best solution. “Psychodynamic therapy” focuses on past unresolved conflicts and relationships and the impact they have on a patient's current situation. In “social skills therapy,” patients are taught skills that help to build and maintain healthy relationships based on honesty and respect. “Supportive counseling” is a more general therapy that aims to get patients to talk about their experiences and emotions and to offer empathy without suggesting solutions or teaching new skills.

The researchers started with a systematic search of the medical literature for relevant studies. The search identified 198 articles that reported on such clinical trials. The trials included a total of 15,118 patients and compared one of the seven psychotherapies either with another one or with a common “control intervention”. In most cases, the control (no psychotherapy) was deferral of treatment by “wait-listing” patients or continuing “usual care.” With network meta-analysis they were able to summarize the results of all these trials in a meaningful way. They did this by integrating direct comparisons of several psychotherapies within the same trial (where those were available) with indirect comparisons across all trials (using no psychotherapy as a control intervention).

Based on the combined trial results, all seven psychotherapies tested were better than wait-listing or usual care, and the differences were moderate to large, meaning that the average person in the group that received therapy was better off than about half of the patients in the control group. When comparing the therapies with each other, the researchers saw small or no differences, meaning that none of them really stood out as much better or much worse than the others. They also found that the treatments worked equally well for different patient groups with depression (younger or older patients, or mothers who had depression after having given birth). Similarly, they saw no big differences when comparing individual with group therapy, or person-to-person with internet-based interactions between therapist and patient.

However, they did find that smaller and less rigorous studies generally found larger benefits of psychotherapies, and most of the studies included in the analysis were small. Only 36 of the studies had at least 50 patients who received the same treatment. When they restricted their analysis to those studies, the researchers still saw clear benefits of cognitive-behavioral therapy, interpersonal therapy, and problem-solving therapy, but not for the other four therapies.

**What Do these Findings Mean?:**

Similar to earlier attempts to summarize and make sense of the many study results, this one finds benefits for all of the seven psychotherapies examined, and none of them stood as being much better than some or all others. The scientific support for being beneficial was stronger for some therapies, mostly because they had been tested more often and in larger studies.

Treatments with proven benefits still do not necessarily work for all patients, and which type of psychotherapy might work best for a particular patient likely depends on that individual. So overall this analysis suggests that patients with depression and their doctors should consider psychotherapies and explore which of the different types might be best suited for a particular patient.

The study also points to the need for further research. Whereas depression affects large numbers of people around the world, all of the trials identified were conducted in rich countries and Western societies. Trials in different settings are essential to inform treatment of patients worldwide. In addition, large high-quality studies should further explore the potential benefits of some of therapies for which less support currently exists. Where possible, future studies should compare psychotherapies with one another, because all of them have benefits, and it would not be ethical to withhold such beneficial treatment from patients.

**Additional Information:**

Please access these Web sites via the online version of this summary at http://dx.doi.org/10.1371/journal.pmed.1001454.

- The US National Institute of Mental Health provides information on all aspects of depression (in English and Spanish); information on psychotherapy includes information on its most common forms
- The UK National Health Service Choices website also provides detailed information about depression and includes personal stories about depression
- The UK nonprofit Mind provides information on depression, including an explanation of the most common psychotherapies in the UK
- MedlinePlus provides links to other resources about depression (in English and Spanish)
- The UK nonprofit healthtalkonline.org has a unique database of personal and patient experiences on depression

## Introduction

Depressive disorders are very common; about one-fifth of the population will be affected in their lifetime in high-income countries [1],[2]. Major depression ranks fourth on the list of disorders with the highest burden of disease worldwide, and it is expected to be ranked first in high-income countries by 2030 [3]. Depressive disorders can lower the ability to function in work and daily life [4].

Various psychotherapeutic interventions have been developed to treat depression, including cognitive-behavioural, interpersonal, humanistic, and psychodynamic approaches. There is substantial evidence that many types of psychotherapeutic interventions have a moderate to large effect (*d* = −0.66, 95% CI [−0.73 to −0.60] [5]), when compared to control conditions. Substantial effects compared to control conditions have been documented specifically for behavioural activation [6],[7], cognitive-behavioural therapy [8],[9], interpersonal psychotherapy [10],[11], problem-solving therapy [12], and short term psychodynamic therapy [13],[14]. Different modalities and forms of psychotherapeutic intervention have similar benefits and no difference has yet been found between individual or group treatment formats [15],[16]. Similarly, it seems to make little difference whether psychotherapeutic interventions on depression are provided face-to-face, via telephone, or on the Internet [17].

While there is broad consensus that psychotherapeutic interventions are beneficial for depressed patients, there is an ongoing debate about the comparative efficacy of different psychotherapeutic interventions. Meta-analyses of within-study comparisons of different types of psychotherapeutic interventions for depression are varied in their conclusions. Two meta-analyses found cognitive-behavioural therapy to be more effective than non-cognitive-behavioural interventions [9],[18] in direct comparisons. In contrast, a meta-analytic comparison of cognitive-behavioural therapy and short-term psychodynamic therapy for depression found no significant differences in efficacy between these interventions [14].

In the most comprehensive investigation of the relative efficacy of psychotherapeutic interventions for depression to date, Cuijpers and colleagues [19] synthesized 53 studies containing within-study comparisons of different psychotherapeutic interventions. The effects of cognitive-behavioural therapy, psychodynamic therapy, behavioural activation treatment, problem-solving therapy, and social skills training did not differ significantly from other psychotherapeutic interventions. Interpersonal therapy was found to be somewhat more effective (*d* = −0.21, CI 95% [−0.42 to −0.01]), and supportive counselling was found to be somewhat less effective than other psychotherapeutic interventions (*d* = 0.17, CI 95% [−0.32 to −0.03]). However, in this meta-analysis, the relative effects of psychotherapeutic interventions were established by pooling all studies that compared the respective treatment to any other intervention. This method complicates the interpretation of the results because the pooled comparator interventions compile different psychotherapeutic interventions. However, this approach had to be used since the number of available within-study comparisons of two specific interventions (e.g., interpersonal psychotherapy versus psychodynamic therapy) was limited.

We employed network meta-analysis—a methodological approach that allows for the comparison of a variety of psychotherapeutic interventions head to head or with a control condition [20]—to overcome the restrictions of limited available comparisons and the problem of lumping together different psychotherapeutic interventions. In network meta-analyses, the information available from within-study comparisons of treatment A and treatment B is combined with indirect comparisons of A and B derived from studies that compare either of the two treatments with a common comparator C (either a third psychotherapeutic intervention or a control condition). Network meta-analysis has already been used to investigate pharmacological treatments for depression [21] and mania [22], but has not yet been used to our knowledge in psychotherapy research.

The quality of primary studies potentially threatens the validity of meta-analyses [23]–[25]. Inadequate concealment of allocation, exclusion of patients from the analysis (i.e., if analysis is not intention-to-treat), and lack of blinding of outcome assessors [26]–[28] are known to have a biasing effect. Furthermore, evidence suggests that treatment efficacy is overestimated in studies with small sample size [29].

We aimed to re-examine the comparative efficacy of different psychotherapeutic interventions for adult depression by using network meta-analysis to integrate all available information from randomized controlled studies. We also wanted to assess the influence of study quality, sample size, and clinical characteristics on effect estimates (for a protocol of the network meta-analysis see [30]).

## Methods

### Study Selection and Inclusion Criteria

We used a database of randomized controlled trials on the efficacy of psychotherapeutic interventions of adult depression (www.evidencebasedpsychotherapies.org), which has been described in detail elsewhere [5],[31]. The database was developed through a comprehensive literature search (from 1966 to November 1, 2012) in PubMed, PsycINFO, Embase, and the Cochrane Central Register of Controlled Trials. Controlled vocabulary and text words related to different psychotherapeutic interventions and depression were used in the search. In the WHO Afro Library we used the text word depression for our search. We obtained all primary studies from 42 meta-analyses of psychotherapeutic interventions for depression [32] and checked the references of the included studies.

We included in this meta-analysis only studies with a randomised design. Studies of adults with a depressive disorder, or with elevated levels of depressive symptoms were required to compare the effects of a psychotherapeutic intervention to a control condition (i.e., waitlist, usual care, or placebo), or to another psychotherapeutic treatment. Psychotherapeutic interventions were defined as interventions with a primary focus on language-based communication between a patient and a therapist, or as bibliotherapy supported by a therapist. We included psychotherapeutic interventions from seven pre-specified categories (see below), defined for a previous meta-analysis [19]. No restrictions were made based on format (individual or group) or treatment setting (face to face, telephone, or internet). Combinations of psychotherapeutic interventions with pharmacotherapy or other non-psychotherapeutic interventions (e.g., managed care interventions and disease management programmes) were excluded. Eligible control conditions were waitlist, usual care, and (psychological or pill) placebo. Comparisons of a psychotherapeutic intervention with pharmacotherapy or other non-psychotherapeutic interventions were excluded. We also excluded studies on maintenance treatment and relapse prevention, and studies that included participants who were anxious but not depressed at the time-point of inclusion. Studies were eligible irrespective of the inclusion of patients with comorbid general medical or psychiatric disorders. No language restrictions and restrictions on publication type were applied.

### Psychotherapeutic Interventions

Psychotherapeutic interventions were coded according to type of intervention and treatment format. Based on an expert taxonomy of psychotherapy for depression [19], we classified psychotherapy into seven different types: interpersonal therapy, behavioural activation, cognitive-behavioural therapy, problem solving therapy, social skills training, psychodynamic therapy, and supportive counselling. A description of each type of intervention is presented in Table 1. We coded further, on the basis of whether a psychotherapeutic intervention was delivered individually, face-to-face, or in a different setting (e.g., group psychotherapeutic intervention, internet based individual treatment, bibliotherapy). The number of sessions was used to rate the treatment dose as low (six or fewer sessions) or high (more than six sessions).

**Table 1:** Description of intervention strategies.

Type of Psychotherapeutic Intervention	Description
Interpersonal psychotherapy (IPT)	IPT is a brief and highly structured manual-based psychotherapy that addresses interpersonal issues in depression to the exclusion of all other foci of clinical attention (http://www.interpersonalpsychotherapy.org). IPT has no specific theoretical origin, although its theoretical basis can be seen as coming from the work of Sullivan, Meyer, and Bowlby. The current form of the treatment was developed by the late Gerald Klerman and Myrna Weissman in the 1980s [50].
Behavioural activation (ACT)	We considered an intervention to be activity scheduling when the registration of pleasant activities and the increase of positive interactions between a person and his or her environment were the core elements of the treatment. Social skills training could be a part of the intervention. Although this intervention was developed by Lewinsohn [51], we also included studies that used the principles of this intervention but did not refer directly to the work of Lewinsohn and colleagues [51]. Some studies referred to the behavioural activation component included in the manual for CBT by Beck et al. [52]. This component of CBT is based on similar principles.
Cognitive behavioural therapy (CBT)	In CBT, therapists focus on the impact a patient's present dysfunctional thoughts have on current behaviour and future functioning. CBT is aimed at evaluating, challenging, and modifying a patient's dysfunctional beliefs (cognitive restructuring). In this form of treatment, the therapist mostly emphasizes homework assignments and outside-of-session activities. Therapists exert an active influence over therapeutic interactions and topics of discussion, use a psychoeducational approach, and teach patients new ways of coping with stressful situations.
Problem-solving therapy (PST)	We defined PST as a psychological intervention in which the following elements had to be included: definition of personal problems, generation of multiple solutions to each problem, selection of the best solution, the working out of a systematic plan for this solution, and evaluation as to whether the solution has resolved the problem. There are several subtypes of PST, such as PST according to Nezu [53] and Mynors-Wallis et al. [54], but the number of studies for each of these subtypes was too small to include in this meta-analysis.
Psychodynamic therapy (DYN)	The primary objective in (short-term) psychodynamic therapy is to enhance the patient's understanding, awareness, and insight about repetitive conflicts (intrapsychic and intrapersonal). An assumption in DYN is that a patient's childhood experiences, past unresolved conflicts, and historical relationships significantly affect a person's present life situation. In this form of treatment, the therapist concentrates on the patient's past, unresolved conflicts, and historical relationships and the impact these have on a patient's present functioning. Furthermore, in DYN the therapists explore a patient's wishes, dreams, and fantasies. The time limitations and the focal explorations of the patient's life and emotions distinguish DYN from psychoanalytic psychotherapy
Social skills training (SST)	SST is a form of behavioural therapy in which clients are taught skills that help in the building and retainment of social and interpersonal relationships. In most versions of SST, patients are trained in assertiveness. This means that the client is taught to stand up for his or her rights by expressing feelings in an honest and respectful way that does not insult people
Supportive counselling (SUP)	We defined supportive counselling as any unstructured therapy without specific psychological techniques other than those common to all approaches, such as helping people to ventilate their experiences and emotions and offering empathy. It is not aimed at solutions or acquiring new skills. It is based on the assumption that relief from personal problems may be achieved through discussion with others. These nondirective therapies are commonly described in the literature as either counselling or supportive therapy.

### Study Characteristics

We coded inclusion in the study of depressed adults in general, as opposed to specific populations (elderly people, women with postpartum depression, patients with somatic illness, or student populations). We used the procedure for diagnosing depressive symptoms to distinguish trials of patients with a formal diagnosis (e.g., according to DSM) from trials of patients with a probable diagnosis of depression (e.g., by using screenings).

Concealment of allocation, outcome assessment, and type of analysis were coded as components of study quality. Concealment of allocation was considered adequate if the investigators responsible for the selection of patients could not foresee a patient's allocation (e.g., by using external randomisation, or sealed, opaque, and sequentially numbered assignment envelopes). Any procedures based on predictable generation of allocation sequences (e.g., alternation), or potentially transparent attempts to conceal allocation, such as non-opaque envelopes, were considered inadequate. Outcome assessment was considered adequate if self-report measures or outcome assessors were blinded towards patients' treatment condition. Outcome assessment was regarded as inadequate if clinical raters were not blind. Because blinding therapists and patients is not possible in psychotherapy trials, we did not assess blinding towards treatment delivery. Type of analysis was considered adequate if all randomised patients were included (i.e., an intention-to-treat approach was used), and inadequate if some randomised patients were excluded from the analysis, or if the analysis was based only on those who completed treatment. Concealment of allocation, outcome assessment, and type of analysis were regarded as inadequate in cases where specific information was unavailable.

Studies were classified in three groups according to average number of patients per condition. We distinguished studies with <25 patients per group (small), from those with 25 to <50 patient per group (moderate), and ≥50 patients per group (large). All coding was done in duplicate by two independent raters. Disagreements were resolved by consensus.

### Statistical Analysis

A Cohen's *d* effect size with Hedges's correction for small sample bias was calculated for all comparisons contained in the studies [33]. A *d* = 0.20 represented a small, 0.50 a moderate, and 0.80 a large difference between interventions [34]. If means (Ms) and standard deviations (SDs) were not provided, we calculated them from standard errors, confidence intervals, or other statistical indices as described elsewhere [35],[36]. Depressive symptoms at post treatment were used as outcome. When the results of post treatment measurement were not reported, we extracted the results of the earliest follow-up measurement. Results from intention-to-treat analysis were preferred over results from completer analyses. All standardised self-report and observer-rated instruments measuring depressive symptoms were extracted. If results for more than one instrument were reported, we calculated the mean of the effect sizes, so that each comparison of conditions contributed only one effect size to the analyses.

To allow comparisons of conventional meta-analyses with results from subsequent network meta-analyses, we first calculated pair-wise meta-analyses of all within-study comparisons available for each contrast, using a Bayesian random effects model based on minimally informative prior distributions [37]. For the network meta-analysis, we used an extension of this model to compare various interventions [20],[38]. The model allows comparison of all conditions evaluated in a connected network of studies, and accounts for multiple comparisons from one study. The code is available in Text S1 in Supporting Information S1.

Relative effect sizes between different psychotherapeutic interventions and between psychotherapeutic interventions and control groups were estimated from the median of the posterior distribution. Corresponding 95% credibility intervals (CrI) were estimated from the 2.5th and 97.5th percentiles of the posterior distribution. In the presence of minimally informative priors, CrIs can be interpreted similarly to confidence intervals, and conventional levels of statistical significance at a two-sided *p*<0.05 can be assumed if 95% CrIs do not include 0.

The variance estimate τ^2^ as a measure of between-study heterogeneity was derived from the median observed in the posterior distribution. Tau (square root of τ^2^) represents the standard deviation of the underlying distribution from which the included trials are assumed to be a random sample from. On the basis of our definition of small, moderate, and large differences between interventions we interpreted τ^2^ as follows: τ^2^ = (0.2/2)^2^ = 0.01 was considered to represent low heterogeneity, τ^2^ = 0.0625 [(0.5/2)^2^] moderate heterogeneity, and τ^2^ = 0.16 [(0.8/2)^2^] high heterogeneity between studies. Differences between direct estimates (e.g., based on all available within-study comparisons) and indirect estimates (e.g., via shared comparators) were calculated to estimate inconsistency as previously described [39].

To determine if the results were affected by treatment format, target group, diagnosis, treatment dose, concealment of allocation, type of analysis, outcome assessment, and study size, we performed network meta-analyses stratified by these study characteristics. Two different stratifications were made for sample size based on the cut-offs defined above. The first stratification compared results from small studies to results from moderate and large studies. The second stratification compared the results from small and moderate studies to the results of large studies to analyse the effect of restricting studies according to sample size. To determine whether sample size was related to study quality, we used sample size as an ordinal variable (small, moderate, and large) and study quality indicators as dichotomous items and calculated Somer's *D*—a correlation coefficient for a dichotomous and an ordinal variable. We quantified the size of the interaction for all stratified analyses and provided corresponding *p*-values. Given the scarcity of the data, we were only able to run a model that assumed that the interaction effect across all comparisons (mean bias) was the same size.

For all Bayesian analyses, Markov-chain-Monte-Carlo methods were used. Initially we had run analyses with three chains to determine the burn-in.

Convergence of Markov chains was considered achieved when plots of the Gelman-Rubin statistics indicated that widths of pooled runs and individual runs stabilised around the same value and their ratio was around one [40]. Finally, all analyses were run using only one chain. We carried out 100,000 iterations. The first 50,000 were discarded after the burn-in period and estimates were based on the subsequent 50,000. We used Stata release 11 (StataCorp) and WinBUGS version 1.4 (MRC Biostatistics Unit 2007) for all analyses.

## Results

**Figure 1:**
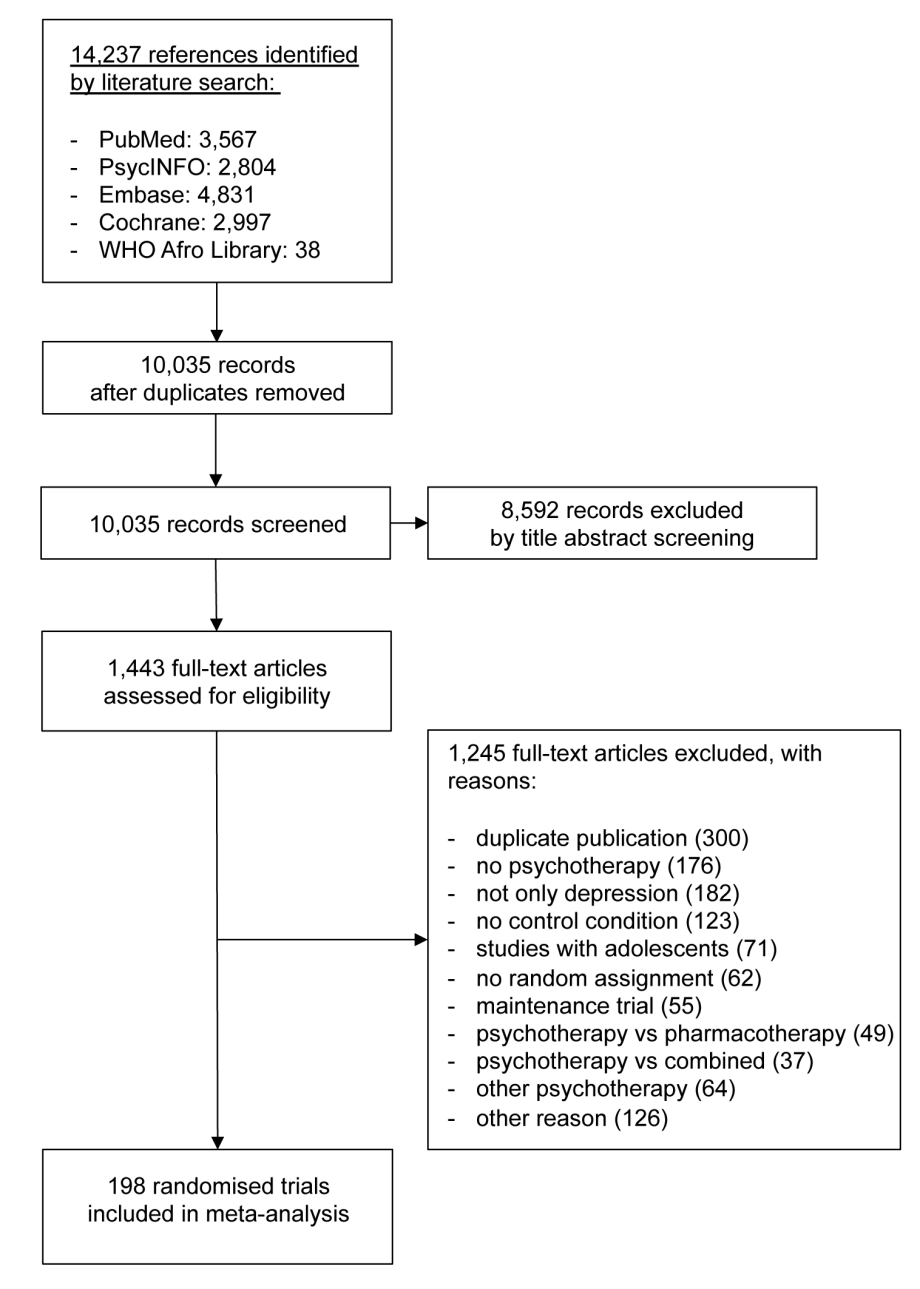
Flowchart of study inclusion.

We analysed 198 studies including 433 conditions (psychotherapeutic interventions or control) and 15,118 patients (see the flow chart in Figure 1; references of included studies are available in the Text S2 in Supporting Information S1). Sixty-three studies contained at least one comparison between two psychological interventions, and 162 studies contained comparisons of psychotherapeutic interventions with a control condition. Overall, 9,314 patients were randomised to psychotherapeutic interventions (cognitive-behavioural therapy *n* = 5,378, supportive counselling *n* = 1,125, interpersonal therapy *n* = 992, problem solving therapy *n* = 852, psychodynamic therapy *n* = 440, behavioural activation *n* = 431, social skills training *n* = 96). Another 5,805 patients were randomised to control conditions (waitlist, usual care, or placebo). The median number of patients included per treatment condition was 23 (range 5 to 418). The median publication year of studies was 2003 (range 1975 to 2012). Further descriptive information about the included studies is given in Table 2. Ninety-four studies (47%) investigated adults with depression, while 104 studies (53%) investigated depression in more specific patient populations. Of all psychotherapeutic interventions, cognitive-behavioural therapy was the intervention that was most often investigated (139 studies, 70%), while social skills training was investigated least often (seven studies, 4%). The most common control condition was waitlist (75 studies, 38%). More than half of the studies investigated psychotherapeutic interventions in an individual, face-to-face setting. Most studies were conducted in the United States (115 studies, 58%). Four studies were published in German and two studies in Spanish. Descriptive information and the coding of variables for each of the 198 included studies are provided in Tables S1 and S2 in Supporting Information S1.

**Table 2:** Summary of study characteristics across the 198 studies included in the network meta-analysis.

Descriptive Categories	Study Characteristic	Number of Studies	Percent
**Patient population**	Regular depression	94	47%
	Geriatric depression	26	13%
	Student populations	8	4%
	Women with postpartum depression	16	8%
	General medical patients with depression	27	14%
	Miscellaneous	27	14%
**Psychotherapeutic intervention** a	Interpersonal therapy	27	14%
	Behavioural activation	26	13%
	Cognitive-behavioural therapy	139	70%
	Problem solving therapy	19	10%
	Social skills training	7	4%
	Psychodynamic therapy	16	8%
	Supportive counselling	37	19%
**Control condition** b	Placebo	27	14%
	Usual care	60	30%
	Waitlist	75	38%
**Intervention format and setting**	Individual and face-to-face	97	49%
	Other	98	50%
	Mixed	2	1%
**Country**	United States	115	58%
	United Kingdom	23	11%
	Continental Europe	27	13%
	Canada	8	4%
	Australia	13	6%
	Miscellaneous	15	8%

a The percentages do not add up to 100% because many studies contained more than one treatment.

b The percentages do not add up to 100% because not all studies contained a control condition.

### Meta-Analysis of Within-Study Comparisons

The results of conventional meta-analyses (i.e., based on available within-study comparisons) for each pair of conditions are shown in the upper triangle in Table 3. Given that we had ten conditions (seven psychotherapeutic interventions and three control conditions), 45 contrasts were possible. Of 21 possible contrasts of specific psychotherapeutic interventions, there was no within-study comparison available for six of the contrasts. There was little evidence for superiority or inferiority of any of the psychotherapeutic interventions in the remaining 15 contrasts (all 95% CrIs included 0). Of 21 possible contrasts between psychotherapeutic interventions and control conditions, no within-study comparison was available for five contrasts. Supportive counselling, social skills training, problem solving, cognitive behavioural therapy, and behavioural activation were more effective than waitlist. Effect sizes were moderate to large (range *d* = −0.56 to *d* = −1.23) and significant (the 95% CrIs did not include 0). Large but non-significant effects were found for psychodynamic therapy and interpersonal therapy vis-à-vis waitlist (the 95% CrIs included 0).

**Table 3:** Relative effect sizes (and 95% credibility intervals) of psychotherapeutic interventions and control conditions from conventional meta-analysis (upper triangle) and network meta-analysis (lower triangle).

Psychotherapeutic Intervention/Control Conditions	Waitlist	Usual Care	Placebo	Supportive Counselling	Psychodynamic Therapy	Social Skills Training	Problem Solving Therapy	Cognitive-Behavioural Therapy	Behavioural Activation	Interpersonal Therapy
Waitlist		-	-	**−0.86**	−1.46	**−0.56**	**−1.23**	**−0.85**	**−1.01**	−1.38
				[−1.63 to −0.10]^a^	[−5.24 to 1.88]	[−1.06 to −0.10]	[−1.88 to −0.72]	[−0.99 to −0.72]	[−1.50 to −0.58]	[−4.55 to 1.34]
				τ^2^ = 0.241	τ^2^ = 0.260	τ^2^ = 0.056	τ^2^ = 0.329	τ^2^ = 0.147	τ^2^ = 0.143	τ^2^ = 0.215
				k = 5	k = 2	k = 5	k = 8	k = 61	k = 9	k = 2
Usual care	**−0.33**		-	**−0.42**	−0.32	-	−0.28	**−0.50**	−0.55	**−0.58**
	[−0.50 to −0.14]			[−0.61 to −0.24]	[−1.13 to 0.45]		[−0.68 to 0.09]	[−0.65 to −0.34]	[−6.90 to 5.34]	[−0.79 to −0.35]
				τ^2^ = 0.019	τ^2^ = 0.122		τ^2^ = 0.032	τ^2^ = 0.142	τ^2^ = 1.013	τ^2^ = 0.078
				k = 10	k = 3		k = 4	k = 38	k = 2	k = 13
Placebo	**−0.33**	−0.01		−0.94	−0.80	-	−0.23	**−0.49**	−0.34	−0.34
	[−0.53 to −0.13]	[−0.20 to 0.19]		[n.e.]^b^	[−2.82 to 0.98]		[−1.09 to 0.55]	[−0.65 to −0.32]	[−0.88 to 0.14]	[−0.82 to 0.14]
				τ^2^ = n.e.	τ^2^ = 0.736		τ^2^ = 0.124	τ^2^ = 0.033	τ^2^ = 0.099	τ^2^ = 0.050
				k = 1	k = 4		k = 3	k = 16	k = 5	k = 4
Supportive counselling	**−0.62**	**−0.29**	**−0.29**		−0.18	-	−0.58	−0.13	−0.38	−0.42
	[−0.82 to −0.40]	[−0.48 to −0.11]	[−0.51 to −0.06]		[−0.72 to 0.41]		[−1.44 to 0.09]	[−0.30 to 0.03]	[−1.05 to 0.27]	[−0.95 to 0.08]
					τ^2^ = 0.046		τ^2^ = 0.086	τ^2^ = 0.064	τ^2^ = 0.082	τ^2^ = 0.054
					k = 3		k = 3	k = 23	k = 4	k = 4
Psychodynamic therapy	**−0.72**	**−0.39**	**−0.39**	−0.10		−0.00	-	−0.22	−0.21	-
	[−1.02 to −0.41]	[−0.68 to −0.11]	[−0.68 to −0.09]	[−0.41 to 0.20]		[−2.32 to 2.14]		[−0.59 to 0.06]	[−0.88 to 0.45]	
						τ^2^ = 0.121		τ^2^ = 0.063	τ^2^ = 0.055	
						k = 2		k = 9	k = 3	
Social skill training	**−0.62**	−0.30	−0.30	−0.01	0.09		−0.04	0.11	0.02	-
	[−1.19 to −0.10]	[−0.88 to 0.25]	[−0.86 to 0.24]	[−0.60 to 0.54]	[−0.49 to 0.65]		[n.e.]	[−0.66 to 0.84]	[n.e.]	
							τ^2^ = n.e.	τ^2^ = 0.065	τ^2^ = n.e.	
							k = 1	k = 3	k = 1	
Problem solving therapy	**−0.74**	**−0.41**	**−0.41**	−0.12	−0.02	−0.11		−0.02	0.04	-
	[−0.97 to −0.50]	[−0.63 to −0.18]	[−0.64 to −0.18]	[−0.36 to 0.13]	[−0.35 to 0.32]	[−0.68 to 0.49]		[−0.50 to 0.36]	[−2.53 to 2.51]	
								τ^2^ = 0.044	τ^2^ = 0.156	
								k = 4	k = 2	
Cognitive-behavioural therapy	**−0.78**	**−0.45**	**−0.45**	−0.16	−0.06	−0.15	−0.04		0.12	−0.05
	[−0.91 to −0.64]	[−0.58 to −0.32]	[−0.61 to −0.28]	[−0.33 to 0.01]	[−0.33 to 0.22]	[−0.67 to 0.41]	[−0.25 to 0.17]		[−0.11 to 0.36]	[−0.31 to 0.20]
									τ^2^ = 0.030	τ^2^ = 0.031
									k = 14	k = 6
Behavioural activation	**−0.80**	**−0.47**	**−0.46**	−0.18	−0.08	−0.17	−0.06	−0.02		-
	[−1.08 to −0.51]	[−0.75 to −0.19]	[−0.76 to −0.18]	[−0.47 to 0.12]	[−0.43 to 0.26]	[−0.72 to 0.43]	[−0.38 to 0.26]	[−0.29 to 0.25]		
Interpersonal therapy	**−0.92**	**−0.59**	**−0.58**	**−0.30**	−0.19	−0.29	−0.18	−0.14	−0.12	
	[−1.14 to −0.69]	[−0.78 to −0.39]	[−0.82 to −0.34]	[−0.54 to −0.05]	[−0.53 to 0.14]	[−0.85 to 0.31]	[−0.46 to 0.09]	[−0.33 to 0.07]	[−0.44 to 0.20]	

Dashes indicate pairs of conditions that have not been compared in a study. Negative signs in the upper triangle indicate superiority of the condition in column; negative signs in the lower triangle indicate superiority of the condition in the row. Significant effects are printed in bold.

a These confidence intervals are calculated by method of moments random effects meta-analysis.

b If only a single comparison was present no credibility intervals are presented.

n.e., not estimated.

### Network Meta-Analysis

All available within-study comparisons were then synthesized with network meta-analysis (see lower triangle in Table 3). Most relative effects of psychotherapeutic interventions were absent to small (range *d* = 0.01 to *d* = −0.30) and all but one failed to reach statistical significance (the 95% CrIs did include 0). Interpersonal therapy was significantly superior to supportive therapy (*d* = −0.30, 95% CrI [−0.54 to −0.05]). All seven psychotherapeutic interventions were more beneficial than waitlist, with effect sizes between *d* = −0.62 and *d* = −0.92. Compared to usual care, all psychotherapeutic interventions except for social skills training were more beneficial, with effect sizes between *d* = −0.29 and *d* = −0.59. Similar results were found in comparison to placebo. Heterogeneity between effect sizes was low (τ^2^ = 0.010) and suggested good interpretability of the results. There was no evidence that direct and indirect effects were inconsistent (95% CrIs of differences between direct and indirect estimates included 0, see Figure S1 in Supporting Information S1).

### Moderator Analyses

We further explored the influence of several potential moderator variables. Table 4 presents the results of network-meta-analyses stratified by different study characteristics. There was no evidence to suggest effect modification for patient population and intervention format (Δ*d* = 0.08, 95% CrI [−0.34 to 0.18]; *p* = 0.54 and Δ*d* = −0.07, 95% CrI [−0.37 to 0.22]; *p* = 0.65, respectively). Thus, psychotherapeutic interventions appeared comparably effective in different populations of depressed patients, and when provided in a face-to-face and individual setting, compared to other settings. The effectiveness of psychotherapeutic interventions was the same in studies in which patients were formally diagnosed with depression and studies in which patients were probably depressed (Δ*d* = −0.11, 95% CrI [−0.39 to 0.18]; *p* = 0.45). However, the effectiveness of psychotherapy was affected by the treatment dose. Unexpectedly, low-dose treatments were more effective in a stratified analysis (Δ*d* = 0.33, 95% CrI [−0.01 to 0.65]; *p* = 0.06). However, a linear regression analysis did not confirm this initial finding (*p* = 0.63).

**Table 4:** Influence of patient population, intervention format, study quality, and study size on the results of network meta-analysis.

Characteristics	Number of Trials	Number of Patients	τ^2^	Interpersonal Therapy	Behavioural Activation	Cognitive-Behavioural Therapy	Problem Solving Therapy	Social Skills Training	Psychodynamic Therapy	Supportive Counselling	*d*-Difference or *p*-Value
**All trials**	198	15,118	0.010	−0.92	−0.80	−0.78	−0.74	−0.62	−0.72	−0.62	-
				[−1.14 to −0.69]	[−1.08 to −0.51]	[−0.91 to −0.64]	[−0.97 to −0.50]	[−1.19 to −0.10]	[−1.02 to −0.41]	[−0.82 to −0.40]	
**Patient population**											
Regular depression	94	6,992	0.015	−0.92	−0.91	−0.82	−0.70	−0.76	−0.77	−0.70	−0.08 [−0.34 to 0.18]
				[−1.26 to −0.57]	(−1.31 to −0.51)	(−1.00 to −0.63)	(−1.05 to −0.37)	(−1.39 to −0.13)	(−1.20 to −0.33)	(−1.08 to −0.31)	
Specific population	104	8,126	0.014	−0.91	−0.68	−0.74	−0.76	−0.20	−0.68	−0.58	*p* = 0.543
				[−1.24 to −0.59]	[−1.10 to −0.25]	[−0.93 to −0.56]	[−1.12 to −0.42]	[−1.55 to 1.13]	[−1.09 to −0.27]	[−0.87 to −0.29]	
**Intervention format and setting**											
Individual and face-to-face^a^	98	7,275	0.011	−0.95	−0.89	−0.84	−0.84	−0.53	−0.75	−0.68	−0.07 [−0.37 to 0.22]
				[−1.29 to −0.60]	[−1.27 to −0.53]	[−1.10 to −0.58]	[−1.22 to −0.48]	[−1.39 to 0.25]	[−1.15 to −0.35]	[−1.04 to −0.31]	
Other^a^	97	8,450	0.014	−1.00	−0.71	−0.75	−0.79	−0.74	−0.92	−0.61	*p* = 0.650
				[−1.39 to −0.59]	[−1.29 to −0.16]	[−0.90 to −0.60]	[−1.24 to −0.36]	[−1.46 to 0.03]	[−1.55 to −0.28]	[−0.91 to −0.30]	
**Treatment dose**											
Low	47	3,669	0.014	−1.12	−1.09	−1.06	−1.00	−0.49	−0.73	−1.22	0.33 [−0.01 to 0.65]
				[−1.77 to −0.47]	[−1.89 to −0.26]	[−1.36 to −0.76]	[−1.43 to −0.57]	[−1.52 to 0.54]	[−1.43 to −0.03]	[−1.77 to −0.66]	
High	151	11,449	0.010	−0.80	−0.73	−0.70	−0.80	−0.72	−0.70	−0.52	*p* = 0.059
				[−1.06 to −0.55]	[−1.04 to −0.43]	[−0.84 to −0.55]	[−1.11 to −0.48]	[−1.38 to −0.09]	[−1.02 to −0.37]	[−0.76 to −0.29]	
**Diagnosis**											
Formal diagnosis	71	5,140	0.012	−0.78	−1.03	−0.86	−1.14	−0.27	−0.87	−0.69	−0.11 [−0.39 to 0.18]
				[−1.14 to −0.45]	[−1.46 to −0.58]	[−1.10 to −0.62]	[−1.65 to −0.65]	[−62.08 to 62.10]	[−1.28 to −0.45]	[−1.10 to −0.27]	
Probable depression	127	9,978	0.011	−1.14	−0.69	−0.75	−0.64	−0.59	−0.63	−0.68	*p* = 0.454
				[−1.49 to −0.78]	[−1.09 to −0.27]	[−0.91 to −0.59]	[−0.92 to −0.34]	[−1.22 to −0.04]	[−1.08 to −0.19]	[−0.95 to −0.43]	
**Concealment of allocation**											
Adequate	34	4,621	0.018	−0.58	−0.92	−0.65	−0.50	0.03	−0.62	−0.49	0.19 [−0.08 to 0.47]
				[−1.97 to 0.77]	[−1.59 to −0.23]	[−0.89 to −0.41]	[−0.88 to −0.12]	[−62.07 to 62.74]	[−1.26 to 0.00]	[−1.03 to 0.04]	
Inadequate or not reported	164	10,497	0.011	−0.92	−0.79	−0.83	−1.02	−0.67	−0.75	−0.69	*p* = 0.162
				[−1.18 to −0.67]	[−1.11 to −0.49]	[−1.00 to −0.68]	[−1.37 to −0.67]	[−1.23 to −0.14]	[−1.12 to −0.43]	[−0.94 to −0.45]	
**Outcome assessment**											
Adequate	169	13,913	0.011	−0.90	−0.73	−0.74	−0.69	−0.62	−0.68	−0.59	0.38 [−0.06 to 0.87]
				[−1.16 to −0.62]	[−1.08 to −0.36]	[−0.88 to −0.61]	[−0.94 to −0.44]	[−1.20 to −0.04]	[−0.99 to −0.38]	[−0.82 to −0.37]	
Inadequate or not reported	29	1,205	0.024	−1.15	−1.16	−1.11	−1.08	−0.85	−0.92	−0.75	*p* = 0.100
				[−1.73 to −0.59]	[−1.76 to −0.56]	[−1.56 to −0.66]	[−2.15 to −0.04]	[−2.73 to 1.05]	[−1.96 to 0.06]	[−1.58 to 0.16]	
**Adequate analysis [ITT]**											
Yes	91	10,007	0.014	−0.89	−0.73	−0.73	−0.64	0.23	−0.72	−0.51	0.13 [−0.14 to 0.39]
				[−1.19 to −0.58]	[−1.19 to −0.26]	[−0.90 to −0.55]	[−0.93 to −0.36]	[−61.92 to 61.92]	[−1.12 to −0.32]	[−0.79 to −0.22]	
No	107	5,111	0.012	−0.88	−0.85	−0.83	−1.07	−0.65	−0.68	−0.77	*p* = 0.354
				[−1.29 to −0.46]	[−1.22 to −0.49]	[−1.05 to −0.63]	[−1.63 to −0.50]	[−1.18 to −0.09]	[−1.14 to −0.21]	[−1.13 to −0.39]	
**Trial size**											
moderate to large [≥25]	95	11,704	0.013	−0.85	−0.69	−0.68	−0.57	−0.51	−0.60	−0.51	0.29 [−0.01 to 0.58]
				[−1.12 to −0.58]	[−1.22 to −0.16]	[−0.84 to −0.53]	[−0.85 to −0.29]	[−62.04 to 61.30]	[−0.96 to −0.24]	[−0.78 to −0.26]	
small [< 25]	103	3,414	0.013	−0.99	−0.92	−0.95	−1.23	−0.72	−0.90	−0.79	*p* = 0.063
				[−1.48 to −0.49]	[−1.27 to −0.56]	[−1.19 to −0.71]	[−1.76 to −0.71]	[−1.29 to −0.14]	[−1.38 to −0.37]	[−1.17 to −0.39]	
**Trial size**											
large [≥50]	36	7,229	0.025	−0.73	−0.14	−0.57	−0.46	−0.25	−0.12	−0.29	0.33 [0.08 to 0.61]
				[−1.14 to −0.32]	[−62.11 to 62.05]	[−0.80 to −0.35]	[−0.81 to −0.12]	[−62.44 to 61.74]	[−0.92 to 0.67]	[−0.67 to 0.09]	
small to moderate [< 50]	162	7,889	0.009	−1.00	−0.92	−0.90	−1.09	−0.72	−0.89	−0.80	*p* = 0.012
				[−1.28 to −0.70]	[−1.23 to −0.60]	[−1.07 to −0.73]	[−1.52 to −0.67]	[−1.31 to −0.15]	[−1.23 to −0.55]	[−1.05 to −0.53]	
**Publication year**											
Early [< 2000]	115	3,686	0.012	−0.79	−0.86	−0.83	−1.16	−0.63	−0.61	−0.81	0.14 [−0.15 to 0.42]
				[−1.23 to −0.34]	[−1.26 to −0.46]	[−1.06 to −0.59]	[−1.73 to −0.65]	[−1.22 to −0.07]	[−1.09 to −0.15]	[−1.20 to −0.40]	
Recent [≥2000]	83	11,432	0.013	−0.95	−0.74	−0.73	−0.61	0.10	−0.76	−0.51	*p* = 0.363
				[−1.22 to −0.67]	[−1.17 to −0.29]	[−0.89 to −0.57]	[−0.89 to −0.33]	[−61.51 to 62.60]	[−1.14 to −0.39]	[−0.77 to −0.25]	

Negative effect sizes indicate superiority of the specific intervention against waitlist.

a Two trials contributed to both strata, one trial did not contribute.

Of the three study quality indicators, one was related to treatment effects in trend. Treatment effects were smaller in studies where outcome was assessed with self-report measures or blinded observers, compared to non-blind observers (Δ*d* = 0.38, 95% CrI [−0.06 to 0.87]; *p* = 0.10). Also, there was a hint towards smaller effects in studies with adequately concealed randomisation sequences, compared to studies with inadequate or unclear concealment (Δ*d* = 0.19, 95% CrI [−0.08 to 0.47]; *p* = 0.16). No significant difference was found between studies in which analysis was to intention-to-treat, compared to studies with more complete analysis (Δ*d* = 0.13, 95% CrI [−0.14 to 0.39]; *p* = 0.35).

Treatment effects were lower in at least moderately sized studies (Δ*d* = 0.29, 95% CrI [−0.01 to 0.58]; *p* = 0.06) and large studies (Δ*d* = 0.33, 95% CrI [0.08 to 0.61]; *p* = 0.01). Consistent with this result, we found a significant funnel plot asymmetry (Egger's test *p*<0.001; see Figure S2 in Supporting Information S1), suggesting a linear relation between the standard error as a proxy for study size and effect size (i.e., small studies showed larger effects). Because sample size is discussed as a proxy for study quality, we also investigated associations between the two variables. We found that all three aspects of study quality were significantly more often fulfilled in larger studies (all *p*-values <0.001, see Table S3 in Supporting Information S1). No clear difference between study effects was found when we contrasted studies published before 2000 and studies published in 2000 or after (Δ*d* = 0.14, 95% CrI [−0.15 to 0.42]; *p* = 0.36).

### Stepwise Restriction of Network Meta-Analyses According to Sample Size

**Figure 2:**
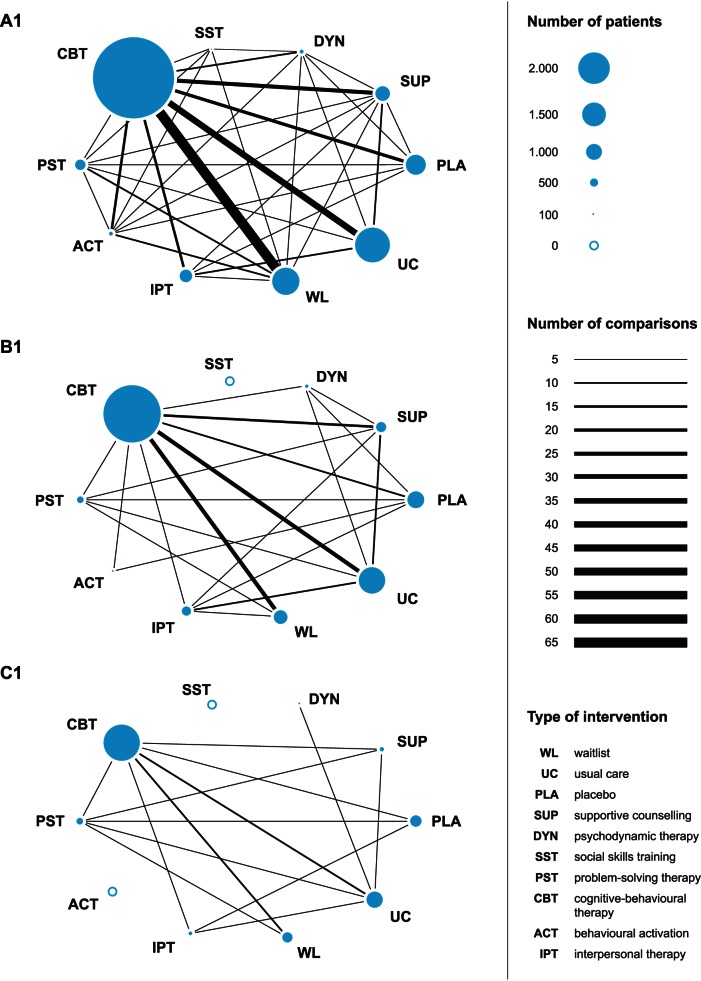
Networks of evidence of all trials (A1), moderately sized trials (B1), and large trials (C1). ACT, behavioural activation; CBT, cognitive-behavioural therapy; DYN, psychodynamic therapy; ES, *d* effect size; IPT, interpersonal therapy; PLA, placebo; PST, problem solving therapy; SST, social skills training; SUP, supportive counselling; UC, usual care; WL, waitlist.

**Figure 3:**
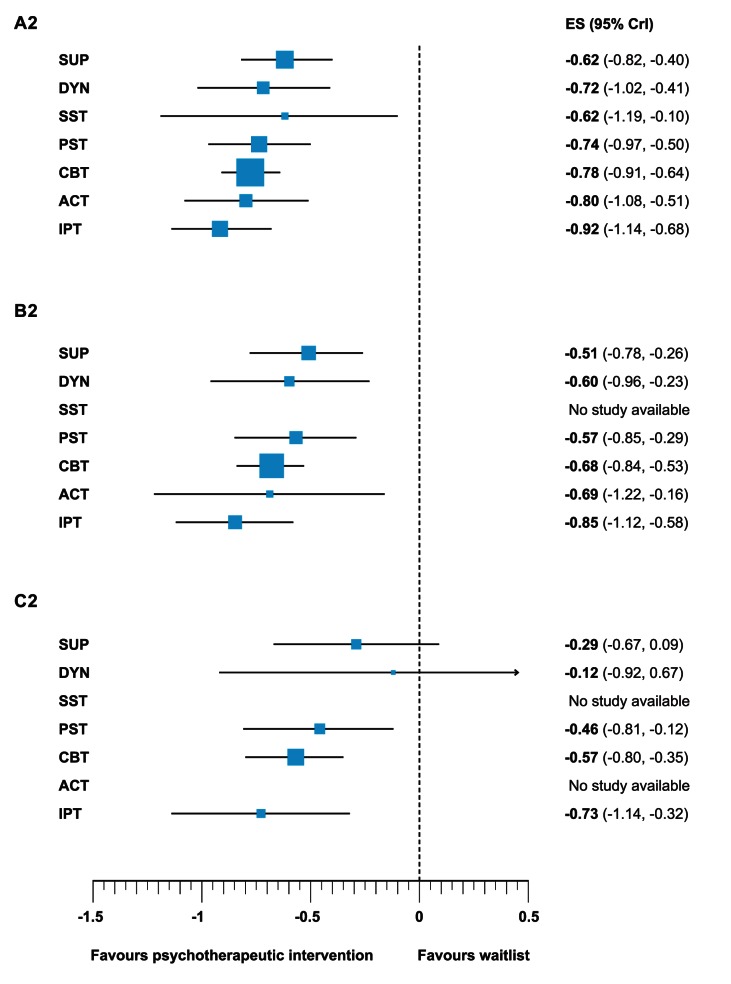
Efficacy of different psychotherapeutic intervention compared to waitlist of all trials (A2), moderately sized trials (B2), and large trials (C2). ACT, behavioural activation; CBT, cognitive-behavioural therapy; DYN, psychodynamic therapy; IPT, interpersonal therapy; PST, problem solving therapy; SST, social skills training; SUP, supportive counselling.

The moderator analyses strongly suggested that the studies included in the present meta-analysis are prone to bias. In order to reduce overestimation of effects and test the robustness of the findings, we conducted further analyses restricted to studies with moderate sample size and studies with large sample size. Since there was an association between sample size and study quality, restricting the analysis to larger studies might reduce bias owing to low study quality. Figures 2 and 3 present networks of evidence and forest plots for all (A1 and A2, cf. lower triangle in Table 2), at least moderately sized (B1 and B2), and large (C1 and C2) trials.

The networks of evidence (A1, B1, and C1) reflect the available within-study comparisons for each contrast between conditions and the number of patients investigated for each condition (Figure 2). Restricting analysis to studies with moderate and large sample sizes reduced the number of psychotherapeutic interventions that could be adequately represented in the network. In the network of studies with at least moderate sample size, no studies were available for social skills training, and only two studies were available for behavioural activation. In the network based on large studies, no studies were available for behavioural activation and social skills training, and only one study was available for psychodynamic therapy. Thus, the influence of sample size on estimated treatment efficacy could not be determined for social skills training, and the evidence base for behavioural activation and psychodynamic therapy was limited.

The forest plots in Figure 3 (A2, B2, and C2) show the relative effect of psychotherapeutic interventions compared with waitlist. In the network meta-analysis restricted to at least moderately sized trials, significant moderate to large effects against waitlist were found for supportive counselling, psychodynamic therapy, problem solving therapy, cognitive behavioural therapy, behavioural activation and interpersonal therapy (all 95% CrIs did not include 0). The effects of all these psychotherapeutic interventions were reduced in comparison to the network meta-analysis based on all studies (A2; range of differences between estimates for treatments Δ*d* = 0.07 to Δ*d* = 0.17). The only significant difference between treatments was that interpersonal therapy appeared to be more effective than supportive counselling (Δ*d* = −0.34, 95% CrI [−0.63 to −0.03]). As mentioned, the effect estimate for behavioural activation was based only on two studies and thus should be interpreted with caution. All relative effects found in the network of at least moderately sized studies are shown in the upper triangle of Table S4 in Supporting Information S1.

In the network meta-analysis restricted to large studies problem solving therapy, cognitive behavioural therapy, and interpersonal therapy showed moderate effects compared to waitlist (95% CrIs did not include 0). Supportive counselling and psychodynamic therapy were not significantly more effective than waitlist (95% CrIs included 0). The effects of all treatments in the network were again reduced, compared to the network of at least moderately sized studies (range of differences between estimates for treatments Δ*d* = 0.11 to Δ*d* = 0.48). The effect size difference between interpersonal therapy and supportive counselling was no longer significant in this analysis (Δ*d* = −0.45, 95% CrI [−0.89 to 0.02]). As mentioned above, the effect of psychodynamic therapy is based on only one study and thus should be interpreted with caution. All relative effects found in the network large studies are shown in the lower triangle in Table S4 in Supporting Information S1.

Heterogeneity between effect sizes in the network meta-analyses restricted to ≥25 and ≥50 patients per condition was low (τ^2^ = 0.013 and τ^2^ = 0.025, respectively) and suggested good interpretability of the results. Again in neither network meta-analyses did we find evidence for inconsistency between direct and indirect estimates (95% CrIs of differences between direct and indirect estimates included 0, see Figures S3 and S4 in Supporting Information S1).

## Discussion

After synthesizing 198 randomized controlled trials, we found evidence that most of the seven psychotherapeutic interventions under investigation have comparable effects on depressive symptoms and achieve moderate to large effects vis-à-vis waitlist. The only significant difference was that interpersonal therapy was somewhat more beneficial than supportive counselling. All seven psychotherapeutic interventions achieved a small to moderate effect compared to usual care. These effects were statistically significant for six psychotherapeutic interventions, and insignificant for social skills training.

Moderator analyses suggested that psychotherapeutic interventions work similarly well in different populations of depressed patients and in different settings. We found evidence that study quality influences treatment effects. Studies using non-blind outcome assessors found significantly larger effects than studies that used self-report measures or blinded observers. Also, there was a trend towards larger effects in studies with inadequately concealed randomisation sequence compared to studies with adequate concealment of allocation. This network meta-analysis also found a decrease in treatment effects in studies with a larger sample size. Treatment effects were about a moderate effect size lower in studies that had 50 or more patients per condition.

To adjust for small study effects, we conducted additional analyses restricted to at least moderately sized and large studies. Adjusting for small study effects resulted in divergent conclusions about the robustness of different psychotherapeutic approaches. Although somewhat reduced in magnitude, problem solving therapy, cognitive behavioural therapy, and interpersonal therapy showed significant moderate effects vis-à-vis waitlist in all restricted analyses. For supportive counselling and psychodynamic therapy, significant effects against waitlist were found in the network of at least moderately sized trials, but not in the network of large trials. Behavioural activation was more effective than waitlist in the network of at least moderately sized studies; however no information from large studies was available. Social skills training was only investigated in small studies.

The diminished effects found for psychodynamic therapy and supportive therapy in the network of large studies need further discussion. Only one large study was available for psychodynamic therapy, in which five sessions of psychodynamic therapy were compared to usual care in patients with chronic depression [41]. From a clinical perspective, a 3-fold treatment dose might be the lower limit for such patients. This was shown in the study on the cognitive behavioural-analysis system of psychotherapy (CBASP), which uses about 18 sessions [42]. The evidence base for supportive counselling was broader and our results suggest that supportive counselling might be a less adequate treatment for depression. However, there have been criticisms of the implementation of supportive counselling in psychotherapy outcome research. Conceptual restrictions might limit its effectiveness (i.e., in many cases supportive counselling was not intended to be therapeutic [43],[44]). After controlling for researcher allegiance, a recent meta-analysis found no difference between supportive counselling and other treatments [45]. Taking these limitations into account, we believe that dismissing psychodynamic therapy and supportive therapy as suboptimal treatments for depression is unjustified.

This research has some limitations. Like standard meta-analysis, network meta-analysis assumes the included studies are drawn from the same population (i.e., homogeneity). But network meta-analysis makes an additional assumption to come to consistent results (i.e., no inconsistency of estimates) from direct and indirect estimates of relative effects. Yet, both assumptions hold for our results because heterogeneity was low in all our analyses, and we found no evidence of inconsistency between direct and indirect estimates.

Network meta-analysis also assumes that particular treatments are similar in rationale and procedure (i.e., the specific ingredients responsible for change), allowing us to group them together as one knot in the network according to a classification system. Grouping treatments that have important differences in rationale and procedures might obscure differences between treatments and cause us to underestimate the relative efficacy of intervention strategies. For the present study, an established classification system of psychotherapeutic interventions for depression [19] differentiated seven clearly defined treatment strategies and did not include a category summarising “other psychotherapeutic interventions.” But the category of usual care may have merged treatments of different intensity, which might have returned biased results. Nor did our study control for researcher allegiance bias [46]–[48], which may have introduced bias in effect estimates. Because data on a comparison level like allegiance cannot be considered in network meta-analysis, it is likely that researcher preferences influence the treatment effects found in this study to some extent.

Our approach has some additional minor limitations that should be taken into account when interpreting our findings. Clinical ratings lead to larger effects than self-report measures [49]. Our study summarizes both types of outcomes in a single aggregate measure in order to make all trials available for the network meta-analysis, and this might overestimate the treatment effects. Our results might have limited generalizability, because studies were mostly conducted in Western countries. It is not possible to come to conclusions about long-term effects because our effectiveness data were collected at the end of treatment. Finally, interaction effects and corresponding *p*-values were derived from a model that assumes the same interaction effect across comparisons. A more flexible modelling approach would allow for different interaction effects across comparisons, but data was too scarce to allow this.

Given the availability of effective treatment options and the severity of the disorder, the use of wait list controls should be considered as unethical. It should only be used in trials, where no adequate treatment is available. In the future, large trials that compare psychotherapeutic approaches with robust evidence of effectiveness may also prove the effectiveness of supportive counselling, psychodynamic therapy, and behavioural activation. To control for allegiance bias, we suggest future trials be carried out by collaborative research teams, representing allegiances to each intervention. For the dissemination of study results into practice, the availability of treatments in the health care system should also guide selection of psychotherapeutic approaches for clinical studies.

### Conclusions

Small study effects affect the results of randomized controlled trials of psychotherapeutic interventions and should receive more attention in further meta-analyses. In larger trials, we found robust effects for cognitive-behavioural therapy, interpersonal therapy, and problem-solving therapy, while effects were less robust for psychodynamic therapy, supportive counselling, and behavioural activation. However, effect differences between these six psychotherapeutic interventions were rather small. Overall, we found that different psychotherapeutic interventions for depression have comparable, moderate-to-large effects.

## Supporting Information

Supporting Information S1 **Supporting information to the manuscript.**
Contents:Text S1. WinBUGs model for main analysis.Text S2. Bibliography of 198 studies included in the meta-analysis.Text S3. PRISMA checklist.Table S1. Bibliography of 198 studies included in the meta-analysis.Table S2. Description of individual studies (methodological characteristics and intervention).Table S3. Aspects of study quality in small to moderate to and large studies (column percentages).Table S4. Relative effect sizes (and 95% credibility intervals) of psychotherapeutic interventions and control conditions from network meta-analyses restricted to at least moderately sized (upper triangle) and large (lower triangle) studies.Figure S1. Forest plot of inconsistency in closed loops with 95% confidence interval.Figure S2. Funnel plot of studies comparing psychotherapeutic interventions with waitlist to including prediction lines from meta-regression models with the standard error as an explanatory variable and 5% contour areas to display areas of significance and non-significance.Figure S3. Forest plot of inconsistency in closed loops with 95% confidence interval (moderately sized studies).Figure S4. Forest plot of inconsistency in closed loops with 95% confidence interval (large studies)
(DOCX)

## Abbreviations

ACT: behavioural activation; CBASP, cognitive behavioural-analysis system of psychotherapy; CBT, cognitive-behavioural therapy; CI, confidence interval; CrI, credibility interval; d, effect size; D, Somer's D; DYN, psychodynamic therapy; ES, *d* effect size; IPT, interpersonal therapy; k, number of comparisons; M, mean; p, p-value; PLA, placebo; PST, problem solving therapy; SD, standard deviation; SST, social skills training; SUP, supportive counselling; UC, usual care; WL, waitlist; T^2^, tau square
