# Supplementary material for: Comparative Efficacy of Seven Psychotherapeutic Interventions for Patients with Depression: A Network Meta-Analysis
Source: PLoS Med. 2013 May 28;10(5):e1001454. doi: 10.1371/journal.pmed.1001454 (PMC3665892; doi:10.1371/journal.pmed.1001454)
Supplement: Supporting Information S1 — Supporting information to the manuscript. Contents:Text S1. WinBUGs model for main analysis.Text S2. Bibliography of 198 studies included in the meta-analysis.Text S3. PRISMA checklist.Table S1. Bibliography of 198 studies included in the meta-analysis.Table S2. Description of individual studies (methodological characteristics and intervention).Table S3. Aspects of study quality in small to moderate to and large studies (column percentages).Table S4. Relative effect sizes (and 95% credibility intervals) of psychotherapeutic interventions and control conditions from network meta-analyses restricted to at least moderately sized (upper triangle) and large (lower triangle) studies.Figure S1. Forest plot of inconsistency in closed loops with 95% confidence interval.Figure S2. Funnel plot of studies comparing psychotherapeutic interventions with waitlist to including prediction lines from meta-regression models with the standard error as an explanatory variable and 5% contour areas to display areas of significance and non-significance.Figure S3. Forest plot of inconsistency in closed loops with 95% confidence interval (moderately sized studies).Figure S4. Forest plot of inconsistency in closed loops with 95% confidence interval (large studies) (DOCX) [file pmed.1001454.s001.docx]

Supporting information to the manuscript

“Comparative efficacy of seven psychotherapeutic interventions for patients with depression:
a network meta-analysis”

Revised manuscript submitted to *PlosMedicine*

Contents

Text S1 2

Text S2 3

Text S3 18

Table S1 21

Table S2 34

Table S3 45

Table S4 46

Figure S1 48

Figure S2 49

Figure S3 50

Figure S4 51

# Text S1

WinBUGs model for main analysis

ns: number of trials, nt: number of treatments, na[i]: number of arms in i^th^ trial, seff[i,j]: effect size comparing j^th^ treatment to baseline treatment in i^th^ trial, se[i,j]: standard error of effect size comparing j^th^ treatment to baseline treatment in i^th^ trial, t[i,k]: treatment in k^th^ arm of i^th^ trial.

model {

for(i in 1:ns){

w[i,1] <- 0

delta[i,t[i,1]] <- 0

# Vague priors for trial baselines

mu[i] ~ dnorm(0,0.001)

for (k in 1:na[i]){

prec[i,t[i,k]] <- 1/pow(se[i,t[i,k]],2)

# Likelihood function

seff[i,t[i,k]] ~ dnorm(intermediate[i,k],prec[i,t[i,k]])

# Evidence synthesis model

intermediate[i,k] <- mu[i] + delta[i,t[i,k]]

}

for (k in 2:na[i]){

# Trial-specific effect size

delta[i,t[i,k]] ~ dnorm(md[i,t[i,k]],taud[i,t[i,k]])

# Mean effect size

md[i,t[i,k]] <- d[t[i,k]] - d[t[i,1]] + sw[i,k]

# Precision of mean effect size distribution

taud[i,t[i,k]] <- tau*2*(k-1)/k

# Adjustments for multi-arm trials

w[i,k] <- delta[i,t[i,k]] - d[t[i,k]] + d[t[i,1]]

sw[i,k] <- sum(w[i,1:k-1]) /(k-1)

}

}

# Define reference treatment

d[1] <- 0

for (k in 2:nt){

# Vague priors for basic parameters

d[k] ~ dnorm(0,0.001)

}

# Vague priors for variance of random effects distribution

tau2 ~ dgamma(0.01,0.01)

orig.tau <- 1/tau2

sd <- pow(tau2, -0.5)

}

# Text S2

Bibliography of 198 studies included in the meta-analysis

Alexopoulos, G. S., Raue, P., & Areán, P. (2003). Problem-solving therapy versus supportive therapy in geriatric major depression with executive dysfunction. *American Journal of Geriatric Psychiatry, 11*(1), 46-52. doi: 10.1001/jama.2011.713

Allart-van Dam, E., Hosman, C. M., Hoogduin, C. A., & Schaap, C. P. (2003). The coping with depression course: Short-term outcomes and mediating effects of a randomized controlled trial in the treatment of subclinical depression. *Behavior Therapy 34*(3), 381-396. doi: 10.1016/S0005-7894(03)80007-2

Andersson, G., Bergstrom, J., Hollandare, F., Carlbring, P., Kaldo, V., & Ekselius, L. (2005). Internet-based self-help for depression: Randomised controlled trial. *British Journal of Psychiatry, 187*, 456-461. doi: 10.1192/bjp.187.5.456

Areán, P. A., Perri, M., Nezu, A., Schein, R., Christopher, F., & Joseph, T. (1993). Comparative effectiveness of social problem-solving therapy and reminiscence therapy as treatments for depression in older adults. *Journal of Consulting and Clinical Psychology, 61*(6), 1003-1010. doi: 10.1037/0022-006X.61.6.1003

Areán, P. A., Raue, P., Mackin, R. S., Kanellopoulos, D., McCulloch, C., & Alexopoulos, G. S. (2010). Problem-solving therapy and supportive therapy in older adults with major depression and executive dysfunction. *American Journal of Psychiatry, 167*(11), 1391-1398. doi: 10.1176/appi.ajp.2010.09091327

Ayen, I., & Hautzinger, M. (2004). Cognitive behavior therapy for depression in menopausal women. A controlled, randomized treatment study [Kognitive Verhaltenstherapie bei Depressionen im Klimakterium: Eine kontrollierte, randomisierte Interventionsstudie]. *Zeitschrift für Klinische Psychologie und Psychotherapie, 33*(4), 290-299. doi: 10.1026/1616-3443.33.4.290

Baker, A. L., Kavanagh, D. J., Kay-Lambkin, F. J., Hunt, S. A., Lewin, T. J., Carr, V. J., & Connolly, J. (2009). Randomized controlled trial of cognitive–behavioural therapy for coexisting depression and alcohol problems: Short term outcome. *Addiction, 105*(1), 87-99. doi: 10.1111/j.1360-0443.2009.02757.x

Barnhofer, T., Crane, C., Hargus, E., Amarasinghe, M., Winder, R., & Williams, J. M. (2009). Mindfulness-based cognitive therapy as a treatment for chronic depression: A preliminary study. *Behaviour Research and Therapy, 47*(5), 366-373. doi: 10.1016/j.brat.2009.01.019

Barrera, M. (1979). An evaluation of a brief group therapy for depression. *Journal of Consulting and Clinical Psychology, 47*(2), 413-415. doi: 10.1037/0022-006X.47.2.413

Barrett, J. E., Williams, J. W., Oxman, T. E., Frank, E., Katon, W., Sullivan, M., . . . Sengupta, A. S. (2001). Treatment of dysthymia and minor depression in primary care: A randomized trial in patients aged 18 to 59 years. *Journal of Family Practice, 50*(5), 405-412.

Beach, S., & O'Leary, K. (1992). Treating depression in the context of marital discord: Outcome and predictors of response of marital therapy versus cognitive therapy. *Behavior Therapy, 23*(4), 507-528. doi: 10.1016/S0005-7894(05)80219-9

Beeber, L. S., Holditch-Davis, D., Perreira, K., Schwartz, T. A., Lewis, V., Blanchard, H., . . . Davis Goldman, B. (2010). Short-term in-home intervention reduces depressive symptoms in early head start latina mothers of infants and toddlers. *Research in Nursing and Health, 33*(1), 60-76. doi: 10.1002/nur.20363

Bellack, A. S., Herse, M., & Himmelhoch, J. (1981). Social skills training compared with pharmacotherapy and psychotherapy in the treatment of unipolar depression. *American Journal of Psychiatry, 138*(12), 1562-1567.

Berger, T., Hämmerli, K., Gubser, N., Andersson, G., & Caspar, F. (2011). Internet-based treatment of depression: A randomized controlled trial comparing guided with unguided self-help. *Cognitive Behaviour Therapy, 40*(4), 251-266. doi: 10.1080/16506073.2011.616531

Beutler, L. E., Engle, D., Mohr, D., Daldrup, R. J., Bergan, J., Meredith, K., & Merry, W. (1991). Predictors of differential response to cognitive, experiential, and self-directed psychotherapeutic procedures. *Journal of Consulting and Clinical Psychology, 59*(2), 333-340. doi: 10.1037/0022-006X.59.2.333

Bodenmann, G., Plancherel, B., Widmer, K., Meuwly, N., Beach, S. R., Gabriel, B., . . . Schramm, E. (2008). Effects of coping-oriented couples therapy on depression: A randomized clinical trial. *Journal of Consulting and Clinical Psychology, 76*(6), 944-954. doi: 10.1037/a0013467

Bolton, P., Bass, J., Neugebauer, R., Verdeli, H., Clougherty, K. F., Wickramaratne, P., . . . Weissman, M. M. (2003). Group interpersonal psychotherapy for depression in rural Uganda: A randomized controlled trial. *Journal of the American Medical Association, 289*(23), 3117-3124.

Bowman, D., Scogin, F., & Lyrene, B. (1995). The efficacy of self-examination therapy and cognitive bibliotherapy in the treatment of mild to moderate depression. *Psychotherapy Research, 5*(2), 131-140.

Bright, J., Baker, K., & Neimeyer, R. (1999). Professional and paraprofessional group treatments for depression: A comparison of cognitive-behavioral and mutual support interventions. *Journal of Consulting and Clinical Psychology, 67*(4), 491-501. doi: 10.1037/0022-006X.67.4.491

Brown, R. A., & Lewinsohn, P. M. (1984). A psychoeducational approach to the treatment of depression: Comparison of group, individual, and minimal contact procedures. *Journal of Consulting and Clinical Psychology, 52*(5), 774-783.

Carpenter, K. M., Smith, J. L., Aharonovich, E., & Nunes, E. V. (2008). Developing therapies for depression in drug dependence: Results of a stage 1 therapy study. *American Journal of Drug and Alcohol Abuse, 34*(5), 642-652. doi: 10.1080/00952990802308171

Carrington, C. H. (1979). *A comparison of cognitive and analytically oriented brief treatment approaches to depression in black women.* Dissertation, University of Maryland, Maryland.

Castonguay, L. G., Schut, A. J., Aikens, D. E., Constantino, M. J., Laurenceau, J., Bologh, L., & Burns, D. D. (2004). Integrative cognitive therapy for depression: A preliminary investigation. *Journal of Psychotherapy Integration, 14*(1), 4-20.

Chen, C., Tseng, Y., Chou, F., & Wang, S. (2000). Effects of support group intervention in postnatally distressed women: A controlled study in Taiwan. *Journal of Psychosomatic Research, 49*(6), 395-399. doi: 10.1016/S0022-3999(00)00180-X

Cho, H. J., Kwon, J. H., & Lee, J. J. (2008). Antenatal cognitive-behavioral therapy for prevention of postpartum depression: A pilot study. *Yonsei Medical Journal*(4), 553-562. doi: 10.3349/ymj.2008.49.4.553

Choi, I., Zou, J., Titov, N., Dear, B. F., Li, S., Johnston, L., . . . Hunt, C. (2012). Culturally attuned internet treatment for depression amongst Chinese Australians: A randomised controlled trial. Journal of Affective Disorders, 136(3), 459-468. doi: 10.1016/j.jad.2011.11.003

Cramer, H., Salisbury, C., Conrad, J., Eldred, J., & Araya, R. (2011). Group cognitive behavioural therapy for women with depression: Pilot and feasibility study for a randomised controlled trial using mixed methods. *BMC Psychiatry, 11*, 82. doi: 10.1186/1471-244X-11-82

Christensen, H., Griffiths, K. M., & Jorm, A. F. (2004). Delivering interventions for depression by using the internet: Randomised controlled trial. *British Medical Journal, 328*(7434), 265-268. doi: 10.1136/bmj.37945.566632.EE

Collins, R. W. (1996). *The treatment of depression: An integrative psychotherapy model.* Dissertation, Saybrook Institute, San Francisco.

Comas-Diaz, L. (1981). Effects of cognitive and behavioral group treatment on the depressive symptomatology of Puerto Rican women. *Journal of Consulting and Clinical Psychology, 49*(5), 627-632. doi: 10.1037/0022-006X.49.5.627

Conoley, C. W., & Garber, R. A. (1985). Effects of reframing and self-control directives on loneliness, depression, and controllability. *Journal of Counseling Psychology 32*(1), 139-142. doi: 10.1037/0022-0167.32.1.139

Conradi, H. J., de Jonge, P., Kluiter, H., Smit, A., van der Meer, K., Jenner, J. A., . . . Ormel, J. (2007). Enhanced treatment for depression in primary care: Long-term outcomes of a psycho-educational prevention program alone and enriched with psychiatric consultation or cognitive behavioral therapy. *Psychological Medicine, 37*(6), 849-862. doi: 10.1017/S0033291706009809

Cooper, P. J., Murray, L., Wilson, A., & Romaniuk, H. (2003). Controlled trial of the short- and long-term effect of psychological treatment of post-partum depression: 1. impact on maternal mood. *British Journal of Psychiatry, 182*, 412-419. doi: 10.1192/bjp.02.177

Covi, L., & Lipman, R. S. (1987). Cognitive behavioral group psychotherapy combined with imipramine in major depression. *Psychopharmacology Bulletin, 23*(1), 173-176.

Cullen, J. M. (2003). *Testing the effectiveness of behavioral activation therapy in the treatment of acute unipolar depression.* Dissertation, Western Michigan University, Kalamazoo.

Dekker, R. L., Moser, D. K., Peden, A. R., & Lennie, T. A. (2011). Cognitive therapy improves three-month outcomes in hospitalized patients with heart failure. *Journal of Cardiac Failure, 18*(1), 10-20. doi: 10.1016/j.cardfail.2011.09.008

DeRubeis, R., Hollon, S., Amsterdam, J., Shelton, R., Young, P., Salomon, R., . . . Gallop, R. (2005). Cognitive therapy vs. medications in the treatment of moderate to severe depression. *Archives of General Psychiatry, 62*(4), 409-416. doi: 10.1001/archpsyc.62.4.409

Dimidjian, S., Hollon, S. D., Dobson, K. S., Schmaling, K. B., Kohlenberg, R. J., Addis, M. E., . . . Jacobson, N. S. (2006). Randomized trial of behavioral activation, cognitive therapy, and antidepressant medication in the acute treatment of adults with major depression. *Journal of Consulting and Clinical Psychology, 74*(4), 658-670. doi: 10.1037/0022-006X.74.4.658

Dobkin, R. D., Menza, M., Allen, L. A., Gara, M. A., Mark, M. H., Tiu, J., . . . Friedman, J. (2011). Cognitive-behavioral therapy for depression in Parkinson's disease: A randomized, controlled trial. [Article]. *American Journal of Psychiatry, 168*(10), 1066-1074. doi: 10.1176/appi.ajp.2011.10111669

Dowrick, C., Dunn, G., Ayuso-Mateos, J. L., Dalgard, O. S., Page, H., Lehtinen, V., . . . Wilkinson, G. (2000). Problem solving treatment and group psychoeducation for depression: Multicentre randomised controlled trial. *British Medical Journal, 321*(7274), 1450-1454. doi: 10.1136/bmj.321.7274.1450

Duarte, P. S., Miyazaki, M. C., Blay, S. L., & Sesso, R. (2009). Cognitive-behavioral group therapy is an effective treatment for major depression in hemodialysis patients. *Kidney International, 76*(4), 414-421. doi: 10.1038/ki.2009.156

Duchac, N. (2003). *The use of a rational emotive behavioral therapy to treat depression with incarcerated males.* Dissertation, University of Toledo, Toledo.

Dwight-Johnson, M., Aisenberg, E., Golinelli, D., Hong, S., O'Brien, M., & Ludman, E. (2011). Telephone-based cognitive-behavioral therapy for Latino patients living in rural areas: A randomized pilot study. *Psychiatric Services, 62*(8), 936-942. doi: 10.1176/appi.ps.62.8.936

Ekers, D., Richards, D., McMillan, D., Bland, J. M., & Gilbody, S. (2011). Behavioural activation delivered by the non-specialist: Phase II randomised controlled trial. *British Journal of Psychiatry, 198*(1), 66-72. doi: 10.1192/bjp.bp.110.079111

Elkin, I., Shea, M., Watkins, J. T., Imber, S. D., & et al. (1989). National Institute of Mental Health Treatment of Depression Collaborative Research Program: General effectiveness of treatments. *Archives of General Psychiatry, 46*(11), 971-982.

Epstein, D. (1987). *Aerobic activity versus group cognitive therapy: An evaluative study of contrasting interventions for the alleviation of clinical depression.* Dissertation, University of Nevada, Reno.

Evans, R., & Connis, R. (1995). Comparison of brief group therapies for depressed cancer patients receiving radiation treatment. *Public Health Reports, 110*(3), 306-311.

Faramarzi, M., Alipor, A., Esmaelzadeh, S., Kheirkhah, F., Poladi, K., & Pash, H. (2008). Treatment of depression and anxiety in infertile women: Cognitive behavioral therapy versus fluoxetine. *Journal of Affective Disorders, 108*(1-2), 159-164. doi: 10.1016/j.jad.2007.09.002

Fleming, B. M., & Thornton, D. W. (1980). Coping skills training as a component in the short-term treatment of depression. *Journal of Consulting and Clinical Psychology, 48*(5), 652-654. doi: 10.1037/0022-006X.48.5.652

Floyd, M., Scogin, F., McKendree-Smith, N. L., Floyd, D. L., & Rokke, P. D. (2004). Cognitive therapy for depression: A comparison of individual psychotherapy and bibliotherapy for depressed older adults. *Behavior Modification, 28*(2), 297-318. doi: 10.1177/0145445503259284

Forsyth, K. M. (2001). *The design and implementation of a depression prevention program.* Dissertation, University of Rhode Island, Kingston.

Foster, R. P. (2007). Treating depression in vulnerable urban women: A feasibility study of clinical outcomes in community service settings. *American Journal of Orthopsychiatry, 77*(3), 443-453. doi: 10.1037/0002-9432.77.3.443

Freedland, K. E., Skala, J. A., Carney, R. M., Rubin, E. H., Lustman, P. J., Davila-Roman, V. G., . . . Hogue, C. W. (2009). Treatment of depression after coronary artery bypass surgery: A randomized controlled trial. *Archives of General Psychiatry, 66*(4), 387-396. doi: 10.1001/archgenpsychiatry.2009.7

Frothingham, S. S. (2006). *The effects of an optimism-based cognitive behavioral intervention on mood and functioning in cardiac patients.* Dissertation, University of Southern Mississippi, Hattiesburg.

Fry, P. (1984). Cognitive training and cognitive-behavioral variables in the treatment of depression in the elderly. *Clinical Gerontologist 3*(1), 25-45. doi: 10.1300/J018v03n01_04

Fuchs, C. Z., & Rehm, L. P. (1977). A self-control behavior therapy program for depression. *Journal of Consulting and Clinical Psychology, 45*(2), 206-215. doi: 10.1037/0022-006X.45.2.206

Furukawa, T. A., Horikoshi, M., Kawakami, N., Kadota, M., Sasaki, M., Sekiya, Y., . . . Terashima, H. (2012). Telephone cognitive-behavioral therapy for subthreshold depression and presenteeism in workplace: A randomized controlled trial. *PloS one, 7*(4), e35330. doi: 10.1371/journal.pone.0035330

Gallagher-Thompson, D., & Steffen, A. (1994). Comparative effects of cognitive-behavioral and brief psychodynamic psychotherapies for depressed family caregivers. *Journal of Consulting and Clinical Psychology, 62*(3), 543-549. doi: 10.1037/0022-006X.62.3.543

Gallagher, D. E. (1981). Behavioral group therapy with elderly depressives: An experimental study. In D. Upper & S. Ross (Eds.), *Behavioral group therapy* (pp. 187-224). Champaign: Research Press.

Gallagher, D. E., & Thompson, L. W. (1982). Treatment of major depressive disorder in older adult outpatients with brief psychotherapies. *Psychotherapy: Theory, Research and Practice, 19*(4), 482-490. doi: 10.1037/h0088461

Gardner, P., & Oei, T. P. (1981). Depression and self-esteem: An investigation that used behavioral and cognitive approaches to the treatment of clinically depressed clients. *Journal of Clinical Psychology, 37*(1), 128-135.

Grote, N. K., Swartz, H. A., Geibel, S. L., Zuckoff, A., Houck, P. R., & Frank, E. (2009). A randomized controlled trial of culturally relevant, brief interpersonal psychotherapy for perinatal depression. Psychiatric Services, 60(3), 313-321. doi: 10.1176/appi.ps.60.3.313

Hamamci, Z. (2006). Integrating psychodrama and cognitive behavioral therapy to treat moderate depression. *Arts in Psychotherapy, 33*(3), 199-207. doi: 10.1016/j.aip.2006.02.001

Hamdan-Mansour, A. M., Puskar, K., & Bandak, A. G. (2009). Effectiveness of cognitive-behavioral therapy on depressive symptomatology, stress and coping strategies among Jordanian university students. *Issues in Mental Health Nursing, 30*(3), 188-196. doi: doi:10.1080/01612840802694577

Haringsma, R., Engels, G. I., Cuijpers, P., & Spinhoven, P. (2006). Effectiveness of the coping with depression (CWD) course for older adults provided by the community-based mental health care system in the Netherlands: A randomized controlled field trial. *International Psychogeriatrics, 18*(2), 307-325. doi: 10.1017/S104161020500253X

Hautzinger, M., & Welz, S. (2004). Cognitive behavioral therapy for depressed older outpatients: A controlled, randomized trial [Kognitive Verhaltenstherapie bei Depressionen im Alter. Ergebnisse einer kontrollierten Vergleichsstudie unter ambulanten Bedingungen an Depressionen mittleren Schweregrads]. *Zeitschrift fur Gerontologie und Geriatrie, 37*(6), 427-435. doi: 10.1007/s00391-004-0262-x

Hautzinger, M., & Welz, S. (2008). Kurz- und längerfristige Wirksamkeit psychologischer Interventionen bei Depressionen im Alter [Short- and long-term efficacy of psychological intervention for depression in older adults]. *Zeitschrift für Klinische Psychologie und Psychotherapie, 37*, 52-60. doi: 10.1026/1616-3443.37.1.52

Hayden, T., Perantie, D. C., Nix, B. D., Barnes, L. D., Mostello, D. J., Holcomb, W. L., . . . Hershey, T. (2012). Treating prepartum depression to improve infant developmental outcomes: A study of diabetes in pregnancy. [Article]. *Journal of Clinical Psychology in Medical Settings, 19*(3), 285-292. doi: 10.1007/s10880-011-9294-8

Hayman, P. M., & Cope, C. S. (1980). Effects of assertion training on depression. *Journal of Clinical Psychology, 36*(2), 534-543.

Hegerl, U., Hautzinger, M., Mergl, R., Kohnen, R., Schütze, M., Scheunemann, W., . . . Henkel, V. (2009). Effects of pharmacotherapy and psychotherapy in depressed primary-care patients: A randomized, controlled trial including a patients' choice arm. *International Journal of Neuropsychopharmacology, 13*, 31-44. doi: 10.1017/S1461145709000224

Holden, J., Sagovsky, R., & Cox, J. (1989). Counselling in a general practice setting: Controlled study of heath visitor intervention in treatment of postnatal depression. *British Medical Journal, 298*(6668), 223-226. doi: 10.1136/bmj.298.6668.223

Honey, K., Bennett, P., & Morgan, M. (2002). A brief psycho-educational group intervention for postnatal depression. *British Journal of Clinical Psychology, 41*(4), 405-409. doi: 10.1348/014466502760387515

Hopko, D. R., Armento, M. E. A., Robertson, S., Ryba, M. M., Carvalho, J. P., Colman, L. K., . . . McNulty, J. K. (2011). Brief behavioral activation and problem-solving therapy for depressed breast cancer patients: Randomized trial. *Journal of Consulting and Clinical Psychology, 79*(6), 834-849. doi: 10.1037/a0025450

Hunter, S. B., Watkins, K. E., Hepner, K. A., Paddock, S. M., Ewing, B. A., Osilla, K. C., & Perry, S. (2012). Treating depression and substance use: A randomized controlled trial. *Journal of Substance Abuse Treatment, 43*(2), 137-151. doi: 10.1016/j.jsat.2011.12.004

Jacobson, N. S., Dobson, K. S., Truax, P. A., Addis, M. E., Koerner, K., Gollan, J. K., . . . Prince, S. E. (1996). A component analysis of cognitive-behavioral treatment for depression. *Journal of Consulting and Clinical Psychology, 64*(2), 295-304. doi: 10.1037/0022-006X.64.2.295

Jamison, C., & Scogin, F. (1995). The outcome of cognitive bibliotherapy with depressed adults. *Journal of Consulting and Clinical Psychology, 63*(4), 644-650. doi: 10.1037/0022-006X.63.4.644

Jarrett, R. B., Schaffer, M., McIntire, D., Witt-Browder, A., Kraft, D., & Risser, R. C. (1999). Treatment of atypical depression with cognitive therapy or phenelzine: A double-blind, placebo-controlled trial. *Archives of General Psychiatry, 56*(5), 431-437.

Johansson, R., Ekbladh, S., Hebert, A., Lindström, M., Möller, S., Petitt, E., . . . Carlbring, P. (2012). Psychodynamic guided self-help for adult depression through the internet: A randomised controlled trial. *PloS one, 7*(5), e38021. doi: 10.1371/journal.pone.0038021

Johansson, R., Sjöberg, E., Sjögren, M., Johnsson, E., Carlbring, P., Andersson, T., . . . Andersson, G. (2012). Tailored vs. standardized internet-based cognitive behavior therapy for depression and comorbid symptoms: A randomized controlled trial. *PloS one, 7*(5), e36905. doi: 10.1371/journal.pone.0036905

Johnson, J. E., & Zlotnick, C. (2012). Pilot study of treatment for major depression among women prisoners with substance use disorder. *Journal of Psychiatric Research, 46*(9), 1174-1183. doi: 10.1016/j.jpsychires.2012.05.007

Joling, K. J., van Hout, H. P. J., van't Veer-Tazelaar, P. J., van der Horst, H. E., Cuijpers, P., van de Ven, P. M., & van Marwijk, H. W. J. (2011). How effective is bibliotherapy for very old adults with subthreshold depression? A randomized controlled trial. *American Journal of Geriatric Psychiatry, 19*(3), 256-265. doi: 10.1097/JGP.0b013e3181ec8859

Kay-Lambkin, F. J., Baker, A. L., Lewin, T. J., & Carr, V. J. (2009). Computer-based psychological treatment for comorbid depression and problematic alcohol and/or cannabis use: A randomized controlled trial of clinical efficacy. *Addiction, 104*(3), 378-388. doi: 10.1111/j.1360-0443.2008.02444.x

Kelly, J. A., Murphy, D. A., Bahr, G. R., Kalichman, S. C., Morgan, M. G., Stevenson, L. Y., . . . Bernstein, B. M. (1993). Outcome of cognitive-behavioral and support group brief therapies for depressed, HIV-infected persons. *American Journal of Psychiatry, 150*(11), 1679-1686.

King, M., Sibbald, B., Ward, E., Bower, P., Lloyd, M., Gabbay, M., & Byford, S. (2000). Randomised controlled trial of non-directive counselling, cognitive-behaviour therapy and usual general practitioner care in the management of depression as well as mixed anxiety and depression in primary care. *Health Technology Assessment 4*(19), 1-83.

Kiosses, D. N., Areán, P. A., Teri, L., & Alexopoulos, G. S. (2010). Home-delivered Problem Adaptation Therapy (PATH) for depressed, cognitively impaired, disabled elders: A preliminary study. *American Journal of Geriatric Psychiatry, 18*(11), 988-998. doi: 10.1097/JGP.0b013e3181d6947d

Klein, M., Greist, J., Gurman, A., Neimeyer, R., Lesser, D., Bushnell, N., & Smith, R. (1985). A comparative outcome study of group psychotherapy vs. exercise treatments for depression. *International Journal of Mental Health, 13*, 148-177.

Koszycki, D., Bisserbe, J. C., Blier, P., Bradwejn, J., & Markowitz, J. (2012). Interpersonal psychotherapy versus brief supportive therapy for depressed infertile women: First pilot randomized controlled trial. *Archives of Women's Mental Health, 15*(3), 193-201. doi: 10.1007/s00737-012-0277-z

Krampen, G. (1997). Autogenic training applied before and in addition to integrated therapy of depressive disorders [Autogenes Training vor und begleitend zur methodenübergreifenden Einzelpsychotherapie bei depressiven Störungen]. *Zeitschrift für Klinische Psychologie, Psychiatrie und Psychotherapie, 45*(2), 214-232.

Laidlaw, K., Davidson, K., Toner, H., Jackson, G., Clark, S., Law, J., . . . Cross, S. (2008). A randomised controlled trial of cognitive behaviour therapy vs. treatment as usual in the treatment of mild to moderate late life depression. *International Journal of Geriatric Psychiatry, 23*(8), 843-850. doi: 10.1002/gps.1993

Lamers, F., Jonkers, C. C., Bosma, H., Kempen, G. I., Meijer, J. A., Penninx, B. W., . . . van Eijk, J. T. (2010). A minimal psychological intervention in chronically ill elderly patients with depression: A randomized trial. *Psychotherapy and Psychosomatics, 79*(4), 217-226. doi: 10.1159/000313690

Landreville, P., & Bissonnette, L. (1997). Effects of cognitive bibliotherapy for depressed older adults with a disability. *Clinical Gerontologist, 17*(4), 35-55. doi: 10.1300/J018v17n04_05

LaPointe, K. A., & Rimm, D. C. (1980). Cognitive, assertive, and insight-oriented group therapies in the treatment of reactive depression in women. *Psychotherapy: Theory, Research and Practice, 17*(3), 312-321. doi: 10.1037/h0085928

Lexis, M. A. S., Jansen, N. W. H., Huibers, M. J. H., van Amelsvoort, L. G. P. M., Berkouwer, A., Ton, G. T. A., . . . Kant, I. J. (2011). Prevention of long-term sickness absence and major depression in high-risk employees: A randomised controlled trial. *Occupational and Environmental Medicine, 68*(6), 400-407. doi: 10.1136/oem.2010.057877

Lincoln, N., & Flannaghan, T. (2003). Cognitive behavioral psychotherapy for depression following stroke: A randomized controlled trial. *Stroke, 34*(1), 111-115. doi: 10.1161/​01.STR.0000044167.44670.55

Lopez, D., Cuevas, P., Gomez, A., & Mendoza, J. (2004). Transference-focused psychotherapy for borderline personality disorder. A study with female patients [Psicoterapia focalizada en la transferencia para el trastorno límite de la personalidad. Un estudio con pacientes femeninas]. *Salud Mental, 27*(4), 44-54.

Lustman, P., Griffith, L., Freedland, K., Kissel, S., & Clouse, R. (1998). Cognitive behavior therapy for depression in type 2 diabetes mellitus. A randomized, controlled trial. *Annals of Internal Medicine, 129*(8), 613-621.

Lynch, D. J., Tamburrino, M. B., & Nagel, R. (1997). Telephone counseling for patients with minor depression: Preliminary findings in a family practice setting. *Journal of Family Practice, 44*(3), 293-298.

Lynch, D. J., Tamburrino, M. B., Nagel, R., & Smith, M. K. (2004). Telephone-based treatment for family practice patients with mild depression. *Psychological Reports, 94*(3 ), 785-792. doi: 10.2466/PR0.94.3.785-792

Maina, G., Forner, F., & Bogetto, F. (2005). Randomized controlled trial comparing brief dynamic and supportive therapy with waiting list condition in minor depressive disorders. *Psychotherapy and Psychosomatics, 74*(1), 43-50. doi: 10.1159/000082026

Maldonado-Lopez, A. (1982). Behavioural therapy and depression. *Revista de Psicologica General y Aplicada, 37*, 31-56.

Malouff, J. M., Lanyon, R. I., & Schutte, N. S. (1988). Effectiveness of a brief group RET treatment for divorce-related dysphoria. *Journal of Rational-Emotive and Cognitive Behavior Therapy 6*(3), 162-171. doi: 10.1007/BF01064077

Manson, S. M., & Brenneman, D. L. (1995). Chronic disease among older American Indians: Preventing depressive symptoms and related problems of coping. In D. K. Pagett (Ed.), *Handbook on Ethnicity, Aging, and Mental Health*. Westport: Greenwood Press.

Markowitz, J. C., Kocsis, J. H., Bleiberg, K. L., Christos, P. J., & Sacks, M. (2005). A comparative trial of psychotherapy and pharmacotherapy for "pure" dysthymic patients. *Journal of Affective Disorders, 89*(1-3), 167-175. doi: 10.1016/j.jad.2005.10.001

Markowitz, J. C., Kocsis, J. H., Christos, P., Bleiberg, K., & Carlin, A. (2008). Pilot study of interpersonal psychotherapy versus supportive psychotherapy for dysthymic patients with secondary alcohol abuse or dependence. *Journal of Nervous and Mental Disease, 196*(6), 468-474. doi: 10.1097/NMD.0b013e31817738f1

Markowitz, J. C., Kocsis, J. H., Fishman, B., Spielman, L. A., Jacobsberg, L. B., Frances, A. J., . . . Perry, S. W. (1998). Treatment of depressive symptoms in human immunodeficiency virus-positive patients. *Archives of General Psychiatry, 55*(5), 452-457. doi: 10.1001/archpsyc.55.5.452

Marshall, M. B., Zuroff, D. C., McBride, C., & Bagby, R. M. (2008). Self-criticism predicts differential response to treatment for major depression. *Journal of Clinical Psychology, 64*(3), 231-244. doi: 10.1002/jclp.20438

McBride, C., Segal, Z., Kennedy, S., & Gemar, M. (2007). Changes in autobiographical memory specificity following cognitive behavior therapy and pharmacotherapy for major depression. *Psychopathology, 40*(3), 147-152. doi: 10.1159/000100003

McKendree-Smith, N. L. (1998). *Cognitive and behavioral bibliotherapy for depression: An examination of efficacy and mediators and moderators of change.* Dissertation, University of Alabama, Tuscaloosa.

McLean, P. D., & Hakstian, A. R. (1979). Clinical depression: Comparative efficacy of outpatient treatments. *Journal of Consulting and Clinical Psychology, 47*(5), 818-836. doi: 10.1037/0022-006X.47.5.818

McNamara, K., & Horan, J. J. (1986). Experimental construct validity in the evaluation of cognitive and behavioral treatments for depression. *Journal of Counseling Psychology, 33*(1), 23-30. doi: 10.1037/0022-0167.33.1.23

Meager, I., & Milgrom, J. (1996). Group treatment for postpartum depression: A pilot study. *Australian and New Zealand Journal of Psychiatry, 30*(6), 852-860. doi: 10.3109/00048679609065055

Milgrom, J., Holt, C. J., Gemmill, A. W., Ericksen, J., Leigh, B., Buist, A., & Schembri, C. (2011). Treating postnatal depressive symptoms in primary care: A randomised controlled trial of GP management, with and without adjunctive counselling. *BMC Psychiatry, 11*(1), 95. doi: 10.1186/1471-244X-11-95

Milgrom, J., Negri, L., Gemmill, A., McNeil, M., & Martin, P. (2005). A randomized controlled trial of psychological interventions for postnatal depression. *British Journal of Clinical Psychology, 44*, 529-542. doi: 10.1348/014466505X34200

Miller, L., & Weissman, M. (2002). Interpersonal psychotherapy delivered over the telephone to recurrent depressives: A pilot study. *Depression and Anxiety, 16*(3), 114-117. doi: 10.1002/da.10047

Miranda, J., Chung, J., Green, B., Krupnick, J., Siddique, J., Revicki, D., & Belin, T. (2003). Treating depression in predominantly low-income young minority women: A randomized controlled trial. *Journal of the American Medical Association, 290*(1), 57-65. doi: 10.1001/jama.290.1.57

Mitchell, P. H., Veith, R. C., Becker, K. J., Buzatis, A., Cain, K. C., Fruin, M., . . . Teri, L. (2009). Brief psychosocial-behavioral intervention with antidepressant reduces poststroke depression significantly more than usual care with antidepressant: Living Well With Stroke: randomized, controlled trial. Stroke, 40, 3073-3078. doi: 10.1161/STROKEAHA.109.549808

Mohr, D. C., Carmody, T., Erickson, L., Jin, L., & Leader, J. (2011). Telephone-administered cognitive behavioral therapy for veterans served by community-based outpatient clinics. *Journal of Consulting and Clinical Psychology, 79*(2), 261-265. doi: 10.1037/a0022395

Mohr, D. C., Hart, S. L., Julian, L., Catledge, C., Honos-Webb, L., Vella, L., & Tasch, E. T. (2005). Telephone-administered psychotherapy for depression. *Archives of General Psychiatry, 62*(9), 1007-1014. doi: 10.1001/archpsyc.62.9.1007

Mohr, D. C., Likosky, W., Bertagnolli, A., Goodkin, D. E., Van Der Wende, J., Dwyer, P., & Dick, L. P. (2000). Telephone-administered cognitive-behavioral therapy for the treatment of depressive symptoms in multiple sclerosis. *Journal of Consulting and Clinical Psychology, 68*(2), 356-361. doi: 10.1037/0022-006X.68.2.356

Morris, T. (1975). *Development and assessment of a didactic cognitive-behavioural programme for the treatment of the neurotically depressed.* Toronto East General Hospital.

Mossey, J. M., Knott, K. A., Higgins, M., & Talerico, K. (1996). Effectiveness of a psychosocial intervention, interpersonal counseling, for subdysthymic depression in medically ill elderly. *Journals of Gerontology. Series A, Biological Sciences and Medical Sciences, 51*(4), M172-M178. doi: 10.1093/gerona/51A.4.M172

Mukhtar, F. (2011). Predictors of group cognitive behaviour therapy outcomes for the treatment of depression in Malaysia. *Asian Journal of Psychiatry, 4*(2), 125-128. doi: 10.1016/j.ajp.2011.04.002

Mulcahy, R., Reay, R. E., Wilkinson, R. B., & Owen, C. (2010). A randomised control trial for the effectiveness of group interpersonal psychotherapy for postnatal depression. *Archives of Women's Mental Health, 13*(2), 125-139. doi: 10.1007/s00737-009-0101-6

Murphy, G. E., Carney, R. M., Knesevich, M. A., Wetzel, R. D., & Whitworth, P. (1995). Cognitive behavior therapy, relaxation training, and tricyclic antidepressant medication in the treatment of depression. *Psychological Reports, 77*(2), 403-420.

Murray, L., Cooper, P. J., Wilson, A., & Romaniuk, H. (2003). Controlled trial of the short- and long-term effect of psychological treatment of post-partum depression: 2. Impact on the mother-child relationship and child outcome. *British Journal of Psychiatry, 182*, 420-427. doi: 10.1192/bjp.02.178

Mynors-Wallis, L., Gath, D., Lloyd-Thomas, A., & Tomlinson, D. (1995). Randomised controlled trial comparing problem solving treatment with amitriptyline and placebo for major depression in primary care. *British Medical Journal, 310*(6977), 441-445.

Neimeyer, R. A., & Feixas, G. (1990). The role of homework and skill acquisition in the outcome of group cognitive therapy for depression. *Behavior Therapy, 21*(3), 281-292. doi: 10.1016/S0005-7894(05)80331-4

Neugebauer, R., Kline, J., Markowitz, J. C., Bleiberg, K. L., Baxi, L., Rosing, M. A., . . . Keith, J. (2006). Pilot randomized controlled trial of interpersonal counseling for subsyndromal depression following miscarriage. *Journal of Clinical Psychiatry, 67*(8), 1299-1304. doi: 10.4088/JCP.v67n0819

Nezu, A. M. (1986). Efficacy of a social problem-solving therapy approach for unipolar depression. *Journal of Consulting and Clinical Psychology, 54*(2), 196-202. doi: 10.1037/0022-006X.54.2.196

Nezu, A. M., & Perri, M. G. (1989). Social problem-solving therapy for unipolar depression: An initial dismantling investigation. *Journal of Consulting and Clinical Psychology, 57*(3), 408-413. doi: 10.1037/0022-006X.57.3.408

O'Hara, M. W., Stuart, S., Gorman, L. L., & Wenzel, A. (2000). Efficacy of interpersonal psychotherapy for postpartum depression. *Archives of General Psychiatry, 57*(11), 1039-1045. doi: 10.1001/archpsyc.57.11.1039

Pace, T. M., & Dixon, D. N. (1993). Changes in depressive self-schemata and depressive symptoms following cognitive therapy. *Journal of Counseling Psychology, 40*(3), 288-294. doi: 10.1037/0022-0167.40.3.288

Padfield, M. (1976). The comparative effects of two counseling approaches on the intensity of depression among rural women of low socioeconomic status. *Journal of Counseling Psychology, 23*(3), 209-214. doi: 10.1037/0022-0167.23.3.209

Pecheur, D. R., & Edwards, K. J. (1984). A comparison of secular and religious versions of cognitive therapy with depressed Christian college students. *Journal of Psychology and Theology, 12*(1), 45-54.

Peden, A. R., Hall, L. A., Rayens, M. K., & Beebe, L. L. (2000). Reducing negative thinking and depressive symptoms in college women. *Journal of Nursing Scholarship, 32*(2), 145-151. doi: 10.1111/j.1547-5069.2000.00145.x

Pibernik-Okanovic, M., Begic, D., Ajdukovic, D., Andrijasevic, N., & Metelko, Z. (2009). Psychoeducation versus treatment as usual in diabetic patients with subthreshold depression: Preliminary results of a randomized controlled trial. *Trials, 10*(78), 1-9. doi: 10.1186/1745-6215-10-78

Power, M. J., & Freeman, C. (2012). A randomized controlled trial of IPT versus CBT in primary care: With some cautionary notes about handling missing values in clinical trials. *Clinical Psychology and Psychotherapy, 19*(2), 159-169. doi: 10.1002/cpp.1781

Prendergast, J., & Austin, M. (2001). Early childhood nurse-delivered cognitive behavioural counselling for post-natal depression. *Australasian Psychiatry, 9*(3), 255-259. doi: 10.1046/j.1440-1665.2001.00330.x

Propst, L. R., Ostrom, R., Watkins, P., Dean, T., & Mashburn, D. (1992). Comparative efficacy of religious and nonreligious cognitive-behavioral therapy for the treatment of clinical depression in religious individuals. *Journal of Consulting and Clinical Psychology, 60*(1), 94-103. doi: 10.1037/0022-006X.60.1.94

Rahman, A. (2008). "Cognitive behaviour therapy for depressed Pakistani mothers": Author's Reply. [Comment/Reply]. *Lancet, 372*(9656), 2111-2112. doi: 10.1016/S0140-6736(08)61920-0

Ransom, D., Heckman, T. G., Anderson, T., Garske, J., Holroyd, K., & Basta, T. (2008). Telephone-delivered, interpersonal psychotherapy for HIV-infected rural persons with depression: A pilot trial. *Psychiatric Services 59*(8), 871-877. doi: 10.1176/appi.ps.59.8.871

Rohan, K. J., Roecklein, K. A., Tierney Lindsey, K., Johnson, L. G., Lippy, R. D., Lacy, T. J., & Barton, F. B. (2007). A randomized controlled trial of cognitive-behavioral therapy, light therapy, and their combination for seasonal affective disorder. *Journal of Consulting and Clinical Psychology, 75*(3), 489-500. doi: 10.1037/0022-006X.75.3.489

Rohen, N. A. (2003). *Analysis of efficacy and mediators of outcome in minimal-contact cognitive bibliotherapy used in the treatment of depressive symptoms.* Dissertation, University of Alabama, Tuscaloosa.

Ross, M., & Scott, M. (1985). An evaluation of the effectiveness of individual and group cognitive therapy in the treatment of depressed patients in an inner city health centre. *Journal of the Royal College of General Practitioners, 35*(274), 239-242.

Rude, S. S. (1986). Relative benefits of assertion or cognitive self-control treatment for depression as a function of proficiency in each domain. *Journal of Consulting and Clinical Psychology, 54*(3), 390-394. doi: 10.1037/0022-006X.54.3.390

Safren, S. A., O'Cleirigh, C., Tan, J. Y., Raminani, S. R., Reilly, L. C., Otto, M. W., & Mayer, K. H. (2009). A randomized controlled trial of cognitive behavioral therapy for adherence and depression (CBT-AD) in HIV-infected individuals. *Health Psychology 28*(1), 1-10. doi: 10.1037/a0012715

Savard, J., Simard, S., Giguere, I., Ivers, H., Morin, C. M., Maunsell, E., . . . Marceau, D. (2006). Randomized clinical trial on cognitive therapy for depression in women with metastatic breast cancer: Psychological and immunological effects. *Palliative and Supportive Care, 4*(3), 219-237. doi: 10.1017/S1478951506060305

Schmidt, M. M., & Miller, W. R. (1983). Amount of therapist contact and outcome in a multidimensional depression treatment program. *Acta Psychiatrica Scandinavica, 67*(5), 319-332. doi: 10.1111/j.1600-0447.1983.tb00349.x

Schmitt, S. G. (1988). *Clinical depression: A comparative outcome study of two treatment approaches.* Dissertation, Fairleigh Dickinson University, New Jersey.

Schulberg, H., Block, M., Madonia, M., Scott, C., Rodriguez, E., Imber, S., . . . Coulehan, J. (1996). Treating major depression in primary care practice: Eight-month clinical outcomes. *Archives of General Psychiatry, 53*(10), 913-919.

Scogin, F., Hamblin, D., & Beutler, L. E. (1987). Bibliotherapy for depressed older adults: A self-help alternative. *The Gerontologist 27*(3), 383-387. doi: 10.1093/geront/27.3.383

Scogin, F., Jamison, C., & Gochneaur, K. (1989). Comparative efficacy of cognitive and behavioral bibliotherapy for mildly and moderately depressed older adults. *Journal of Consulting and Clinical Psychology, 57*(3), 403-407. doi: 10.1037/0022-006X.57.3.403

Scott, A., & Freeman, C. (1992). Edinburgh primary care depression study: Treatment outcome, patient satisfaction, and cost after 16 weeks. *British Medical Journal, 304*(6831), 883-887. doi: 10.1136/bmj.304.6831.883

Scott, C., Tacchi, M. J., Jones, R., & Scott, J. (1997). Acute and one-year outcome of a randomised controlled trial of brief cognitive therapy for major depressive disorder in primary care. *British Journal of Psychiatry, 171*, 131-134. doi: 10.1192/bjp.171.2.131

Scott, M. J., & Stradling, S. G. (1990). Group cognitive therapy for depression produces clinically significant reliable change in community-based settings. *Behavioural Psychotherapy, 18*(1), 1-19. doi: 10.1017/S014134730001795X

Selmi, P. M., Klein, M. H., Greist, J. H., Sorrell, S. P., & Erdman, H. P. (1990). Computer-administered cognitive-behavioral therapy for depression. *American Journal of Psychiatry, 147*(1), 51-56.

Serfaty, M. A., Haworth, D., Blanchard, M., Buszewicz, M., Murad, S., & King, M. (2009). Clinical effectiveness of individual cognitive behavioral therapy for depressed older people in primary care: A randomized controlled trial. *Archives of General Psychiatry, 66*(12), 1332-1340. doi: 10.1001/archgenpsychiatry.2009.165

Shaw, B. F. (1977). Comparison of cognitive therapy and behavior therapy in the treatment of depression. *Journal of Consulting and Clinical Psychology 45*(4), 543-551. doi: 10.1037/0022-006X.45.4.543

Sheeber, L. B., Seeley, J. R., Feil, E. G., Davis, B., Sorensen, E., Kosty, D. B., & Lewinsohn, P. M. (2012). Development and pilot evaluation of an Internet-facilitated cognitive-behavioral intervention for maternal depression. *Journal of Consulting and Clinical Psychology, 80*(5), 739-749. doi: 10.1037/a0028820

Simpson, S., Corney, R., & Beecham, J. (2003). A randomized controlled trial to evaluate the effectiveness and cost-effectiveness of psychodynamic counselling for general practice patients with chronic depression. *Psychological Medicine, 33*(2), 229-239. doi: 10.1017/S0033291702006517

Simson, U., Nawarotzky, U., Friese, G., Porck, W., Schottenfeld-Naor, Y., Hahn, S., . . . Kruse, J. (2008). Psychotherapy intervention to reduce depressive symptoms in patients with diabetic foot syndrome. *Diabetic Medicine, 25*(2), 206-212. doi: 10.1111/j.1464-5491.2007.02370.x

Skinner, D. A. (1983). *Self-control of depression: A comparison of behavior therapy and cognitive behavior therapy.* Dissertation, United States International University.

Sloane, R. B., Staples, F. R., & Schneider, L. S. (1985). Interpersonal therapy vs. nortriptyline for depression in the elderly. *Clinical and Pharmacological Studies in Psychiatric Disorders*, 344-346.

Snarski, M., Scogin, F., DiNapoli, E., Presnell, A., McAlpine, J., & Marcinak, J. (2011). The effects of behavioral activation therapy with inpatient geriatric psychiatry patients. *Behavior Therapy, 42*(1), 100-108. doi: 10.1016/j.beth.2010.05.001

Spek, V., Nyklicek, I., Cuijpers, P., Riper, H., Keyzer, J., & Pop, V. (2007). Internet-based cognitive behavioural therapy for subthreshold depression in people over 50 years old: A randomized controlled clinical trial *Psychological Medicine, 37*(12), 1797-1806. doi: 10.1017/S0033291707000542

Spinelli, M., & Endicott, J. (2003). Controlled clinical trial of interpersonal psychotherapy versus parenting education program for depressed pregnant women. *American Journal of Psychiatry, 160*(3), 555-562. doi: 10.1176/appi.ajp.160.3.555

Sudweeks, C. (1996). *Effects of cognitive group hypnotherapy in the alteration of depressogenic schemas.* Dissertation, Washington State University, Pullman.

Swartz, H. A., Frank, E., Zuckoff, A., Cyranowski, J. M., Houck, P. R., Cheng, Y., . . . Shear, M. K. (2008). Brief interpersonal psychotherapy for depressed mothers whose children are receiving psychiatric treatment. *American Journal of Psychiatry, 165*(9), 1155-1162. doi: 10.1176/appi.ajp.2008.07081339

Talbot, N. L., Chaudron, L. H., Ward, E. A., Duberstein, P. R., Conwell, Y., O’Hara, M. W., . . . Stuart, S. (2011). A randomized effectiveness trial of interpersonal psychotherapy for depressed women with sexual abuse histories. *Psychiatric Services, 62*(4), 374-380. doi: 10.1176/appi.ps.62.4.374

Taylor, C. B., Conrad, A., Wilhelm, F. H., Strachowski, D., Khaylis, A., Neri, E., . . . Spiegel, D. (2009). Does improving mood in depressed patients alter factors that may affect cardiovascular disease risk? *Journal of Psychiatric Research, 43*(16), 1246-1252. doi: 10.1016/j.jpsychires.2009.05.006

Taylor, F. G., & Marshall, W. L. (1977). Experimental analysis of a cognitive-behavioral therapy for depression. *Cognitive Therapy and Research, 1*(1), 59-72. doi: 10.1007/bf01173505

Teasdale, J. D., Fennell, M. J., Hibbert, G. A., & Amies, P. L. (1984). Cognitive therapy for major depressive disorder in primary care. *British Journal of Psychiatry, 144*, 400-406. doi: 10.1192/bjp.144.4.400

Teichman, Y., Bar-el, Z., Shor, H., Sirota, P., & Elizur, A. (1995). A comparison of two modalities of cognitive therapy (individual and marital) in treating depression. *Psychiatry, 58*(2), 136-148.

Teri, L., Logsdon, R., Uomoto, J., & McCurry, S. (1997). Behavioral treatment of depression in dementia patients: A controlled clinical trial. *Journals of Gerontology. Series B, Psychological Sciences and Social Sciences, 52*(4), 159-166. doi: 10.1093/geronb/52B.4.P159

Thompson, L. W., & Gallagher, D. (1984). Efficacy of psychotherapy in the treatment of late-life depression. *Advances in Behaviour Research and Therapy, 6*(2), 127-139. doi: 10.1016/0146-6402(84)90007-9

Thompson, L. W., Gallagher, D., & Breckenridge, J. S. (1987). Comparative effectiveness of psychotherapies for depressed elders. *Journal of Consulting and Clinical Psychology, 55*(3), 385-390. doi: 10.1037/0022-006X.55.3.385

Thompson, N. J., Walker, E. R., Obolensky, N., Winning, A., Barmon, C., DiIorio, C., & Compton, M. T. (2010). Distance delivery of mindfulness-based cognitive therapy for depression: Project UPLIFT. *Epilepsy and Behavior, 19*(3), 247-254. doi: 10.1016/j.yebeh.2010.07.031

Titov, N., Andrews, G., Davies, M., McIntyre, K., Robinson, E., & Solley, K. (2010). Internet treatment for depression: A randomized controlled trial comparing clinician vs. technician assistance. *PloS one, 5*(6), e10939.

Turner, R. W., Ward, M. F., & Turner, D. J. (1979). Behavioral treatment for depression: An evaluation of therapeutic components. *Journal of Clinical Psychology, 35*(1), 166-175. doi: 10.1002/1097-4679(197901)35:1<166::aid-jclp2270350127>3.0.co;2-1

Usaf, S. O., & Kavanagh, D. J. (1990). Mechanisms of improvement in treatment for depression: Test of a self-efficacy and performance model. *Journal of Cognitive Psychotherapy, 4*(1), 51-70.

van Bastelaar, K. M. P., Pouwer, F., Cuijpers, P., Riper, H., & Snoek, F. J. (2011). Web-based depression treatment for type 1 and type 2 diabetic patients: A randomized, controlled trial. *Diabetes Care, 34*(2), 320-325. doi: 10.2337/dc10-1248

van Schaik, A., van Marwijk, H., Ader, H., van Dyck, R., de Haan, M., Penninx, B., . . . Beekman, A. (2006). Interpersonal psychotherapy for elderly patients in primary care. *American Journal of Geriatric Psychiatry, 14*(9), 777-786. doi: 10.1097/01.JGP.0000199341.25431.4b

Verduyn, C., Barrowclough, C., Roberts, J., Tarrier, T., & Harrington, R. (2003). Maternal depression and child behaviour problems: Randomised placebo-controlled trial of a cognitive-behavioural group intervention. *British Journal of Psychiatry, 183*, 342-348. doi: 10.1192/02-294

Vernmark, K., Lenndin, J., Bjärehed, J., Carlsson, M., Karlsson, J., Öberg, J., . . . Andersson, G. (2010). Internet administered guided self-help versus individualized e-mail therapy: A randomized trial of two versions of CBT for major depression. *Behaviour Research and Therapy, 48*(5), 368-376. doi: 10.1016/j.brat.2010.01.005

Vitriol, V. G., Ballesteros, S. T., Florenzano, R. U., Weil, K. P., & Benadof, D. F. (2009). Evaluation of an outpatient intervention for women with severe depression and a history of childhood trauma. *Psychiatric Services, 60*(7), 936-942. doi: 10.1176/appi.ps.60.7.936

Warmerdam, L., van Straten, A., Twisk, J., Riper, H., & Cuijpers, P. (2008). Internet-based treatment for adults with depressive symptoms: Randomized controlled trial. *Journal of Medical Internet Research, 10*(4), e44. doi: 10.2196/jmir.1094

Watkins, E. R., Baeyens, C. B., & Read, R. (2009). Concreteness training reduces dysphoria: Proof-of-principle for repeated cognitive bias modification in depression. *Journal of Abnormal Psychology, 118*(1), 55-64. doi: 10.1037/a0013642

Weissman, M. M., Prusoff, B. A., Dimascio, A., Neu, C., Goklaney, M., & Klerman, G. L. (1979). The efficacy of drugs and psychotherapy in the treatment of acute depressive episodes. *American Journal of Psychiatry 136*(4), 555-558.

Wickberg, B., & Hwang, C. P. (1996). Counselling of postnatal depression: A controlled study on a population based Swedish sample. *Journal of Affective Disorders, 39*(3), 209-216. doi: 10.1016/0165-0327(96)00034-1

Wiklund, I., Mohlkert, P., & Edman, G. (2010). Evaluation of a brief cognitive intervention in patients with signs of postnatal depression: A randomized controlled trial. *Acta Obstetricia et Gynecologica Scandinavica, 89*(8), 1100-1104. doi: 10.3109/00016349.2010.500369

Williams, J. W., Barrett, J. E., Oxman, T. E., Frank, E., Katon, W., Sullivan, M., . . . Sengupta, A. S. (2000). Treatment of dysthymia and minor depression in primary care: A randomized controlled trial in older adults. *Journal of the American Medical Association, 284*(12), 1519-1526. doi: 10.1001/jama.284.12.1519

Wilson, G. L. (1990). Psychotherapy with depressed incarcerated felons: A comparative evaluation of treatments. *Psychological Reports, 67*(3 ), 1027-1041. doi: 10.2466/PR0.67.7.1027-1041

Wilson, P. H., Goldin, J. C., & Charbonneau-Powis, M. (1983). Comparative efficacy of behavioral and cognitive treatments of depression. *Cognitive Therapy and Research, 7*(2), 111-124. doi: 10.1007/BF01190064

Wollersheim, J. P., & Wilson, G. L. (1991). Group treatment of unipolar depression: A comparison of coping, supportive, bibliotherapy, and delayed treatment groups. *Professional Psychology: Research and Practice, 22*(6), 496-502. doi: 10.1037/0735-7028.22.6.496

Wong, D. F. (2008a). Cognitive and health-related outcomes of group cognitive behavioural treatment for people with depressive symptoms in Hong Kong: Randomized wait-list control study. *Australian and New Zealand Journal of Psychiatry, 42*(8), 702-711. doi: 10.1080/00048670802203418

Wong, D. F. (2008b). Cognitive behavioral treatment groups for people with chronic depression in Hong Kong: A randomized wait-list control design. *Depression and Anxiety, 25*(2), 142-148. doi: 10.1002/da.20286

Wright, J. H., Wright, A. S., Albano, A. M., Basco, M. R., Goldsmith, L. J., Raffield, T., & Otto, M. W. (2005). Computer-assisted cognitive therapy for depression: Maintaining efficacy while reducing therapist time. *American Journal of Psychiatry, 162*(6), 1158-1164. doi: 10.1176/appi.ajp.162.6.1158

Zeiss, A. M., Lewinsohn, P. M., & Muñoz, R. F. (1979). Nonspecific improvement effects in depression using interpersonal skills training, pleasant activity schedules, or cognitive training. *Journal of Consulting and Clinical Psychology, 47*(3), 427-439. doi: 10.1037/0022-006X.47.3.427

# Text S3

PRISMA Checklist: Comparative efficacy of seven psychotherapeutic interventions for patients with depression: a network meta-analysis

| **Section/topic** | **Item No** | **Checklist item** | **Reported in Paragraph** |
| --- | --- | --- | --- |
| **Title** |  |  |  |
| Title | 1 | Identify the report as a systematic review, meta-analysis, or both | Title page |
| Abstract |  |  |  |
| Structured summary | 2 | Provide a structured summary including, as applicable, background, objectives, data sources, study eligibility criteria, participants, interventions, study appraisal and synthesis methods, results, limitations, conclusions and implications of key findings, systematic review registration number | Title page |
| **Introduction** | |  |  |
| Rationale | 3 | Describe the rationale for the review in the context of what is already known | Introduction: Paragraphs 1-6 |
| Objectives | 4 | Provide an explicit statement of questions being addressed with reference to participants, interventions, comparisons, outcomes, and study design (PICOS) | Introduction:  Paragraph 7 |
| **Methods** |  |  |  |
| Protocol and registration | 5 | Indicate if a review protocol exists, if and where it can be accessed (such as web address), and, if available, provide registration information including registration number | Introduction:  Paragraph 7 |
| Eligibility criteria | 6 | Specify study characteristics (such as PICOS, length of follow-up) and report characteristics (such as years considered, language, publication status) used as criteria for eligibility, giving rationale | Method:  Paragraph 2 |
| Information sources | 7 | Describe all information sources (such as databases with dates of coverage, contact with study authors to identify additional studies) in the search and date last searched | Method:  Paragraph 1 |
| Search | 8 | Present full electronic search strategy for at least one database, including any limits used, such that it could be repeated | Method:  Paragraph 1 |
| Study selection | 9 | State the process for selecting studies (that is, screening, eligibility, included in systematic review, and, if applicable, included in the meta-analysis) | Result:  Paragraph 1 |
| Data collection process | 10 | Describe method of data extraction from reports (such as piloted forms, independently, in duplicate) and any processes for obtaining and confirming data from investigators | Method:  Paragraph 4 |
| Data items | 11 | List and define all variables for which data were sought (such as PICOS, funding sources) and any assumptions and simplifications made | Method:  Paragraphs 3-6 |
| Risk of bias in individual studies | 12 | Describe methods used for assessing risk of bias of individual studies (including specification of whether this was done at the study or outcome level), and how this information is to be used in any data synthesis | Method:  Paragraph 5 |
| Summary measures | 13 | State the principal summary measures (such as risk ratio, difference in means). | Method:  Paragraph 7 |
| Synthesis of results | 14 | Describe the methods of handling data and combining results of studies, if done, including measures of consistency (such as I2 statistic) for each meta-analysis | Method:  Paragraphs  8-10, 12 |
| Risk of bias across studies | 15 | Specify any assessment of risk of bias that may affect the cumulative evidence (such as publication bias, selective reporting within studies) | Method:  Paragraph 11 |
| Additional analyses | 16 | Describe methods of additional analyses (such as sensitivity or subgroup analyses, meta-regression), if done, indicating which were pre-specified | Method:  Paragraph 11 |
| **Results** |  |  |  |
| Study selection | 17 | Give numbers of studies screened, assessed for eligibility, and included in the review, with reasons for exclusions at each stage, ideally with a flow diagram | Results:  Paragraph 1 |
| Study characteristics | 18 | For each study, present characteristics for which data were extracted (such as study size, PICOS, follow-up period) and provide the citations | Supplementary material |
| Risk of bias within studies | 19 | Present data on risk of bias of each study and, if available, any outcome-level assessment (see item 12). | Supplementary material |
| Results of individual studies | 20 | For all outcomes considered (benefits or harms), present for each study (a) simple summary data for each intervention group and (b) effect estimates and confidence intervals, ideally with a forest plot | Results:  Paragraph  2-3  Table 3 |
| Synthesis of results | 21 | Present results of each meta-analysis done, including confidence intervals and measures of consistency | Results:  Paragraphs  2-3 |
| Risk of bias across studies | 22 | Present results of any assessment of risk of bias across studies (see item 15) | Results:  Paragraphs  4-6 |
| Additional analysis | 23 | Give results of additional analyses, if done (such as sensitivity or subgroup analyses, meta-regression) (see item 16) | Results:  Paragraphs  7-11 |
| **Discussion** | |  |  |
| Summary of evidence | 24 | Summarise the main findings including the strength of evidence for each main outcome; consider their relevance to key groups (such as health care providers, users, and policy makers) | Discussion:  Paragraphs  1-4 |
| Limitations | 25 | Discuss limitations at study and outcome level (such as risk of bias), and at review level (such as incomplete retrieval of identified research, reporting bias) | Discussion:  Paragraphs  5-8 |
| Conclusions | 26 | Provide a general interpretation of the results in the context of other evidence, and implications for future research | Discussion:  Paragraph 9 |
| **Funding** |  |  |  |
| Funding | 27 | Describe sources of funding for the systematic review and other support (such as supply of data) and role of funders for the systematic review | Title page |

# Table S1

Description of individual studies (patient characteristics and outcome)

| First author | Year | Total number of patients | Number of patients per group | Effect Size and CI | Outcome measure | Outcome assessment | Diagnosis | Patient population |
| --- | --- | --- | --- | --- | --- | --- | --- | --- |
| Alexopoulos | 2003 | 25 | SUP=13, PST=12 | PST vs. SUP: -1.02 (-1.83 to -0.20) | HAMD | Adequate | Formal diagnosis | Specific |
| Allart | 2003 | 102 | UC=41, CBT=61 | CBT vs. UC: -0.57 (-0.97 to -0.17) | BDI | Adequate | Probable depression | Regular |
| Andersson | 2005 | 85 | PLA=49, CBT=36 | CBT vs. PLA: -0.87 (-1.32 to -0.43) | BDI, MADRS-S | Adequate | Probable depression | Regular |
| Arean | 1993 | 38 | PST=28  WL=10 | PST vs. WL: -1.25 (-2.02 to -0.49) | BDI, GDS, HAMD | Adequate | Formal diagnosis | Specific |
| Arean | 2010 | 221 | SUP=111, PST=110 | PST vs. SUP: -0.38 (-0.65 to -0.12) | HAMD | Adequate | Formal diagnosis | Specific |
| Ayen | 2004 | 41 | SUP=20, CBT=11  WL=10 | SUP vs. WL: -2.00 (-2.91 to -1.10)  CBT vs. WL: -3.69 (-5.12 to -2.25) | BDI | Adequate | Probable depression | Specific |
| Baker | 2009 | 141 | WL=70, CBT=71 | WL vs. CBT: -0.09 (-0.41 to 0.24) | BDI-II | Adequate | Probable depression | Specific |
| Barnhofer | 2009 | 31 | UC=15, CBT=16 | CBT vs. UC: -0.89 (-1.16 to -0.17) | BDI | Adequate | Formal diagnosis | Regular |
| Barrera | 1979 | 20 | ACT=10  WL=10 | ACT vs. WL: 0.00 (-0.84 to 0.84) | BDI | Adequate | Probable depression | Regular |
| Barrett | 2001 | 161 | PLA=81, PST=80 | PST vs. PLA: 0.07 (-0.24 to 0.38) | HSCL-20 | Adequate | Probable depression | Regular |
| Beach | 1992 | 30 | WL=15, CBT=15 | CBT vs. WL: -1.34 (-2.12 to -0.56) | BDI | Adequate | Probable depression | Regular |
| Beeber | 2010 | 71 | UC=37, IPT=34 | IPT vs. UC: -0.77 (-1.24 to -0.29) | CES-D | Adequate | Probable depression | Specific |
| Bellack | 1981 | 37 | DYN=17, SST=20 | SST vs. DYN: 0.00 (-0.63 to 0.63) | BDI, HAMD | Adequate | Probable depression | Regular |
| Berger | 2011 | 51 | CBT=25  WL=26 | CBT vs. WL: -1.11 (-1.70 to -0.53) | BDI-II | Adequate | Probable depression | Regular |
| Beutler | 1991 | 41 | SUP=20, CBT=21 | CBT vs. SUP: -0.17 (-0.77 to 0.43) | BDI, HAMD | Inadequate or not reported | Formal diagnosis | Regular |
| Bodenmann | 2008 | 37 | CBT=19, IPT=18 | CBT vs. IPT 0.02 (-0.62 to 0.65) | BDI, HAMD | Inadequate or not reported | Probable depression | Regular |
| Bolton | 2003 | 341 | UC=178, IPT=163 | IPT vs. UC: -1.06 (-1.28 to -0.83) | HSCL | Adequate | Probable depression | Regular |
| Bowman | 1995 | 30 | WL=10, PST=10, CBT=10 | PST vs. WL: -0.91 (-1.80 to -0.02)  CBT vs. WL: -1.25 (-2.18 to -0.31) | BDI, HAMD | Inadequate or not reported | Probable depression | Regular |

Table S1

Description of individual studies (patient characteristics and outcome) (continued)

| First author | Year | Total number of patients | Number of patients per group | Effect Size and CI | Outcome measure | Outcome assessment | Diagnosis | Patient population |
| --- | --- | --- | --- | --- | --- | --- | --- | --- |
| Bright | 1999 | 67 | SUP=36, CBT=31 | CBT vs. SUP: 0.09 (-0.38 to 0.57) | BDI, HAMD | Adequate | Probable depression | Regular |
| Brown | 1984 | 63 | WL=11, CBT=52 | CBT vs. WL: -0.41 (-1.05 to 0.24) | BDI, CES-D | Adequate | Probable depression | Regular |
| Carpenter | 2008 | 38 | PLA=20, ACT=18 | ACT vs. PLA 0.34 (-0.29 to 0.97) | HAMD | Inadequate or not reported | Probable depression | Specific |
| Carrington | 1979 | 30 | WL=10, DYN=10, CBT=10 | DYN vs. WL -1.88 (-2.92 to -0.84)  CBT vs. WL: -3.58 (-5.02 to -2.14) | BDI | Adequate | Formal diagnosis | Regular |
| Castonguay | 2004 | 21 | PLA=10, CBT=11 | CBT vs. PLA -1.80 (-2.80 to -0.79) | BDI, HAMD | Adequate | Formal diagnosis | Regular |
| Chen | 2000 | 60 | UC=30, SUP=30 | SUP vs. UC: -0.50 (-1.01 to 0.00) | BDI | Adequate | Probable depression | Specific |
| Cho | 2008 | 22 | UC=12, CBT=10 | CBT vs. UC: -1.00 (-1.87 to -0.14) | BDI | Adequate | Probable depression | Specific |
| Choi | 2012 | 63 | CBT=32,  WL=31 | CBT vs. WL: -0.55 (-1.05 to -0.06) | BDI, PHQ-9, K10 | Adequate | Formal diagnosis | Specific |
| Christensen | 2004 | 360 | PLA=178, CBT=182 | CBT vs. PLA: -0.36 (-0.57 to -0.15) | CES-D | Adequate | Probable depression | Regular |
| Collins | 1996 | 51 | WL=25, CBT=26 | CBT vs. WL: 0.00 (-0.54 to 0.54) | BDI | Adequate | Formal diagnosis | Regular |
| Comas-Diaz | 1981 | 26 | WL=10, CBT=8, ACT=8 | CBT vs. WL: -1.70 (-2.77 to -0.64)  ACT vs. WL: -1.70 (-2.77 to -0.64) | BDI, HAMD | Inadequate or not reported | Probable depression | Specific |
| Conoley | 1985 | 38 | WL=19, SST=19 | SST vs. WL: -0.18 (-0.80 to 0.45) | BDI | Adequate | Probable depression | Specific |
| Conradi | 2007 | 116 | UC=72, CBT=44 | CBT vs. UC: 0.00 (-0.37 to 0.37) | BDI | Adequate | Probable depression | Regular |
| Cooper | 2003 | 193 | UC=52, SUP=48, DYN=50, CBT=43 | SUP vs. UC: -0.26 (-0.65 to 0.13)  DYN vs. UC: -0.53 (-0.92 to -0.13)  CBT vs. UC: -0.44 (-0.84 to -0.03) | EPDS | Adequate | Formal diagnosis | Specific |
| Covi | 1987 | 47 | DYN=20, CBT=27 | CBT vs. DYN: 0.00 (-0.57 to 0.57) | BDI | Adequate | Formal diagnosis | Regular |
| Cramer | 2011 | 67 | CBT=48  UC=19 | CBT vs. UC: -0.25 (-0.77 to 0.28) | PHQ-9 | Adequate | Probable depression | Regular |

Table S1

Description of individual studies (patient characteristics and outcome) (continued)

| First author | Year | Total number of patients | Number of patients per group | Effect Size and CI | | Outcome measure | Outcome assessment | Diagnosis | Patient population |
| --- | --- | --- | --- | --- | --- | --- | --- | --- | --- |
| Cullen | 2002 | 14 | WL=8, ACT=6 | ACT vs. WL: -1.82 (-3.04 to -0.59) | | BDI-II | Adequate | Formal diagnosis | Regular |
| Dekker | 2012 | 41 | CBT=20,  UC=21 | CBT vs. UC: -0.11 (-0.71 to 0.49) | | BDI-II | Adequate | Probable depression | Specific |
| DeRubeis | 2005 | 120 | PLA=60, CBT=60 | CBT vs. PLA: -0.24 (-0.60 to 0.12) | | HAMD | Adequate | Formal diagnosis | Regular |
| Dimidjian | 2006 | 116 | PLA=41, CBT=38 ACT=37 | | CBT vs. PLA: -0.28 (-0.72 to 0.16)  ACT vs. PLA: -0.24 (-0.68 to 0.20) | BDI, HAMD | Adequate | Formal diagnosis | Regular |
| Dobkin | 2011 | 80 | CBT=41  WL=39 | | CBT vs. WL: -1.13 (-1.60 to -0.66) | BDI, HAMD | Adequate | Formal diagnosis | Specific |
| Dowrick | 2000 | 425 | UC=189, PST=128, CBT=108 | | PST vs. UC: -0.25 (-0.47 to -0.02)  CBT vs. UC: -0.07 (-0.31 to 0.17) | BDI | Adequate | Probable depression | Regular |
| Duarte | 2009 | 85 | UC=44, CBT=41 | | CBT vs. UC: -0.80 (-1.24 to -0.37) | BDI | Adequate | Formal diagnosis | Specific |
| Duchac | 2002 | 32 | WL=16, CBT=16 | | CBT vs. WL: -0.08 (-0.75 to 0.60) | BDI II, HAMD | Inadequate or not reported | Probable depression | Specific |
| Dwight | 2011 | 101 | CBT=50  UC=51 | | CBT vs. UC: -1.73 (-2.18 to -1.27) | PHQ-9, HSCL | Adequate | Probable depression | Regular |
| Ekers | 2011 | 47 | ACT=23  UC=24 | | ACT vs. UC: -1.13 (-1.74 to -0.52) | BDI-II | Adequate | Formal diagnosis | Regular |
| Elkin | 1989 | 179 | PLA=59, CBT=59, IPT=61 | | CBT vs. PLA: -0.36 (-0.72 to 0.00)  IPT vs. PLA: -0.24 (-0.60 to 0.12) | BDI, HAMD | Adequate | Formal diagnosis | Regular |
| Epstein | 1986 | 19 | WL=10, CBT=9 | | CBT vs. WL: -0.09 (-0.95 to 0.77) | BDI, SDS | Adequate | Probable depression | Regular |
| Evans | 1995 | 72 | PLA=24, SUP=21, CBT=27 | | SUP vs. PLA: -0.95 (-1.56 to -0.34)  CBT vs. PLA: -0.60 (-1.15 to -0.05) | CES-D, SCLD | Adequate | Probable depression | Specific |
| Faramarzi | 2008 | 59 | UC=30, CBT=29 | | CBT vs. UC: -1.73 (-2.32 to -1.13) | BDI | Adequate | Formal diagnosis | Specific |
| Fleming | 1980 | 22 | SUP=9, CBT=13 | | CBT vs. SUP: 0.00 (-0.82 to 0.82) | BDI | Adequate | Probable depression | Regular |
| Floyd | 2004 | 28 | WL=7, CBT=21 | | CBT vs. WL: -1.14 (-2.03 to -0.25) | GDS, HAMD | Inadequate or not reported | Probable depression | Specific |
| Forsyth | 2000 | 59 | WL=28, IPT=31 | | IPT vs. WL: -1.72 (-2.31 to -1.12) | BDI | Adequate | Probable depression | Specific |

Table S1

Description of individual studies (patient characteristics and outcome) (continued)

| First author | Year | Total number of patients | Number of patients per group | Effect Size and CI | | Outcome measure | Outcome assessment | Diagnosis | Patient population |
| --- | --- | --- | --- | --- | --- | --- | --- | --- | --- |
| Foster | 2007 | 91 | SUP=45.5, CBT=45.5 | | CBT vs. SUP: 0.15 (-0.26 to 0.56) | BDI, CES-D | Adequate | Probable depression | Specific |
| Freedland | 2009 | 123 | UC=40, SUP=42, CBT=41 | | SUP vs. UC: -0.47 (-0.90 to -0.03)  CBT vs. UC: -0.88 (-1.33 to -0.43) | BDI, HAMD | Adequate | Probable depression | Specific |
| Frothingham | 2005 | 43 | UC=18, CBT=25 | | CBT vs. UC: -0.06 (-0.65 to 0.54) | CDS | Adequate | Formal diagnosis | Specific |
| Fry | 1984 | 25 | WL=10, CBT=15 | CBT vs. WL: -0.44 (-1.22 to 0.35) | | MMPI-D | Adequate | Probable depression | Specific |
| Fuchs | 1977 | 18 | WL=10, SUP=8 | SUP vs. WL: -0.38 (-1.28 to 0.51) | | BDI, MMPI-D | Adequate | Probable depression | Regular |
| Furukawa | 2012 | 118 | CBT=58,  WL=60 | CBT vs. WL: -0.68 (-1.04 to -0.31) | | BDI-II, K6 | Adequate | Probable depression | Regular |
| Gallagher | 1981 | 28 | SUP=14, ACT=14 | ACT vs. SUP: 0.00 (-0.72 to 0.72) | | BDI, MMPI-D | Adequate | Probable depression | Specific |
| Gallagher | 1982 | 30 | DYN=10, CBT=10, ACT=10 | CBT vs. DYN: -0.65 (-1.52 to 0.21)  ACT vs. DYN: -0.33 (-1.17 to 0.52) | | BDI, HAMD, SDS | Adequate | Formal diagnosis | Specific |
| Gallagher | 1994 | 52 | DYN=21, CBT=31 | CBT vs. DYN: -0.40 (-0.96 to 0.15) | | BDI, HAMD, ZUNG | Adequate | Probable depression | Specific |
| Gardner | 1981 | 16 | CBT=8, ACT=8 | CBT vs. ACT: -0.66 (-1.62 to 0.30) | | BDI, ZUNG | Adequate | Probable depression | Regular |
| Grote | 2009 | 53 | IPT=25,  UC=28 | IPT vs. UC: -1.25 (-1.84 to -0.67) | | BDI, EPDS | Adequate | Probable depression | Specific |
| Hamamci | 2006 | 27 | UC=11, CBT=16 | CBT vs. UC: -1.40 (-2.24 to 0.56) | | BDI | Adequate | Probable depression | Specific |
| Hamdan-Mansour | 2009 | 84 | WL=40, CBT=44 | CBT vs. WL: -0.63 (-1.06 to -0.19) | | BDI | Adequate | Probable depression | Specific |
| Haringsma | 2005 | 110 | WL=58, CBT=52 | CBT vs. WL: -0.46 (-0.83 to -0.08) | | CES-D, HADS-D | Adequate | Probable depression | Specific |
| Hautzinger | 2004 | 85 | WL=30, CBT=55 | CBT vs. WL: -0.93 (-1.39 to -0.47) | | GDS, IDS, SCLD | Adequate | Probable depression | Specific |
| Hautzinger | 2008 | 109 | SUP=51, CBT=58 | CBT vs. SUP: -0.40 (-0.77 to -0.02) | | GDS, IDS | Adequate | Probable depression | Specific |
| Hayden | 2012 | 34 | CBT=20,  SUP=14 | CBT vs. SUP: -0.41 (-1.08 to 0.26) | | BDI | Adequate | Formal diagnosis | Specific |
| Hayman | 1980 | 28 | WL=12, SST=16 | SST vs. WL: -0.48 (-1.21 to 0.26) | | BDI | Adequate | Probable depression | Regular |

Table S1

Description of individual studies (patient characteristics and outcome) (continued)

| First author | Year | Total number of patients | Number of patients per group | Effect Size and CI | Outcome measure | Outcome assessment | Diagnosis | Patient population |
| --- | --- | --- | --- | --- | --- | --- | --- | --- |
| Hegerl | 2009 | 120 | SUP=59, CBT=61 | CBT vs. SUP: -0.53 (-0.89 to -0.17) | HAMD, IDS | Adequate | Probable depression | Regular |
| Holden | 1989 | 50 | UC=24, SUP=26 | SUP vs. UC: -0.70 (-1.26 to -0.14) | EPDS | Adequate | Probable depression | Specific |
| Honey | 2002 | 45 | UC=22, CBT=23 | CBT vs. UC: -0.35 (-0.93 to 0.23) | EPDS | Adequate | Probable depression | Specific |
| Hopko | 2011 | 80 | ACT=42  PST=38 | ACT vs. PST: -0.15 (-0.58 to 0.29) | BDI-II, HAMD | Adequate | Formal diagnosis | Specific |
| Hunter | 2012 | 73 | CBT=47,  UC=26 | CBT vs. UC: -0.40 (-0.87 to 0.08) | BDI-II | Adequate | Probable depression | Specific |
| Jacobson | 1996 | 99 | CBT=43, ACT=56 | ACT vs. CBT: -0.14 (-0.53 to 0.26) | BDI, HAMD | Inadequate or not reported | Formal diagnosis | Regular |
| Jamison | 1995 | 72 | WL=39, CBT=33 | CBT vs. WL: -1.58 (-2.11 to -1.05) | BDI, HAMD | Inadequate or not reported | Formal diagnosis | Regular |
| Jarrett | 1999 | 72 | PLA=36, CBT=36 | CBT vs. PLA: -0.58 (-1.05 to -0.12) | BDI, HAMD | Adequate | Formal diagnosis | Regular |
| Johansson | 2012 | 92 | DYN=46,  PLA=46 | DYN vs. PLA: -0.96 (-1.39 to -0.53) | BDI-II, PHQ, MADRS | Adequate | Formal diagnosis | Regular |
| Johansson | 2012 | 79 | CBT=73,  PLA=84 | CBT vs. PLA: -0.69 (-1.01 to -0.37) | BDI-II, MADRS | Adequate | Probable depression | Regular |
| Johnson | 2012 | 38 | ACT=19,  PLA=19 | ACT vs. PLA: -0.68 (-1.32 to -0.03) | HAMD | Inadequate or not reported | Formal diagnosis | Specific |
| Joling | 2011 | 170 | CBT=86  WL=84 | CBT vs. WL: -1.03 (-1.34 to -0.71) | CES-D | Adequate | Probable depression | Specific |
| Kay-Lambkin | 2009 | 82 | PLA=27.33, CBT=54.67 | CBT vs. PLA: -0.71 (-1.18 to -0.25) | BDI | Adequate | Probable depression | Specific |
| Kelly | 1993 | 41 | SUP=14, CBT=27 | CBT vs. SUP: 0.38 (-0.26 to 1.02) | CES-D, SCLD | Adequate | Probable depression | Specific |
| King | 2000 | 197 | UC=67, SUP=67, CBT=63 | SUP vs. UC: -0.49 (-0.83 to -0.15)  CBT vs. UC: -0.34 (-0.68 to 0.01) | BDI | Adequate | Probable depression | Regular |
| Kiosses | 2010 | 30 | SUP=15, PST=15 | PST vs. SUP: -0.73 (-1.45 to -0.01) | HAMD | Inadequate or not reported | Formal diagnosis | Specific |
| Klein | 1985 | 22 | PLA=8, CBT=14 | CBT vs. PLA: 0.74 (-0.13 to 1.61) | CMID, SCL90D | Adequate | Probable depression | Regular |

Table S1

Description of individual studies (patient characteristics and outcome) (continued)

| First author | Year | Total number of patients | Number of patients per group | Effect Size and CI | | Outcome measure | Outcome assessment | Diagnosis | Patient population |
| --- | --- | --- | --- | --- | --- | --- | --- | --- | --- |
| Koszycky | 2012 | 31 | IPT=15,  SUP=16 | IPT vs. SUP: -0.61 (-1.32 to 0.09) | | BDI, MADRS | Inadequate or not reported | Formal diagnosis | Specific |
| Krampen | 1997 | 29 | WL=14, CBT=15 | CBT vs. WL: -0.72 (-1.45 to 0.01) | | BDI | Adequate | Probable depression | Regular |
| Laidlaw | 2008 | 40 | UC=20, CBT=20 | CBT vs. UC: -0.42 (-1.04 to 0.19) | | BDI | Adequate | Formal diagnosis | Specific |
| Lamers | 2010 | 262 | UC=135, CBT=127 | CBT vs. UC: -0.16 (-0.40 to 0.08) | | BDI | Adequate | Probable depression | Specific |
| Landreville | 1997 | 23 | WL=13, CBT=10 | CBT vs. WL: -0.30 (-1.10 to 0.50) | | BDI, GDS, IDD | Adequate | Probable depression | Specific |
| LaPointe | 1980 | 33 | DYN=11, SST=10, CBT=12 | SST vs. DYN: 0.00 (-0.82 to 0.82)  CBT vs. DYN: 0.00 (-0.79 to 0.79) | | BDI | Adequate | Probable depression | Regular |
| Lexis | 2011 | 139 | PST=69,  UC=70 | PST vs. UC: -0.28 (-0.61 to 0.05) | | BDI-II, HADS-D | Adequate | Probable depression | Specific |
| Lincoln | 2003 | 80 | UC=41, CBT=39 | | CBT vs. UC: 0.00 (-0.43 to 0.43) | BDI, WDI | Adequate | Probable depression | Specific |
| Lopez | 2004 | 20 | PLA=10, DYN=10 | | DYN vs. PLA: -1.81 (-2.84 to -0.78) | HAMD | Adequate | Probable depression | Regular |
| Lustman | 1998 | 42 | UC=22, CBT=20 | | CBT vs. UC -1.06 (-1.70 to -0.42) | BDI | Adequate | Formal diagnosis | Specific |
| Lynch | 1997 | 24 | UC=13, PST=11 | | PST vs. UC: -0.61 (-1.40 to 0.19) | BDI | Adequate | Probable depression | Regular |
| Lynch | 2004 | 22 | UC=13, PST=9 | | PST vs. UC: -0.09 (-0.90 to 0.73) | BDI | Adequate | Probable depression | Regular |
| Maina | 2005 | 30 | WL=10, SUP=10, DYN=10 | | SUP vs. WL: -1.15 (-2.07 to -0.23)  DYN vs. WL: -1.09 (-2.00 to -0.18) | HAMD | Adequate | Probable depression | Regular |
| Maldonado-Lopez | 1982 | 16 | CBT=8, ACT=8 | | CBT vs. ACT: -0.55 (-1.50 to 0.40) | BDI, HAMD, SDS | Adequate | Probable depression | Regular |
| Malouff | 1988 | 41 | WL=13, PST=14, CBT=14 | | PST vs. WL: -1.20 (-2.01 to -0.40)  CBT vs. WL: -1.40 (-2.22 to -0.57) | BDI, POMS-depression | Adequate | Probable depression | Specific |
| Manson | 1995 | 48 | WL=26, CBT=22 | | CBT vs. WL: -0.77 (-1.35 to -0.19) | CES-D | Adequate | Probable depression | Specific |
| Markowitz | 1998 | 75 | SUP=24, CBT=27, IPT=24 | | CBT vs. SUP: 0.06 (-0.48 to 0.60)  IPT vs. SUP: -0.53 (-1.10 to 0.04) | BDI, HAMD | Adequate | Probable depression | Specific |

Table S1

Description of individual studies (patient characteristics and outcome) (continued)

| First author | Year | Total number of patients | Number of patients per group | Effect Size and CI | | Outcome measure | Outcome assessment | Diagnosis | Patient population |
| --- | --- | --- | --- | --- | --- | --- | --- | --- | --- |
| Markowitz | 2005 | 49 | SUP=26, IPT=23 | | IPT vs. SUP: -0.05 (-0.60 to 0.50) | BDI, CDRS, HAMD | Adequate | Probable depression | Regular |
| Markowitz | 2008 | 26 | SUP=12, IPT=14 | | IPT vs. SUP: -0.60 (-1.37 to 0.16) | BDI, CDRS, HAMD24 | Adequate | Probable depression | Specific |
| Marshall | 2008 | 72 | CBT=37, IPT=35 | | CBT vs. IPT: -0.22 (-.068 to 0.24) | HAMD | Inadequate or not reported | Formal diagnosis | Regular |
| McBride | 2007 | 55 | CBT=28, IPT=27 | | IPT vs. CBT: 0.00 (-0.52 to 0.52) | BDI-II | Inadequate or not reported | Formal diagnosis | Regular |
| McKendree | 1998 | 57 | WL=21, CBT=36 | | CBT vs. WL: -0.34 (-0.88 to 0.19) | BDI, HAMD | Adequate | Probable depression | Regular |
| McLean | 1979 | 75 | PLA=37, DYN=38 | | DYN vs. PLA: 0.00 (-0.45 to 0.45) | BDI | Adequate | Formal diagnosis | Regular |
| McNamara | 1986 | 30 | SUP=10, CBT=10, ACT=10 | | CBT vs. SUP: -0.60 (-1.47 to 0.26)  ACT vs. SUP: -0.83 (-1.72 to 0.05) | BDI | Adequate | Probable depression | Regular |
| Meager | 1996 | 12 | WL=6, CBT=6 | | CBT vs. WL: -1.02 (-2.15 to 0.12) | BDI, EPDS, POMS-depression | Adequate | Probable depression | Specific |
| Milgrom | 2005 | 192 | UC=33, SUP=113, CBT=46 | | SUP vs. UC: -0.27 (-0.66 to 0.12)  CBT vs. SUP: 0.00 (-0.34 to 0.34) | BDI | Adequate | Probable depression | Specific |
| Milgrom | 2011 | 68 | CBT=45,  UC=23 | | CBT vs. UC: -0.36 (-0.86 to 0.14) | BDI-II | Adequate | Probable depression | Specific |
| Miller | 2002 | 30 | UC=15, IPT=15 | | IPT vs. UC: -0.45 (-1.15 to 0.26) | HAMD | Adequate | Probable depression | Regular |
| Miranda | 2003 | 179 | UC=89, CBT=90 | | CBT vs. UC: -0.16 (-0.45 to 0.13) | HAMD | Adequate | Formal diagnosis | Specific |
| Mitchell | 2009 | 98 | CBT=45  UC=53 | | CBT vs. UC: -1.16 (-1.59 to -0.73) | HAMD | Adequate | Formal diagnosis | Specific |
| Mohr | 2000 | 32 | UC=16, CBT=16 | | CBT vs. UC: -0.57 (-1.26 to 0.13) | POMS | Adequate | Probable depression | Specific |
| Mohr | 2005 | 122 | SUP=62, CBT=60 | | CBT vs. SUP: -0.39 (-0.74 to -0.03) | BDI-II, HAMD | Adequate | Probable depression | Specific |
| Mohr | 2011 | 32 | CBT=16,  UC=16 | | CBT vs. UC: -0.28 (-0.96 to 0.40) | PHQ-9, HAMD | Adequate | Formal diagnosis | Specific |
| Morris | 1975 | 34 | WL=12, CBT=22 | | CBT vs. WL: -1.79 (-2.60 to -0.97) | BDI, ZUNG | Adequate | Probable depression | Regular |
| Mossey | 1996 | 69 | UC=38, IPT=31 | | IPT vs. UC: -0.50 (-0.98 to -0.03) | GDS | Adequate | Probable depression | Specific |

Table S1

Description of individual studies (patient characteristics and outcome) (continued)

| First author | Year | Total number of patients | Number of patients per group | Effect Size and CI | | Outcome measure | Outcome assessment | Diagnosis | Patient population |
| --- | --- | --- | --- | --- | --- | --- | --- | --- | --- |
| Mukhtar | 2011 | 113 | CBT=58,  WL=55 | | CBT vs. WL: -0.65 (-1.02 to -0.27) | BDI | Adequate | Formal diagnosis | Regular |
| Mulcahy | 2010 | 50 | UC=27, IPT=23 | | IPT vs. UC: -0.59 (-1.15 to -0.03) | BDI-II, EPDS, HAMD | Adequate | Formal diagnosis | Specific |
| Murphy | 1995 | 24 | PLA=13, CBT=11 | | CBT vs. PLA: -0.30 (-1.08 to 0.48) | BDI, HAMD | Inadequate or not reported | Formal diagnosis | Regular |
| Murray | 2003 | 134 | SUP=47, DYN=45, CBT=42 | | DYN vs. SUP: -0.19 (-0.59 to 0.22)  CBT vs. SUP: -0.13 (-0.54 to 0.28) | EPDS | Adequate | Formal diagnosis | Specific |
| Mynors-Wallis | 1995 | 60 | PLA=30, PST=30 | | PST vs. PLA: -0.73 (-1.25 to -0.21) | BDI, HAMD | Adequate | Formal diagnosis | Regular |
| Neimeyer | 1990 | 102 | CBT=63, IPT=39 | | IPT vs. CBT: 0.00 (-0.40 to 0.40) | BDI, HAMD | Adequate | Formal diagnosis | Regular |
| Neugebauer | 2006 | 19 | UC=9, IPT=10 | | IPT vs. UC: -0.15 (-1.01 to 0.71) | HAMD | Inadequate or not reported | Probable depression | Specific |
| Nezu | 1986 | 17 | WL=6, PST=11 | | PST vs. WL: -2.98 (-4.40 to -1.56) | BDI | Adequate | Probable depression | Regular |
| Nezu | 1989 | 34 | WL=6, PST=28 | | PST vs. WL: -2.4 (-3.44 to -1.35) | BDI | Adequate | Formal diagnosis | Regular |
| O'Hara | 2000 | 99 | WL=51, IPT=48 | | IPT vs. WL: -1.14 (-1.56 to -0.72) | BDI, HAMD | Inadequate or not reported | Formal diagnosis | Specific |
| Pace | 1993 | 74 | WL=43, CBT=31 | | CBT vs. WL: -0.74 (-1.21 to -0.27) | BDI | Adequate | Probable depression | Specific |
| Padfield | 1976 | 24 | SUP=12, ACT=12 | | ACT vs. SUP: -0.70 (-1.49 to 0.10) | ZUNG | Adequate | Probable depression | Specific |
| Pecheur | 1984 | 21 | WL=7, CBT=14 | | CBT vs. WL: -1.98 (-3.06 to -0.91) | BDI, HAMD | Inadequate or not reported | Formal diagnosis | Specific |
| Peden | 2000 | 92 | UC=46, CBT=46 | | CBT vs. UC: -0.68 (-1.10 to -0.27) | BDI, CES-D | Adequate | Probable depression | Specific |
| Pibernik | 2009 | 50 | UC=25, CBT=25 | | CBT vs. UC: -0.19 (-0.74 to 0.35) | CES-D | Adequate | Probable depression | Specific |
| Power | 2012 | 49 | IPT=39,  UC=10 | | IPT vs. UC: -0.66 (-1.36 to 0.04) | BDI-II | Adequate | Formal diagnosis | Regular |
| Power | 2012 | 32 | CBT=22,  UC=10 | | CBT vs. UC: -0.36 (-1.10 to 0.37) | BDI-II | Adequate | Formal diagnosis | Regular |
| Prendergast | 2001 | 37 | PLA=20, CBT=17 | | CBT vs. PLA: -0.10 (-0.73 to 0.54) | EPDS, MADRS | Inadequate or not reported | Probable depression | Specific |
| Propst | 1992 | 49 | WL=11, CBT=38 | | CBT vs. WL: -0.90 (-1.59 to -0.22) | BDI, HAMD | Adequate | Probable depression | Regular |

Table S1

Description of individual studies (patient characteristics and outcome) (continued)

| First author | Year | Total number of patients | Number of patients per group | Effect Size and CI | | Outcome measure | Outcome assessment | Diagnosis | Patient population |
| --- | --- | --- | --- | --- | --- | --- | --- | --- | --- |
| Rahman | 2008 | 818 | PLA=400, CBT=418 | | CBT vs. PLA: -0.62 (-0.76 to -0.48) | HAMD | Adequate | Formal diagnosis | Specific |
| Ransom | 2008 | 79 | UC=38, IPT=41 | | IPT vs. UC: -0.16 (-0.60 to 0.28) | BDI-II | Adequate | Probable depression | Specific |
| Rohan | 2007 | 30 | WL=15, CBT=15 | | CBT vs. WL: -0.96 (-1.70 to -0.22) | BDI-II, HAMD, SIGH-SAD | Adequate | Formal diagnosis | Regular |
| Rohen | 2002 | 27 | WL=13, CBT=14 | | CBT vs. WL: -1.21 (-2.02 to -0.41) | BDI, HAMD | Adequate | Probable depression | Regular |
| Ross | 1985 | 51 | WL=21, CBT=30 | | CBT vs. WL: -0.65 (-1.21 to -0.09) | BDI, MADRS | Adequate | Formal diagnosis | Regular |
| Rude | 1986 | 44 | WL=16, SST=14, CBT=14 | | SST vs. WL: -0.97 (-1.72 to -0.23)  CBT vs. WL: -0.72 (-1.44 to 0.00) | BDI | Adequate | Probable depression | Regular |
| Safren | 2009 | 45 | WL=22, CBT=23 | | CBT vs. WL: -0.63 (-1.22 to -0.04) | BDI, HAMD | Adequate | Probable depression | Specific |
| Savard | 2006 | 37 | WL=16, CBT=21 | | CBT vs. WL: -0.68 (-1.34 to -0.03) | BDI, HADS-D, HAMD | Adequate | Probable depression | Specific |
| Schmidt | 1983 | 56 | WL=10, CBT=46 | | CBT vs. WL: -1.18 (-1.89 to -0.47) | BDI, DACL, MMPI, POMS, SDS | Adequate | Probable depression | Regular |
| Schmitt | 1988 | 30 | WL=6, SST=10, PST=14 | | SST vs. WL: -0.84 (-1.85 to 0.16)  PST vs. WL: -0.90 (-1.86 to 0.06) | BDI, HAMD | Inadequate or not reported | Probable depression | Regular |
| Schulberg | 1996 | 185 | UC=92, IPT=93 | | IPT vs. UC: -0.44 (-0.73 to -0.15) | HAMD | Adequate | Formal diagnosis | Regular |
| Scogin | 1987 | 17 | WL=8, CBT=9 | | CBT vs. WL: -1.27 (-2.28 to -0.26) | BDI, GDS, HAMD | Inadequate or not reported | Probable depression | Specific |
| Scogin | 1989 | 51 | WL=11, CBT=40 | | CBT vs. WL: -0.76 (-1.43 to 0.08) | GDS, HAMD | Inadequate or not reported | Probable depression | Specific |
| Scott | 1990 | 67 | WL=23, CBT=44 | | CBT vs. WL: -0.68 (-1.19 to -0.16) | BDI | Adequate | Probable depression | Regular |
| Scott | 1992 | 87 | UC=29, SUP=29, CBT=29 | | SUP vs. UC: -0.52 (-1.04 to -0.01)  CBT vs. UC: -0.25 (-0.76 to 0.26) | HAMD | Adequate | Formal diagnosis | Regular |
| Scott | 1997 | 34 | UC=16, CBT=18 | | CBT vs. UC: -0.48 (-1.15 to 0.19) | BDI, HAMD | Adequate | Formal diagnosis | Regular |
| Selmi | 1990 | 36 | WL=12, CBT=24 | | CBT vs. WL: -1.16 (-1.89 to -0.43) | HAMD | Adequate | Probable depression | Regular |

Table S1

Description of individual studies (patient characteristics and outcome) (continued)

| First author | Year | Total number of patients | Number of patients per group | Effect Size and CI | | Outcome measure | Outcome assessment | Diagnosis | Patient population |
| --- | --- | --- | --- | --- | --- | --- | --- | --- | --- |
| Serfaty | 2009 | 137 | UC=68.5, CBT=68.5 | | CBT vs. UC: -0.24 (-0.57 to 0.09) | BDI-II | Adequate | Probable depression | Specific |
| Shaw | 1977 | 32 | WL=8, SUP=8, CBT=8, ACT=8 | | SUP vs. WL: -0.43 (-1.37 to 0.51)  CBT vs. WL: -1.33 (-2.38 to -0.28)  ACT vs. WL: -0.43 (-1.37 to 0.51) | BDI, HAMD | Adequate | Probable depression | Specific |
| Sheeber | 2012 | 70 | CBT=35,  WL=35 | | CBT vs. WL: -0.84 (-1.32 to -0.36) | BDI-II | Adequate | Probable depression | Specific |
| Simpson | 2003 | 145 | UC=72, DYN=73 | | DYN vs. UC: 0.06 (-0.26 to 0.38) | BDI | Adequate | Probable depression | Regular |
| Simson | 2008 | 30 | UC=15, SUP=15 | | SUP vs. UC: -0.23 (-0.93 to 0.47) | HADS | Adequate | Probable depression | Specific |
| Skinner | 1983 | 24 | PLA=9, CBT=7, ACT=8 | | CBT vs. PLA: -0.53 (-1.48 to 0.42)  ACT vs. PLA: -0.65 (-1.58 to 0.29) | BDI | Adequate | Probable depression | Regular |
| Sloane | 1985 | 33 | PLA=14, IPT=19 | | IPT vs. PLA: 0 (-0.67 to 0.67) | BDI, HAMD | Adequate | Formal diagnosis | Specific |
| Snarski | 2011 | 29 | ACT=16,  UC=13 | | ACT vs. UC: 0.12 (-0.60 to 0.83) | GDS | Adequate | Probable depression | Specific |
| Spek | 2007 | 199 | WL=100, CBT=99 | | CBT vs. WL: -0.31 (-0.59 to -0.03) | BDI | Adequate | Probable depression | Specific |
| Spinelli | 2003 | 38 | PLA=17, IPT=21 | | IPT vs. PLA: -0.74 (-1.39 to -0.09) | BDI, EPDS, HAMD | Inadequate or not reported | Formal diagnosis | Specific |
| Sudweeks | 1996 | 45 | WL=15, CBT=30 | | CBT vs. WL: -1.44 (-2.12 to -0.76) | BDI | Adequate | Probable depression | Regular |
| Swartz | 2008 | 40 | UC=17, IPT=23 | | IPT vs. UC: -0.85 (-1.50 to -0.21) | BDI, HAMD | Adequate | Formal diagnosis | Specific |
| Talbot | 2011 | 58 | IPT=34,  UC=24 | | IPT vs. UC: -0.31 (-0.82 to 0.21) | BDI-II, HAMD | Inadequate or not reported | Formal diagnosis | Specific |
| Taylor | 1977 | 28 | WL=7, CBT=14, ACT=7 | | CBT vs. WL: -2.07 (-3.16 to -0.98)  ACT vs. WL: -1.25 (-2.36 to -0.15) | BDI | Adequate | Probable depression | Specific |
| Taylor | 2009 | 48 | WL=25, CBT=23 | | CBT vs. WL: -1.88 (-2.56 to -1.21) | HAMD, BDI | Adequate | Probable depression | Specific |
| Teasdale | 1984 | 34 | UC=17, CBT=17 | | CBT vs. UC: -0.85 (-1.54 to -0.16) | BDI, HAMD, MADRS | Adequate | Formal diagnosis | Regular |
| Teichmann | 1995 | 45 | WL=15, CBT=30 | | CBT vs. WL: -0.75 (-1.38 to -0.12) | BDI | Adequate | Probable depression | Regular |

Table S1

Description of individual studies (patient characteristics and outcome) (continued)

| First author | Year | Total number of patients | Number of patients per group | Effect Size and CI | | Outcome measure | Outcome assessment | Diagnosis | Patient population |
| --- | --- | --- | --- | --- | --- | --- | --- | --- | --- |
| Teri | 1997 | 62 | WL=20, PST=19, ACT=23 | | PST vs. WL: -1.14 (-1.80 to -0.47)  ACT vs. WL: -0.83 (-1.45 to -0.22) | BDI, CSDD, HAMD | Adequate | Probable depression | Specific |
| Thompson | 1984 | 33 | DYN=11, CBT=8, ACT=14 | | CBT vs. DYN: -0.61 (-1.51 to 0.28)  ACT vs. DYN: -0.14 (-0.90 to 0.63) | BDI, HAMD | Inadequate or not reported | Formal diagnosis | Specific |
| Thompson | 1987 | 74 | DYN=24, CBT=25, ACT=25 | | CBT vs. DYN: -0.13 (-0.68 to 0.42)  ACT vs. DYN: -0.20 (-0.75 to 0.36) | BDI, BSID, GDS, HAMD | Inadequate or not reported | Formal diagnosis | Specific |
| Thompson | 2010 | 53 | UC=27, CBT=26 | | CBT vs. UC: -0.92 (-1.48 to -0.36) | BDI | Adequate | Probable depression | Specific |
| Titov | 2010 | 127 | WL=40, CBT=87 | | CBT vs. WL: -1.16 (-1.56 to -0.76) | BDI-II, K10, PHQ9, SDS | Adequate | Formal diagnosis | Regular |
| Turner | 1979 | 28 | PLA=14, ACT=14 | | ACT vs. PLA: -0.77 (-1.52 to -0.02) | DACL | Adequate | Probable depression | Regular |
| Usaf | 1990 | 40 | WL=15, CBT=25 | | CBT vs. WL: 0.00 (-0.63 to 0.63) | BDI | Adequate | Formal diagnosis | Regular |
| Van Bastelaar | 2011 | 255 | CBT=125,  WL=130 | | CBT vs. WL: -0.49 (-0.74 to -0.24) | CES-D | Adequate | Probable depression | Specific |
| VanSchaik | 2006 | 143 | UC=74, IPT=69 | | IPT vs. UC: -0.18 (-0.51 to 0.15) | GDS, MADRS | Adequate | Formal diagnosis | Specific |
| Verduyn | 2003 | 87 | UC=13, SUP=44, CBT=30 | | SUP vs. UC: -0.08 (-0.69 to 0.53)  CBT vs. UC: -0.24 (-0.88 to 0.41) | BDI, HAMD | Adequate | Probable depression | Specific |
| Vernmark | 2010 | 88 | WL=29, CBT=59 | | CBT vs. WL: -0.82 (-1.28 to -0.36) | BDI, MADRS | Adequate | Formal diagnosis | Regular |
| Vitriol | 2009 | 87 | UC=43, DYN=44 | | DYN vs. UC: -0.57 (-0.99 to -0.14) | HAMD | Adequate | Probable depression | Specific |
| Warmerdam | 2008 | 263 | WL=87, PST=88, CBT=88 | | PST vs. WL: -0.47 (-0.77 to -0.17)  CBT vs. WL: -0.55 (-0.85 to -0.25) | CES-D | Adequate | Probable depression | Regular |
| Watkins | 2009 | 40 | WL=20, ACT=20 | | Act vs. WL: -1.11 (-1.76 to -0.45) | BDI-II, HAMD | Inadequate or not reported | Probable depression | Regular |
| Weissman | 1979 | 38 | PLA=21, IPT=17 | | IPT vs. PLA: -0.41 (-1.04 to 0.22) | RASKIN | Adequate | Formal diagnosis | Regular |
| Wickberg | 1996 | 41 | UC=21, SUP=20 | | SUP vs. UC: -0.82 (-1.45 to -0.20) | MADRS | Adequate | Formal diagnosis | Specific |
| Wiklund | 2010 | 67 | UC=34, CBT=33 | | CBT vs. UC: -0.05 (-0.52 to 0.42) | EPDS | Adequate | Probable depression | Specific |

Table S1

Description of individual studies (patient characteristics and outcome) (continued)

| First author | Year | Total number of patients | Number of patients per group | Effect Size and CI | | Outcome measure | Outcome assessment | Diagnosis | Patient population |
| --- | --- | --- | --- | --- | --- | --- | --- | --- | --- |
| Williams | 2000 | 278 | PLA=140, PST=138 | | PST vs. PLA: -0.80 (-0.43 to 0.04) | HSCL | Adequate | Probable depression | Specific |
| Wilson | 1983 | 25 | WL=9, CBT=8, ACT=8 | | CBT vs. WL: -1.73 (-2.82 to -0.63)  ACT vs. WL: -2.21 (-3.41 to -1.01) | BDI, HAMD | Inadequate or not reported | Probable depression | Regular |
| Wilson | 1990 | 10 | SUP=5, CBT=5 | | CBT vs. SUP: 0.20 (-0.93 to 1.32) | BDI, MMPI-D | Inadequate or not reported | Probable depression | Specific |
| Wollersheim | 1991 | 32 | WL=8, SUP=8, CBT=16 | | SUP vs. WL: -0.34 (-1.28 to 0.59)  CBT vs. WL: -0.28 (-1.11 to 0.54) | BDI, MMPI-D | Adequate | Formal diagnosis | Regular |
| Wong | 2008 | 88 | WL=40, CBT=48 | | CBT vs. WL: -0.76 (-1.19 to -0.33) | C-BDI | Adequate | Formal diagnosis | Regular |
| Wong | 2008 | 322 | WL=159, CBT=163 | | CBT vs. WL: -74 (-0.96 to -0.51) | C-BDI | Adequate | Formal diagnosis | Regular |
| Wright | 2005 | 45 | WL=15, CBT=30 | | CBT vs. WL: -1.12 (-1.77 to -0.46) | BDI, HAMD | Adequate | Formal diagnosis | Regular |
| Zeiss | 1979 | 28 | WL=7, SST=7, CBT=7, ACT=7 | | SST vs. WL: -0.58 (-1.59 to 0.43)  CBT vs. WL: -0.58 (-1.59 to 0.43)  ACT vs. WL: -0.58 (-1.59 to 0.43) | MMPI-D | Adequate | Probable depression | Regular |

*Note.* ACT = Behavioral activation, BDI = Beck depression inventory, BDI-II = Beck depression inventor second Edition, CBT = Cognitive behavioural therapy, C-BDI = Chinese version of the Beck depression inventory, CES-D = Center for epidemiologic studies depression scale, CDS = Cardiac depression scale, CDRS = Cornell dysthymia rating scale, CI = Confidence interval*,* CMID = Cornell medical index depression, CSDD = Cornell scale for depression and dementia, DACL = Depression adjective checklist, DYN = Psychodynamic therapy, Effect size = Effect size between groups (Hedges g), EPDS = Edinburgh postnatal depression scale, Formal diagnosis = Diagnosis according to DSM or ICD with clinical interview, GDS = Geriatric depression scale, HADS = Hospital anxiety and depression scale, HADS-D = Hospital anxiety and depression scale – depression subscale, HAMD = Hamilton depression scale, HSCL = Hopkins depression self-report scale, IDD = Inventory to diagnose depression, IDS = Inventar depressiver Symptome, IPT = Interpersonal therapy, ITT = intention-to-treat, K6/ K10 = Kessler 6/10, MADRS = Montgomery Asberg depression rating scale, MMPI-D = Minnesota multiphasic personality inventory – depression subscale, PHQ = Patient health questionnaire, PLA = Placebo, POMS = Profile of mood state, Probable depression = Patients with elevated depression score, minor depression or other mood disorder, PST = Problem solving therapy, RASKIN = Raskin three area depression scale, Regular = Regular depression, SCL(90)D = Symptom check list depression subscale, SDS = Zung self-rating depression scale, SIGH-SAD = Structured interview guide for the Hamilton rating scale for depression—SAD, Specific = Specific population, SST = Social skill training, SUP = Supportive counselling, UC = Usual care, WDI = Wakefield self-assessment of depression inventory, WL = Waitlist, ZUNG = Zung self rating depression scale.

# Table S2

Description of individual studies (methodological characteristics and intervention)

| First author | Year | Publication period | Sample size | Concealment of allocation | Type of analysis | Intervention format and setting | Treatment dose  (n sessions) |
| --- | --- | --- | --- | --- | --- | --- | --- |
| Alexopoulos | 2003 | recent | Small | Inadequate or not reported | ITT | Individual and face to face | High (12) |
| Allart | 2003 | recent | Large | Inadequate or not reported | ITT | Other | High (12) |
| Andersson | 2005 | recent | Moderate | Adequate | Completer | Other | Low (5) |
| Arean | 1993 | early | Small | Inadequate or not reported | Completer | Other | High (12) |
| Arean | 2010 | recent | Large | Inadequate or not reported | ITT | Individual and face to face | High (12) |
| Ayen | 2004 | recent | Small | Inadequate or not reported | ITT | Other | High (12) |
| Baker | 2009 | recent | Large | Inadequate or not reported | ITT | Individual and face to face | High (10) |
| Barnhofer | 2009 | recent | Small | Inadequate or not reported | ITT | Other | High (8) |
| Barrera | 1979 | early | Small | Inadequate or not reported | Completer | Other | High (8) |
| Barrett | 2001 | recent | Large | Adequate | ITT | Individual and face to face | Low (6) |
| Beach | 1992 | early | Small | Inadequate or not reported | Completer | Individual and face to face | High (18) |
| Beeber | 2010 | recent | Moderate | Inadequate or not reported | Completer | Individual and face to face | High (16) |
| Bellack | 1981 | early | Small | Inadequate or not reported | Completer | Individual and face to face | High (12) |
| Berger | 2011 | recent | Moderate | Adequate | ITT | Other | High (10) |
| Beutler | 1991 | early | Small | Inadequate or not reported | ITT | Other | High (20) |
| Bodenmann | 2008 | recent | Small | Adequate | Completer | Individual and face to face | High (20) |
| Bolton | 2003 | recent | Large | Inadequate or not reported | ITT | Other | High (16) |
| Bowman | 1995 | early | Small | Inadequate or not reported | Completer | Other | Low (4) |
| Bright | 1999 | early | Moderate | Inadequate or not reported | Completer | Other | High (10) |

Table S2

*Description of individual studies* (methodological characteristics and intervention) *(continued)*

| First author | Year | Publication period | Sample size | Concealment of allocation | Type of analysis | Intervention format and setting | Treatment dose  (n sessions) |
| --- | --- | --- | --- | --- | --- | --- | --- |
| Brown | 1984 | early | Moderate | Inadequate or not reported | Completer | Other | High (12) |
| Carpenter | 2008 | recent | Small | Inadequate or not reported | ITT | Individual and face to face | High (24) |
| Carrington | 1979 | early | Small | Inadequate or not reported | Completer | Individual and face to face | High (12) |
| Castonguay | 2004 | recent | Small | Inadequate or not reported | Completer | Individual and face to face | High (16) |
| Chen | 2000 | recent | Moderate | Inadequate or not reported | Completer | Other | Low (4) |
| Cho | 2008 | recent | Small | Inadequate or not reported | Completer | Individual and face to face | High (9) |
| Choi | 2012 | recent | Moderate | Adequate | ITT | Other | Low (6) |
| Christensen | 2004 | recent | Large | Adequate | ITT | Other | Low (6) |
| Collins | 1996 | early | Moderate | Inadequate or not reported | Completer | Other | High (12) |
| Comas-Diaz | 1981 | early | Small | Inadequate or not reported | Completer | Other | Low (5) |
| Conoley | 1985 | early | Small | Inadequate or not reported | Completer | Individual and face to face | Low (2) |
| Conradi | 2007 | recent | Large | Inadequate or not reported | ITT | Individual and face to face | High (14) |
| Cooper | 2003 | recent | Moderate | Inadequate or not reported | ITT | Individual and face to face | High (10) |
| Covi | 1987 | early | Small | Inadequate or not reported | Completer | Other | High (15) |
| Cramer | 2011 | recent | Moderate | Inadequate or not reported | Completer | Other | High (12) |
| Cullen | 2002 | recent | Small | Inadequate or not reported | ITT | Individual and face to face | High (10) |
| Dekker | 2012 | recent | Small | Adequate | ITT | Individual and face to face | Low (1) |
| DeRubeis | 2005 | recent | Large | Inadequate or not reported | ITT | Individual and face to face | High (14) |
| Dimidjian | 2006 | recent | Moderate | Inadequate or not reported | ITT | Individual and face to face | High (16) |
| Dobkin | 2011 | recent | Moderate | Inadequate or not reported | Completer | Individual and face to face | High (10) |

Table S2

*Description of individual studies* (methodological characteristics and intervention) *(continued)*

| First author | Year | Publication period | Sample size | Concealment of allocation | Type of analysis | Intervention format and setting | | Treatment dose  (n sessions) | |  |
| --- | --- | --- | --- | --- | --- | --- | --- | --- | --- | --- |
| Dowrick | 2000 | recent | Large | Adequate | ITT | | Individual and face to face | | Low (6) | |
| Duarte | 2009 | recent | Moderate | Inadequate or not reported | Completer | | Other | | High (12) | |
| Duchac | 2002 | recent | Small | Inadequate or not reported | ITT | Other | | Low (6) | |  |
| Dwight | 2011 | recent | Large | Inadequate or not reported | Completer | Other | | High (8) | |  |
| Ekers | 2011 | recent | Small | Adequate | ITT | Individual and face to face | | High (12) | |  |
| Elkin | 1989 | early | Large | Inadequate or not reported | ITT | Individual and face to face | | High (16) | |  |
| Epstein | 1986 | early | Small | Inadequate or not reported | Completer | Other | | High (8) | |  |
| Evans | 1995 | early | Small | Inadequate or not reported | Completer | Other | | High (8) | |  |
| Faramarzi | 2008 | recent | Moderate | Inadequate or not reported | Completer | Other | | High (10) | |  |
| Fleming | 1980 | early | Small | Inadequate or not reported | Completer | Other | | High (8) | |  |
| Floyd | 2004 | recent | Small | Inadequate or not reported | Completer | Other | | Low (4) | |  |
| Forsyth | 2000 | recent | Moderate | Inadequate or not reported | Completer | Other | | Low (4) | |  |
| Foster | 2007 | recent | Moderate | Inadequate or not reported | Completer | Other | | High (16) | |  |
| Freedland | 2009 | recent | Moderate | Inadequate or not reported | ITT | Individual and face to face | | High (12) | |  |
| Frothingham | 2005 | recent | Small | Inadequate or not reported | Completer | Other | | High (8) | |  |
| Fry | 1984 | early | Small | Inadequate or not reported | Completer | Individual and face to face | | High (12) | |  |
| Fuchs | 1977 | early | Small | Inadequate or not reported | Completer | Other | | Low (6) | |  |
| Furukawa | 2012 | recent | Large | Adequate | ITT | Other | | High (8) | |  |
| Gallagher | 1981 | early | Small | Inadequate or not reported | Completer | Other | | High (10) | |  |
| Gallagher | 1982 | early | Small | Inadequate or not reported | Completer | Individual and face to face | | High (16) | |  |

Table S2

*Description of individual studies* (methodological characteristics and intervention) *(continued)*

| First author | Year | Publication period | Sample size | Concealment of allocation | Type of analysis | Intervention format and setting | Treatment dose  (n sessions) |
| --- | --- | --- | --- | --- | --- | --- | --- |
| Gallagher | 1994 | early | Moderate | Inadequate or not reported | Completer | Individual and face to face | High (20) |
| Gardner | 1981 | early | Small | Inadequate or not reported | Completer | Individual and face to face | Low (6) |
| Grote | 2009 | recent | Moderate | Inadequate or not reported | Completer | Individual and face to face | High (8) |
| Hamamci | 2006 | recent | Small | Inadequate or not reported | Completer | Other | High (11) |
| Hamdan-Mansour | 2009 | recent | Moderate | Adequate | Completer | Other | High (10) |
| Haringsma | 2005 | recent | Large | Inadequate or not reported | ITT | Other | High (10) |
| Hautzinger | 2004 | recent | Moderate | Adequate | ITT | Other | High (12) |
| Hautzinger | 2008 | recent | Large | Inadequate or not reported | ITT | Other | High (15) |
| Hayden | 2012 | recent | Small | Adequate | ITT | Individual and face to face | High (10) |
| Hayman | 1980 | early | Small | Inadequate or not reported | Completer | Other | High (8) |
| Hegerl | 2009 | recent | Large | Adequate | ITT | Other | High (10) |
| Holden | 1989 | early | Moderate | Inadequate or not reported | Completer | Individual and face to face | High (8) |
| Honey | 2002 | recent | Small | Inadequate or not reported | ITT | Other | High (8) |
| Hopko | 2011 | recent | Moderate | Adequate | ITT | Individual and face to face | High (8) |
| Hunter | 2012 | recent | Moderate | Inadequate or not reported | Completer | Other | High (18) |
| Jacobson | 1996 | early | Moderate | Inadequate or not reported | Completer | Individual and face to face | High (20) |
| Jamison | 1995 | early | Moderate | Inadequate or not reported | Completer | Other | Low (4) |
| Jarrett | 1999 | early | Moderate | Inadequate or not reported | ITT | Individual and face to face | High (20) |
| Johansson | 2012 | recent | Moderate | Adequate | ITT | Other | High (10) |
| Johansson | 2012 | recent | Moderate | Adequate | ITT | Other | High (10) |

Table S2

*Description of individual studies* (methodological characteristics and intervention) *(continued)*

| First author | Year | Publication period | Sample size | Concealment of allocation | Type of analysis | Intervention format and setting | Treatment dose  (n sessions) |
| --- | --- | --- | --- | --- | --- | --- | --- |
| Johansson | 2012 | recent | Moderate | Adequate | ITT | Other | High (10) |
| Johnson | 2012 | recent | Small | Adequate | ITT | Other | High (24) |
| Joling | 2011 | recent | Large | Adequate | ITT | Other | Low (5) |
| Kay-Lambkin | 2009 | recent | Moderate | Adequate | Completer | Other | High (10) |
| Kelly | 1993 | early | Small | Inadequate or not reported | Completer | Other | High (8) |
| King | 2000 | recent | Large | Adequate | ITT | Individual and face to face | Low (6) |
| Kiosses | 2010 | recent | Small | Inadequate or not reported | ITT | Individual and face to face | High (12) |
| Klein | 1985 | early | Small | Inadequate or not reported | Completer | Other | High (12) |
| Kohen | 2009 | recent | Moderate | Adequate | ITT | Other | High (9) |
| Koszycky | 2012 | recent | Small | Inadequate or not reported | Completer | Individual and face to face | High (12) |
| Krampen | 1997 | early | Small | Inadequate or not reported | Completer | Individual and face to face | High (20) |
| Laidlaw | 2008 | recent | Small | Adequate | Completer | Individual and face to face | High (8) |
| Lamers | 2010 | recent | Large | Adequate | Completer | Individual and face to face | Low (6) |
| Landreville | 1997 | early | Small | Inadequate or not reported | Completer | Other | Low (4) |
| LaPointe | 1980 | early | Small | Inadequate or not reported | Completer | Other | Low (6) |
| Lexis | 2011 | recent | Large | Inadequate or not reported | Completer | Individual and face to face | High (8) |
| Lincoln | 2003 | recent | Moderate | Adequate | Completer | Individual and face to face | High (10) |
| Lopez | 2004 | recent | Small | Inadequate or not reported | Completer | Individual and face to face | High (12) |
| Lustman | 1998 | early | Small | Inadequate or not reported | ITT | Individual and face to face | High (10) |
| Lynch | 1997 | early | Small | Inadequate or not reported | Completer | Other | Low (6) |

Table S2

*Description of individual studies* (methodological characteristics and intervention) *(continued)*

| First author | Year | Publication period | Sample size | Concealment of allocation | Type of analysis | Intervention format and setting | Treatment dose  (n sessions) |
| --- | --- | --- | --- | --- | --- | --- | --- |
| Lynch | 2004 | recent | Small | Inadequate or not reported | Completer | Other | Low (6) |
| Maina | 2005 | recent | Small | Inadequate or not reported | ITT | Individual and face to face | High (20) |
| Maldonado-Lopez | 1982 | early | Small | Inadequate or not reported | Completer | Individual and face to face | High (10) |
| Malouff | 1988 | early | Small | Inadequate or not reported | Completer | Other | Low (4) |
| Manson | 1995 | early | Small | Inadequate or not reported | Completer | Other | High (16) |
| Markowitz | 1998 | early | Moderate | Inadequate or not reported | ITT | Individual and face to face | High (16) |
| Markowitz | 2005 | recent | Small | Inadequate or not reported | ITT | Individual and face to face | High (17) |
| Markowitz | 2008 | recent | Small | Inadequate or not reported | ITT | Individual and face to face | High (17) |
| Marshall | 2008 | recent | Moderate | Inadequate or not reported | Completer | Individual and face to face | High (16) |
| McBride | 2007 | recent | Moderate | Inadequate or not reported | Completer | Individual and face to face | High (16) |
| McKendree | 1998 | early | Moderate | Inadequate or not reported | Completer | Other | High (8) |
| McLean | 1979 | early | Moderate | Inadequate or not reported | Completer | Individual and face to face | High (10) |
| McNamara | 1986 | early | Small | Inadequate or not reported | Completer | Individual and face to face | High (9) |
| Meager | 1996 | early | Small | Inadequate or not reported | Completer | Other | High (10) |
| Milgrom | 2005 | recent | Large | Inadequate or not reported | ITT | Other | High (9) |
| Milgrom | 2011 | recent | Small | Inadequate or not reported | Completer | Individual and face to face | Low (6) |
| Milgrom | 2011 | recent | Small | Inadequate or not reported | Completer | Individual and face to face | Low (6) |
| Miller | 2002 | recent | Small | Inadequate or not reported | ITT | Other | High (12) |
| Miranda | 2003 | recent | Large | Adequate | ITT | Other | High (8) |
| Mitchell | 2009 | recent | Moderate | Adequate | ITT | Other | High (9) |

Table S2

*Description of individual studies* (methodological characteristics and intervention) *(continued)*

| First author | Year | Publication period | Sample size | Concealment of allocation | Type of analysis | Intervention format and setting | Treatment dose  (n sessions) |
| --- | --- | --- | --- | --- | --- | --- | --- |
| Mohr | 2000 | recent | Small | Inadequate or not reported | ITT | Other | High (8) |
| Mohr | 2005 | recent | Large | Inadequate or not reported | Completer | Other | High (16) |
| Mohr | 2011 | recent | Small | Inadequate or not reported | Completer | Other | High (16) |
| Morris | 1975 | early | Small | Inadequate or not reported | Completer | Other | Low (6) |
| Mossey | 1996 | early | Moderate | Inadequate or not reported | Completer | Individual and face to face | High (10) |
| Mukhtar | 2011 | recent | Large | Inadequate or not reported | Completer | Other | High (8) |
| Mulcahy | 2010 | recent | Moderate | Inadequate or not reported | Completer | Other | High (11) |
| Murphy | 1995 | early | Small | Inadequate or not reported | Completer | Individual and face to face | High (20) |
| Murray | 2003 | recent | Moderate | Inadequate or not reported | ITT | Individual and face to face | High (10) |
| Mynors-Wallis | 1995 | early | Moderate | Inadequate or not reported | ITT | Individual and face to face | Low (6) |
| Neimeyer | 1990 | early | Large | Inadequate or not reported | Completer | Other | High (10) |
| Neugebauer | 2006 | recent | Small | Inadequate or not reported | ITT | Individual and face to face | Low (6) |
| Nezu | 1986 | early | Small | Inadequate or not reported | Completer | Other | High (8) |
| Nezu | 1989 | early | Small | Inadequate or not reported | Completer | Other | High (10) |
| O'Hara | 2000 | recent | Moderate | Inadequate or not reported | ITT | Individual and face to face | High (12) |
| Pace | 1993 | early | Moderate | Inadequate or not reported | Completer | Individual and face to face | High (7) |
| Padfield | 1976 | early | Small | Inadequate or not reported | Completer | Individual and face to face | High (12) |
| Pecheur | 1984 | early | Small | Inadequate or not reported | ITT | Individual and face to face | High (8) |
| Peden | 2000 | recent | Moderate | Inadequate or not reported | Completer | Other | Low (6) |
| Power | 2012 | recent | Small | Inadequate or not reported | Completer | Individual and face to face | High (16) |

Table S2

*Description of individual studies* (methodological characteristics and intervention) *(continued)*

| First author | Year | Publication period | Sample size | Concealment of allocation | Type of analysis | Intervention format and setting | Treatment dose  (n sessions) |
| --- | --- | --- | --- | --- | --- | --- | --- |
| Power | 2012 | recent | Small | Inadequate or not reported | Completer | Individual and face to face | High (12) |
| Pibernik | 2009 | recent | Moderate | Inadequate or not reported | Completer | Other | Low (4) |
| Prendergast | 2001 | recent | Small | Inadequate or not reported | Completer | Individual and face to face | Low (6) |
| Propst | 1992 | early | Small | Inadequate or not reported | Completer | Individual and face to face | High (19) |
| Rahman | 2008 | recent | Large | Inadequate or not reported | ITT | Other | High (8) |
| Ransom | 2008 | recent | Moderate | Inadequate or not reported | ITT | Other | Low (6) |
| Rohan | 2007 | recent | Small | Inadequate or not reported | ITT | Other | High (12) |
| Rohen | 2002 | recent | Small | Inadequate or not reported | ITT | Other | Low (4) |
| Ross | 1985 | early | Moderate | Inadequate or not reported | Completer | Other | High (12) |
| Rude | 1986 | early | Small | Inadequate or not reported | Completer | Other | High (12) |
| Safren | 2009 | recent | Small | Inadequate or not reported | ITT | Individual and face to face | High (11) |
| Savard | 2006 | recent | Small | Inadequate or not reported | Completer | Individual and face to face | High (8) |
| Schmidt | 1983 | early | Moderate | Inadequate or not reported | Completer | Individual and face to face | High (8) |
| Schmitt | 1988 | early | Small | Inadequate or not reported | Completer | Other | High (12) |
| Schulberg | 1996 | early | Large | Inadequate or not reported | ITT | Individual and face to face | High (16) |
| Scogin | 1987 | early | Small | Inadequate or not reported | Completer | Other | Low (4) |
| Scogin | 1989 | early | Moderate | Inadequate or not reported | Completer | Other | Low (4) |
| Scott | 1990 | early | Moderate | Inadequate or not reported | ITT | Other | High (12) |
| Scott | 1992 | early | Moderate | Inadequate or not reported | ITT | Individual and face to face | High (16) |
| Scott | 1997 | early | Small | Inadequate or not reported | Completer | Individual and face to face | Low (6) |

Table S2

*Description of individual studies* (methodological characteristics and intervention) *(continued)*

| First author | Year | Publication period | Sample size | Concealment of allocation | Type of analysis | Intervention format and setting | Treatment dose  (n sessions) |
| --- | --- | --- | --- | --- | --- | --- | --- |
| Selmi | 1990 | early | Small | Inadequate or not reported | ITT | Individual and face to face | Low (6) |
| Serfaty | 2009 | recent | Large | Adequate | ITT | Individual and face to face | High (12) |
| Shaw | 1977 | early | Small | Inadequate or not reported | Completer | Other | High (8) |
| Sheeber | 2012 | recent | Moderate | Adequate | ITT | Other | High (8) |
| Simpson | 2003 | recent | Large | Adequate | ITT | Individual and face to face | Low (5) |
| Simson | 2008 | recent | Small | Inadequate or not reported | ITT | Individual and face to face | Low (5) |
| Skinner | 1983 | early | Small | Inadequate or not reported | Completer | Individual and face to face | Low (5) |
| Sloane | 1985 | early | Small | Inadequate or not reported | Completer | Individual and face to face | Low (6) |
| Snarski | 2011 | recent | Small | Inadequate or not reported | Completer | Individual and face to face | High (8) |
| Spek | 2007 | recent | Large | Adequate | ITT | Other | High (10) |
| Spinelli | 2003 | recent | Small | Inadequate or not reported | ITT | Individual and face to face | High (16) |
| Sudweeks | 1996 | early | Small | Inadequate or not reported | Completer | Other | Low (6) |
| Swartz | 2008 | recent | Small | Inadequate or not reported | ITT | Individual and face to face | High (8) |
| Talbot | 2011 | recent | Moderate | Inadequate or not reported | Completer | Individual and face to face | High (13) |
| Taylor | 1977 | early | Small | Inadequate or not reported | Completer | Other | Low (6) |
| Taylor | 2009 | recent | Small | Inadequate or not reported | ITT | Individual and face to face | High (15) |
| Teasdale | 1984 | early | Small | Inadequate or not reported | ITT | Individual and face to face | High (15) |
| Teichmann | 1995 | early | Small | Inadequate or not reported | Completer | Other | High (13) |
| Teri | 1997 | early | Small | Inadequate or not reported | Completer | Individual and face to face | High (9) |
| Thompson | 1984 | early | Small | Inadequate or not reported | Completer | Individual and face to face | High (18) |

Table S2

*Description of individual studies* (methodological characteristics and intervention) *(continued)*

| First author | Year | Publication period | Sample size | Concealment of allocation | Type of analysis | Intervention format and setting | Treatment dose  (n sessions) |
| --- | --- | --- | --- | --- | --- | --- | --- |
| Thompson | 1987 | early | Small | Inadequate or not reported | Completer | Individual and face to face | High (18) |
| Thompson | 2010 | recent | Moderate | Inadequate or not reported | Completer | Other | High (8) |
| Titov | 2010 | recent | Large | Inadequate or not reported | ITT | Other | Low (6) |
| Turner | 1979 | early | Small | Inadequate or not reported | Completer | Individual and face to face | Low (5) |
| Usaf | 1990 | early | Small | Inadequate or not reported | Completer | Other | High (10) |
| Van Bastelaar | 2011 | recent | Large | Adequate | ITT | Other | High (8) |
| VanSchaik | 2006 | recent | Large | Inadequate or not reported | ITT | Individual and face to face | High (10) |
| Verduyn | 2003 | recent | Moderate | Inadequate or not reported | Completer | Other | High (16) |
| Vernmark | 2010 | recent | Moderate | Adequate | ITT | Other | High (7) |
| Vitriol | 2009 | recent | Moderate | Inadequate or not reported | ITT | Individual and face to face | High (12) |
| Warmerdam | 2008 | recent | Large | Adequate | ITT | Other | High (8) |
| Watkins | 2009 | recent | Small | Inadequate or not reported | Completer | Other | High (8) |
| Weissman | 1979 | early | Small | Inadequate or not reported | Completer | Individual and face to face | High (16) |
| Wickberg | 1996 | early | Small | Inadequate or not reported | Completer | Individual and face to face | Low (6) |
| Wiklund | 2010 | recent | Moderate | Inadequate or not reported | Completer | Individual and face to face | High (21) |
| Williams | 2000 | recent | Large | Adequate | ITT | Individual and face to face | Low (6) |
| Wilson | 1983 | early | Small | Inadequate or not reported | Completer | Individual and face to face | High (8) |
| Wilson | 1990 | early | Small | Inadequate or not reported | Completer | Individual and face to face | High (14) |
| Wollersheim | 1991 | early | Small | Inadequate or not reported | Completer | Other | High (10) |
| Wong | 2008 | recent | Moderate | Inadequate or not reported | ITT | Other | High (10) |

Table S2

*Description of individual studies* (methodological characteristics and intervention) *(continued)*

| First author | Year | Publication period | Sample size | Concealment of allocation | Type of analysis | Intervention format and setting | Treatment dose  (n sessions) |
| --- | --- | --- | --- | --- | --- | --- | --- |
| Wright | 2005 | recent | Small | Inadequate or not reported | ITT | Individual and face to face | High (9) |
| Zeiss | 1979 | early | Small | Inadequate or not reported | Completer | Individual and face to face | High (12) |

# Table S3

Aspects of Study Quality in Small, Moderate, and Large Studies (column percentages)

|  |  | Sample size | | |  |
| --- | --- | --- | --- | --- | --- |
| Study quality aspect | Number of studies | Small | Moderate | Large | *p* for trend^a^ |
| Concealment of allocation  Adequate  Inadequate or not reported | 34  164 | 6 (5.8%)  97 (94.2%) | 12 (20.3%)  47 (79.7%) | 16 (44.4%)  20 (55.6%) | <.001 |
| Outcome assessment  Adequate  Inadequate or not reported | 169  29 | 81 (78.6%)  22 (21.4%) | 52 (88.1%)  7 (11.9%) | 36 (100.0%)  0 (0.0%) | <.001 |
| Type of analysis  Intention-to-treat  Completer | 91  107 | 34 (33.0%)  69 (67.0%) | 25 (42.4%)  34 (57.6%) | 32 (88.9%)  4 (11.1%) | <.001 |

^a^*p* for Somer’s *D* with sample size as an ordinal variable.

# Table S4

Relative effect sizes [and 95% credibility intervals] of psychotherapeutic interventions and control conditions from network meta-analyses restricted to at least moderately sized (upper triangle) and large (lower triangle) studies

|  | Waitlist | Usual care | Placebo | Supportive counselling | Psychodynamic therapy | Social skill training | Problem solving therapy | Cognitive-behavioural therapy | Behavioural activation | Interpersonal therapy |
| --- | --- | --- | --- | --- | --- | --- | --- | --- | --- | --- |
| Waitlist |  | **-0.24**  [-0.46, -0.03]  k=0 | -0.22  [-0.46, 0.02]  k=0 | **-0.51**  [-0.78, -0.26]  k=0 | **-0.60**  [-0.96, -0.24]  k=0 | no study | **-0.57**  [-0.85, -0.29]  k=1 | **-0.68**  [-0.84, -0.53]  k=23 | **-0.69**  [-1.22, -0.16]  k=0 | **-0.85**  [-1.12, -0.58]  k=2 |
| Usual care | -0.18  [-0.49, 0.12]  k=0 |  | 0.03  [-0.20, 0.25]  k=0 | **-0.27**  [-0.51, -0.04]  k=8 | **-0.36**  [-0.68, -0.02]  k=3 | no study | **-0.33**  [-0.58, -0.07]  k=1 | **-0.44**  [-0.59, -0.29]  k=22 | -0.44  [-0.97, 0.07]  k=0 | **-0.61**  [-0.82, -0.39]  k=9 |
| Placebo | -0.17  [-0.50, 0.15]  k=0 | 0.01  [-0.31, 0.31]  k=0 |  | **-0.30**  [-0.57, -0.02]  k=0 | **-0.38**  [-0.74, -0.03]  k=2 | no study | **-0.36**  [-0.61, -0.09]  k=3 | **-0.47**  [-0.65, -0.28]  k=10 | -0.47  [-0.99, 0.04]  k=1 | **-0.63**  [-0.92, -0.34]  k=1 |
| Supportive counselling | -0.29  [-0.67, 0.09]  k=0 | -0.11  [-0.46, 0.24]  k=2 | -0.12  [-0.49, 0.27]  k=0 |  | -0.09  [-0.45, 0.29]  k=2 | no study | -0.06  [-0.35, 0.24]  k=1 | -0.17  [-0.38, 0.05]  k=13 | -0.18  [-0.72, 0.37]  k=0 | **-0.34**  [-0.63, -0.03]  k=1 |
| Psychodynamic therapy | -0.12  [-0.92, 0.67]  k=0 | 0.06  [-0.67, 0.80]  k=1 | 0.05  [-0.74, 0.86]  k=0 | 0.17  [-0.64, 0.98]  k=0 |  | no study | 0.03  [-0.38, 0.42]  k=0 | -0.08  [-0.42, 0.25]  k=3 | -0.09  [-0.69, 0.51]  k=0 | -0.25  [-0.63, 0.14]  k=0 |
| Social skill training | no study | no study | no study | no study | no study |  | no study | no study | no study | no study |
| Problem solving therapy | **-0.46**  [-0.81, -0.12]  k=1 | -0.28  [-0.59, 0.03]  k=2 | **-**0.29  [-0.60, 0.03]  k=2 | -0.17  [-0.54, 0.20]  k=1 | -0.34  [-1.14, 0.45]  k=0 | no study |  | -0.11  [-0.36, 0.13]  k=2 | -0.12  [-0.64, 0.40]  k=1 | -0.28  [-0.60, 0.04]  k=0 |
| Cognitive-behavioural therapy | **-0.57**  [-0.80, -0.35]  k=10 | **-0.39**  [-0.62, -0.17]  k=9 | **-0.41**  [-0.64, -0.15]  k=4 | -0.29  [-0.60, 0.03]  k=5 | no study | no study | -0.11  [-0.40, 0.17]  k=2 |  | -0.01  [-0.51, 0.49]  k=2 | -0.17  [-0.39, 0.07]  k=5 |
| Behavioural activation | no study | no study | no study | no study | no study | no study | no study | no study |  | no study |
| Interpersonal therapy | **-0.73**  [-1.14, -0.32]  k=0 | **-0.55**  [-0.87, -0.23]  k=3 | **-0.56**  [-0.96, -0.14]  k=1 | -0.45  [-0.89, 0.02]  k=0 | -0.61  [-1.40, 0.19]  k=0 | no study | -0.27  [-0.68, 0.16]  k=0 | -0.16  [-0.51, 0.20]  k=2 | no study |  |

*Note.* Dashes indicate pairs of conditions that have not been compared in a study. Negative signs in the upper triangle indicate superiority of the condition in column; negative signs in the lower triangle indicate superiority of the condition in the row. Significant effects are printed in bold. k = number of comparisons.

# Figure S1

Forest plot of inconsistency in closed loops with 95% confidence interval.

a=waitlist; b=usual care; c=placebo; d=supportive counselling; e=psychodynamic therapy; f=social skill training; g=problem-solving therapy; h=cognitive behavioural therapy; i=behavioural activation; j=interpersonal therapy


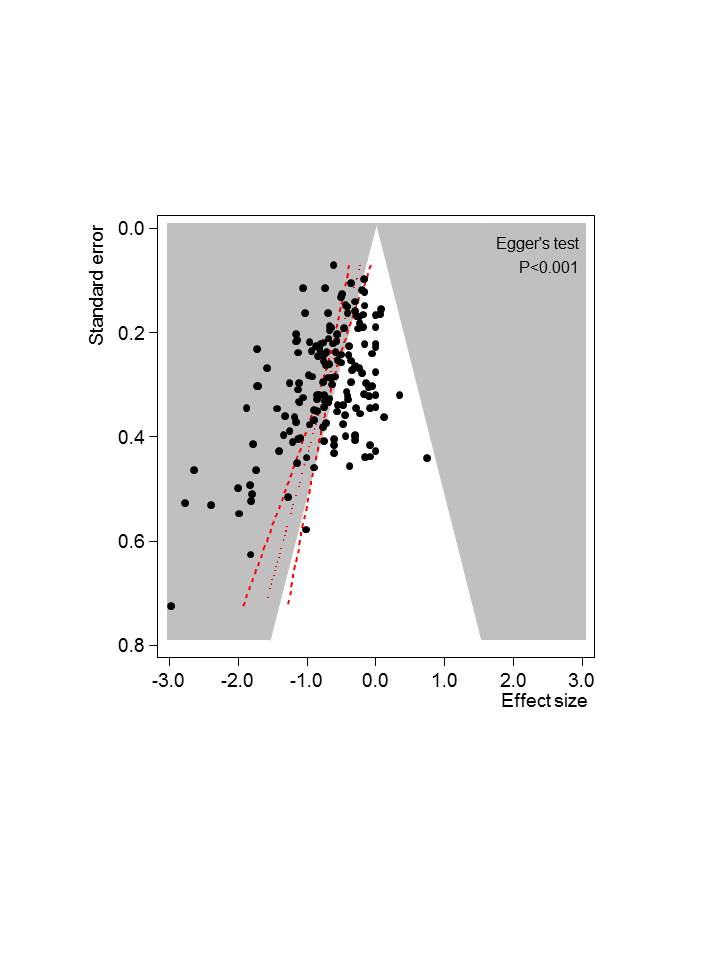


# Figure S2

Funnel plot of studies comparing psychotherapeutic interventions with waitlist, including prediction lines from meta-regression models with the standard error as an explanatory variable and 5% contour areas to display areas of significance and non-significance.

# Figure S3

Forest plot of inconsistency in closed loops with 95% confidence interval (moderately sized studies).

a=waitlist; b=usual care; c=placebo; d=supportive counselling; e=psychodynamic therapy; f=social skill training; g=problem-solving therapy; h=cognitive behavioural therapy; i=behavioural activation; j=interpersonal therapy

# Figure S4

Forest plot of inconsistency in closed loops with 95% confidence interval (large studies).

a=waitlist; b=usual care; c=placebo; d=supportive counselling; e=psychodynamic therapy; f=social skill training; g=problem-solving therapy; h=cognitive behavioural therapy; i=behavioural activation; j=interpersonal therapy
